# Gene Expression Signatures of Radiation Response Are Specific, Durable and Accurate in Mice and Humans

**DOI:** 10.1371/journal.pone.0001912

**Published:** 2008-04-02

**Authors:** Sarah K. Meadows, Holly K. Dressman, Garrett G. Muramoto, Heather Himburg, Alice Salter, ZhengZheng Wei, Geoff Ginsburg, Nelson J. Chao, Joseph R. Nevins, John P. Chute

**Affiliations:** 1 Division of Cellular Therapy, Duke University Medical Center, Durham, North Carolina, United States of America; 2 Department of Molecular Genetics and Microbiology, Duke University Medical Center, Durham, North Carolina, United States of America; 3 Institute for Genome Sciences and Policy, Duke University Medical Center, Durham, North Carolina, United States of America; Deutsches Krebsforschungszentrum, Germany

## Abstract

**Background:**

Previous work has demonstrated the potential for peripheral blood (PB) gene expression profiling for the detection of disease or environmental exposures.

**Methods and Findings:**

We have sought to determine the impact of several variables on the PB gene expression profile of an environmental exposure, ionizing radiation, and to determine the specificity of the PB signature of radiation versus other genotoxic stresses. Neither genotype differences nor the time of PB sampling caused any lessening of the accuracy of PB signatures to predict radiation exposure, but sex difference did influence the accuracy of the prediction of radiation exposure at the lowest level (50 cGy). A PB signature of sepsis was also generated and both the PB signature of radiation and the PB signature of sepsis were found to be 100% specific at distinguishing irradiated from septic animals. We also identified human PB signatures of radiation exposure and chemotherapy treatment which distinguished irradiated patients and chemotherapy-treated individuals within a heterogeneous population with accuracies of 90% and 81%, respectively.

**Conclusions:**

We conclude that PB gene expression profiles can be identified in mice and humans that are accurate in predicting medical conditions, are specific to each condition and remain highly accurate over time.

## Introduction

Invasive procedures are often required for accurate screening and diagnosis of common medical conditions [1]. Examination of the peripheral blood often suffices to establish certain diagnoses, such as chronic lymphocytic leukemia [2], which afflicts the circulating lymphocyte directly. Measurement of total white blood cell counts and the WBC differential (e.g. neutrophils, lymphocytes, monocytes) is routinely performed in medical practice and can facilitate many diagnoses (e.g. bacterial or viral infection). Recently, it has been suggested that gene expression profiling of peripheral blood cells, particularly lymphocytes, can serve as sensitive tool to assess for the presence of certain diseases, such as systemic lupus erythematosus, rheumatoid arthritis, neurologic disease, viral and bacterial infections, breast cancer, atherosclerosis and environmental exposures, including tobacco smoke [3]–[11]. Results from these studies suggest that patterns of gene expression within circulating PB cells can distinguish individuals afflicted by these conditions from those who are not [3]–[11]. It has therefore been suggested that PB gene expression profiling has potential utility in the screening for diseases and environmental exposures. However, any consideration of applying PB gene expression profiles for the detection of disease or environmental exposures requires a determination of the impact of PB cellular composition, time, gender, and genotype on PB gene expression [10]–[13]. Additionally, it is unclear whether PB gene expression profiles that have been associated with various medical conditions are specific for that phenotype, or rather reflect a generalized response to genotoxic stress. Examination of the specificity of PB gene expression profiles in response to different stimuli and the durability of these signatures over time will be critical to allow the translation of this strategy into clinical practice.

Ionizing radiation represents a particularly important environmental hazard, which, at lowest dose exposures, causes little acute health effects [14] and, at higher dose exposures, can cause acute radiation syndrome and death [15]–[17]. Numerous studies have been performed to attempt to understand the biologic effects of ionizing radiation in humans and specific mutations in p53 and HPRT have been identified in somatic cells from survivors of the Hiroshima and Nagasaki atomic bombings [18]–[21]. Gene expression analyses have also been performed on human tumor cells, cell lines, and peripheral blood from small numbers of irradiated patients in order to identify specific genes that are involved in the response to radiation injury [22]–[26]. Recently, public health focus has centered on the development of capabilities to accurately screen large numbers of people for radiation exposure in light of the anticipated use of radiological or nuclear materials by terrorists to produce “dirty bombs” or “improvised nuclear devices” [15]–[17]. We have introduced a method of screening humans for environmental exposure by showing that patterns of gene expression, or metagenes, can be identified in PB cells that accurately distinguish between irradiated and non-irradiated individuals [27]. As importantly, metagenes could be identified in the PB that distinguished different levels of exposure from each other with an accuracy of 96% [27].

In this study, we sought to evaluate the specificity of these PB signatures, as well as to determine the influence of genetic variation and time on the performance of the signature. We conclude that this approach represents a viable strategy for identifying environmental exposures and could be employed for screening populations of affected individuals.

## Methods

### Murine irradiation study

Ten to 11 week old male and female C57Bl6 and female BALB/c mice (Jackson Laboratory, Bar Harbor, ME) were housed at the Duke Cancer Center Isolation Facility under regulations approved by the Duke University Animal Care and Use Committee. Between 5–10 mice/group were given total body irradiation (TBI) with a Cs137 source at an average of 660cGy/min at doses of 50, 200, or 1000cGy as previously described [27]. Six hours, 24 hours, or 7 days post-TBI, approximately 500 ul peripheral blood was collected by retro-orbital bleed from both irradiated and control mice. PB mononuclear cells (PB MNCs) were isolated for total RNA extractions. Total RNA was extracted with Qiagen RNAeasy Mini Kits as previously described [27]. RNA quality was assayed using an Agilent Bioanalyzer 2100 (Agilent Technologies, Inc., Palo Alto, CA).

### Murine LPS study

Ten C57Bl6 female mice were given intraperitoneal injections of a 100 µg of lipopolysaccharide endotoxin (LPS) from *E. coli* 055:B5 (Sigma-Aldrich, St. Louis, MO) to induce sepsis syndrome as previously described [28]. Peripheral blood was collected 6h later from treated and control mice, and RNA was processed as described in the irradiation studies.

### Human Irradiation and Chemotherapy Treatment Studies

With approval from the Duke University Institutional Review Board (IRB), between 5–12 mL of peripheral blood was collected from patients prior to and 6 hrs following total body irradiation with 150 to 200 cGy as part of their pre-transplantation conditioning [27]. For additional comparison, peripheral blood was obtained from healthy volunteers and an additional cohort of patients prior to and 6 hrs following the initiation of alkylator-based chemotherapy alone (without radiotherapy). All patients and healthy volunteers who participated in this study provided written informed consent prior to enrollment, as per the Duke IRB guidelines. PB MNCs and total RNA were isolated from the blood using the identical methods as described for collection of murine cells and RNA.

### DNA Microarrays

Mouse and human oligonucleotide arrays were printed at the Duke Microarray Facility using Operon's Mouse Genome Oligo sets (version 3.0 and version 4.0) and Operon's Human Genome Oligo set (version 3.0 and version 4.0). Data generated from Operon's Mouse and Human version 3 was previously described [27]. Operon's Mouse Genome Oligo set (version 4.0) (https://www.operon.com/arrays/oligosets_mouse.php) contains 35,852 oligonucleotide probes representing 25,000 genes and approximately 38,000 transcripts. Operon's Human Genome Oligo set (version 4.0) (https://www.operon.com/arrays/oligosets_human.php) contains 35,035 oligonucleotide probes, representing approximately 25,100 unique genes and 39,600 transcripts. In order to compare across versions of the Operon oligo sets, Operon provided a map that matched the probes from both versions and only those oligonucleotides that overlapped between versions 3.0 and 4.0 were used in the analysis.

### RNA and Microarray Probe Preparation and Hybridization

Briefly, MNCs were pelleted, and total RNA was isolated using the RNAeasy mini spin column [27]. Total RNA from each sample (mouse or human) and the universal reference RNA (Universal Human or Mouse Reference RNA, Stratagene, http://www.stratagene.com) were amplified and used in probe preparation as previously described [27]. The sample (mouse or human) was labeled with Cy5 and the reference (mouse or human) was labeled with Cy3. The reference RNA allows for the signal for each gene to be normalized to its own unique factor allowing comparisons of gene expression across multiple samples. This serves as a normalization control for two-color microarrays and an internal standardization for the arrays. Amplification, probe preparation and hybridization protocols were performed as previously described [27] and each condition examined had multiple replicates analyzed (n = 3–18 per mouse condition and n = 18–36 per human condition). Detailed protocols are available on the Duke Microarray Facility web site (http://microarray.genome.duke.edu/services/spotted-arrays/protocols).

### Data Processing and Statistical Analysis

Genespring GX 7.3 (Agilent Technologies) was used to perform initial data filtering in which spots whose signal intensities below 70 in either the Cy3 or Cy5 channel were removed. For each analysis, only those samples in that analysis were used in the filtering process. To compare data from previously published results [27], we only used those probes that mapped to each other across the version 3.0 and version 4.0 arrays. To then account for missing values, PAM software (http://www-stat.stanford.edu/tibs/PAM/) was used to impute missing values. *k*-nearest neighbor was used where missing values were imputed using a *k*-nearest neighbor average in gene space. In the analysis approach in which all samples were included, lowess normalization of the data followed by batch effect removal using 2-way mixed model ANOVA (Partek Incorporated) was performed. Gene expression profiles of dose response were used in a supervised analysis using binary regression methodologies as described previously [27]. Prediction analysis of the expression data was performed using MATLAB software as previously described [27]. When predicting levels of radiation exposure, gene selection and identification is based on training the data and finding those genes most highly correlated to response. Each signature summarizes its constituent genes as a single expression profile and is here derived as the first principal component of that set of genes (the factor corresponding to the largest singular value), as determined by a singular value decomposition. Given a training set of expression vectors (of values across metagenes) representing two biological states, a binary probit regression model is estimated using Bayesian methods. Bayesian fitting of binary probit regression models to the training data then permits an assessment of the relevance of the metagene signatures in within-sample classification, and estimation and uncertainty assessments for the binary regression weights mapping metagenes to probabilities of radiation exposure. To internally validate the predictive capacity of the metagene profiles, we performed leave-one-out cross validation studies as we have previously described [27]. A leave one out cross validation involves removing one sample from the dataset, using the remaining samples to develop the model, and then predicting the status of the held out sample. This is then repeated for each sample in the dataset. We have utilized this approach as previously described [27]. A ROC curve analysis was used to define a cut-off for sensitivity and specificity in the predictive models of radiation. Genes found to be predictive of radiation dose were characterized utilizing an in-house program, GATHER (http://meddb01.duhs.duke.edu/gather/). GATHER quantifies the evidence supporting the association between a gene group and an annotation using a Bayes factor [29]. All microarray data files can be found at http://data.cgt.duke.edu/ChuteRadiation.php and at gene expression omnibus website (GEO [http://www.ncbi.nlm.nih.gov/geo], accession number GSE10640).

## Results

### PB gene expression signatures predict ionizing radiation exposure in a heterogeneous population

**Figure 1:**
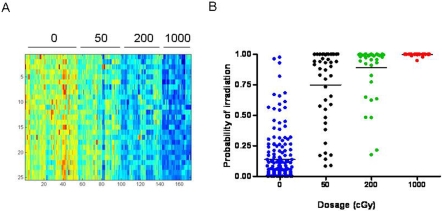
Peripheral blood gene expression profiles distinguish irradiated mice within a heterogeneous population (A) The left panel represents a heat map of a 25 gene profile that can predict radiation status. The figure is sorted by dosage (0 cGy, 50cGy, 200cGy, and 1000cGy). High expression is depicted as red, and low expression is depicted as blue. (B) The right panel is a graph of the predicted capabilities of the irradiation signature across all mice (including C57Bl6 and BALB/c strains, males and females and 3 sampling time points) versus a control, non irradiated sample. All predicted probabilities for the controls are listed.

In a previous study, we demonstrated that PB collected from a single strain and gender of mice, at a single time point, contained patterns of gene expression that predicted both prior radiation exposure and distinguished different levels of radiation exposure with a high degree of accuracy [27]. In this study, we sought to determine whether PB gene expression signatures could be identified that predict radiation exposure status within a population that was heterogeneous for genotype, gender and time of sampling. We found that a clear pattern of gene expression could be identified within this heterogeneous population of mice that distinguished non-irradiated animals from those irradiated with 50 cGy, 200 cGy, and 1000 cGy (Figure 1*A*). To verify that these patterns did indeed represent genes reflecting exposure to radiation, we utilized a leave-one-out cross-validation analysis to assess the ability of the pattern to predict the relevant samples (Figure 1*B*). The results demonstrate that the pattern selected for distinguishing control animals from those irradiated at various doses has the capacity to predict the status of the samples. The accuracies of prediction of the non-irradiated samples, the 50 cGy-, 200 cGy- and 1000 cGy-irradiated samples were 92%, 78%, 91% and 100%, respectively.

### Sex differences impact the accuracy of gene expression signatures of radiation

**Figure 2:**
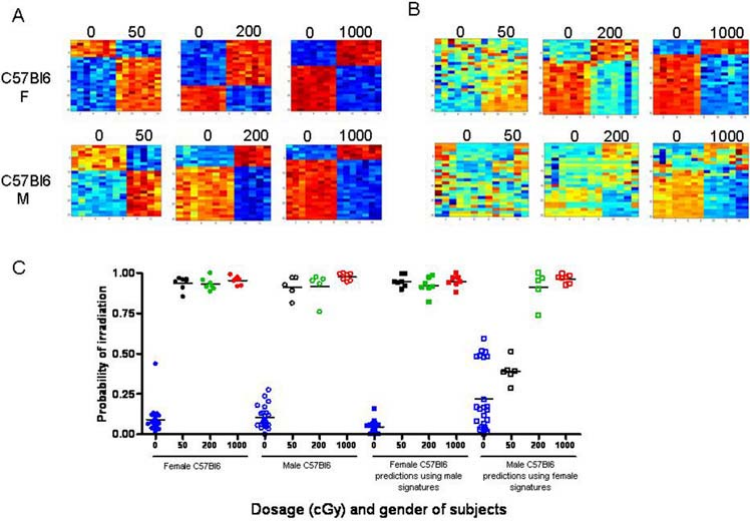
Impact of sex on murine irradiation profiles (A) Heat map images illustrating expression pattern of genes selected for classifying control, non-irradiated mice versus 50 cGy, 200 cGy, or 1000 cGy irradiated mice within female (top) and male C57Bl6 mice (bottom). (B) Heat map images illustrating expression pattern of genes found in the female C57Bl6 strain or male C57Bl6 strain predicting the irradiation status of the opposite sex at dosage 50 cGy, 200 cGy, 1000 cGy. High expression is depicted as red, and low expression is depicted as blue. (C) A leave-one-out cross-validation analysis of the classification for control (blue) versus 50 cGy (black), 200 cGy (green), and 1000 cGy (red) for the female C57Bl6 (squares) and male C57Bl6 (circles) samples is shown. The control probabilities for each prediction are shown.

We next sought to determine the extent to which variables within a heterogeneous population can limit the accuracy of PB gene expression profiling. In order to address the impact of sex difference, healthy adult male and female C57Bl6 mice were irradiated with 50 cGy, 200 cGy, and 1000 cGy and PB was collected at 6 hours post-irradiation, along with PB from non-irradiated control mice (n = 7–10 per group). Patterns of gene expression could be identified in the PB of both male and female mice that appeared to distinguish radiation exposure status (Figure 2*A*). When the PB signatures from the male C57Bl6 mice were tested against the female PB samples, the heat map analysis suggested less distinction between the non-irradiated and irradiated profiles (Figure 2*B*). Comparable effects were observed when the female PB signatures were applied against male PB samples. A leave-one-out cross-validation analysis demonstrated that the male and female PB signatures of radiation were 100% accurate in predicting the radiation status of PB samples from mice of the same sex (Figure 2*C*). The male PB signatures also were 100% accurate in predicting the status of the female mice. However, the female PB signatures were less accurate in distinguishing the non-irradiated from 50 cGy irradiated male mice, with improved accuracy in predicting non-irradiated samples from male mice irradiated with higher doses of radiation (200 cGy and 1000 cGy; Figure 2*C*). The basis for the observed differences in predicting the radiation status of mice across gender differences may be a function of the distinct sets of genes which are represented in the predictors of radiation exposure in males and females (Table S1). Less than 15% of the genes overlapped between the PB metagenes of males and females at each dose of radiation (Table S2).

### Impact of genotype on prediction of radiation status

**Figure 3:**
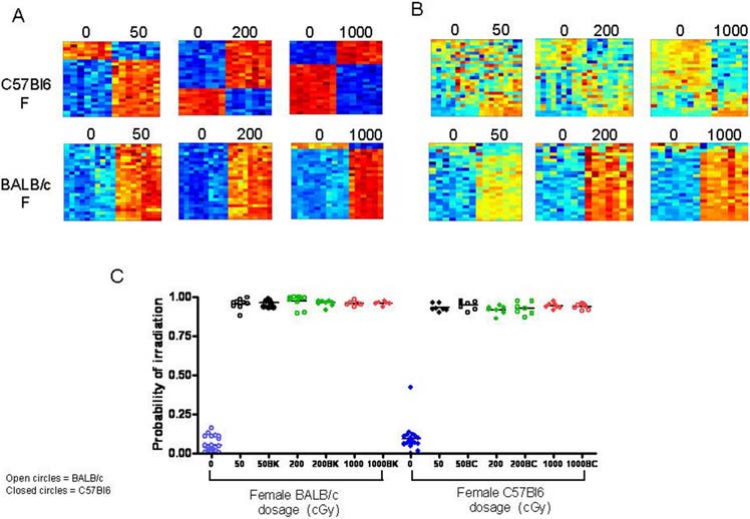
Impact of genotype on murine irradiation profiles. (A) Heat map images illustrating expression pattern of genes selected for classifying control, non-irradiated samples versus 50 cGy, 200 cGy, 1000 cGy irradiated samples between female C57Bl6 strain (top) and female BALB/c strain (bottom). (B) Heat map images illustrating expression pattern of genes developed in one strain as predicting the other strain (C57Bl6 or BALB/c). High expression is depicted as red and low expression is depicted as blue. (C) A leave-one-out cross-validation analysis of the classification for control versus 50 cGy (black), 200 cGy (green), and 1000 cGy (red) for the female BALB/c and female C57Bl6 samples is shown. The control probabilities for each prediction are shown. BK represents the application of female C57Bl6 metagenes to predict the status of female BALB/c mice, and BC represents using female BALB/c mice metagenes to predict the status of female C57Bl6 mice.

Since the human population is genetically diverse, we next examined whether gene expression signatures of radiation exposure could accurately predict the status of mice across different genotypes. PB was collected from C57Bl6 and BALB/c mice at 6 hours following 50 cGy, 200 cGy or 1000 cGy and we were able to identify patterns of gene expression which appeared to distinguish the different levels of radiation from the non-irradiated controls within each strain (Figure 3*A*). However, when the PB gene expression signatures from C57Bl6 mice were tested against BALB/c mice, and vice versa, the gene expression profiles were less distinct (Figure 3*B*). We then performed a leave-one-out cross-validation analysis in which gene expression profiles from C57Bl6 mice were tested against PB from BALB/c mice and found that the metagene predictors of radiation from C57Bl6 mice displayed 100% accuracy in predicting the status of non-irradiated and irradiated BALB/c mice (Figure 3*C*). Similarly, application of the PB metagene profiles of radiation generated in BALB/c mice demonstrated 100% accuracy in distinguishing non-irradiated and irradiated C57Bl6 mice. Interestingly, less than 20% of the genes represented within the PB predictors from C57Bl6 mice and BALB/c mice overlapped (Table S3, Table S2), but both predictors were highly accurate in predicting the radiation status of the different strain of mice. Dda3, a p53-inducible gene, which participates in suppression of cell growth [30], was represented in both strains at all radiation doses.

### The impact of time on PB gene expression signatures of irradiation

**Figure 4:**
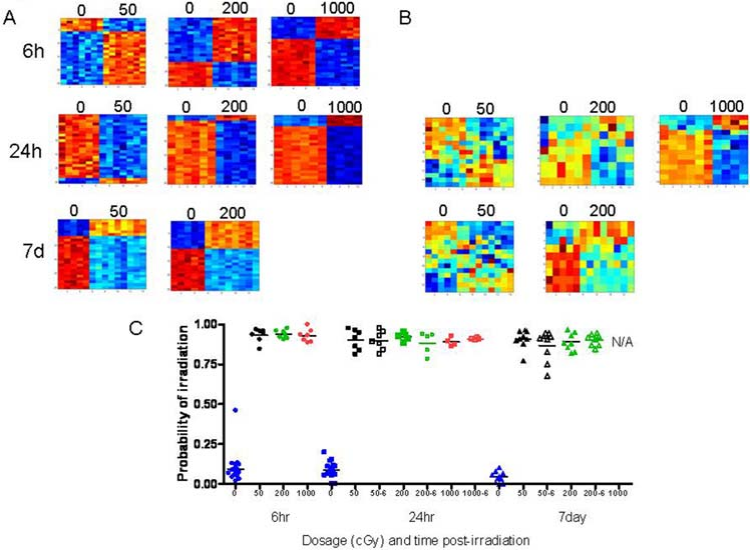
Impact of time on murine irradiation profiles. (A) Heat map images illustrating expression pattern of genes selected for classifying control, non-irradiated samples versus 50 cGy, 200 cGy, 1000 cGy irradiated samples at time points 6hr, 24hr, and 7days. (B) Heat map images illustrating expression pattern of genes found in the 6hr time point as applied to the dosages 50 cGy, 200 cGy, 1000 cGy at 24 hr and 7 day time points. High expression is depicted as red, and low expression is depicted as blue. (C) A leave-one-out cross-validation analysis of the classification for control (blue) versus 50 cGy (black), 200 cGy (green), and 1000 cGy (red) for the time points 6 hr (circles), 24 hr (squares), and 7 days (triangles) is shown. The control probabilities for each prediction are shown.

PB responses to environmental exposures may change over time as a function of changes in PB cellular composition and cellular responses themselves. We identified patterns of gene expression in the PB of C57Bl6 female mice at 6 hrs, 24 hrs and 7 days post-irradiation which appeared to distinguish the 3 different levels of radiation versus non-irradiated mice (Figure 4*A*). When the PB metagene profiles of radiation exposure generated from the 6 hr time point were applied against PB samples from mice at the 24 hr and 7 day time points post-irradiation, the profiles appeared less distinct (Figure 4*B*). A leave-one-out cross-validation analysis demonstrated that the PB metagene profiles from each time point predicted each dose of radiation with 100% accuracy (Figure 4*C*). Next, a leave-one-out cross-validation analysis was performed using the metagene profiles from the 6 hr time point against each of the PB samples from mice at 24 hr and 7 day time points and the 6 hr metagene profiles demonstrated 100% accuracy in predicting the radiation status of the 24 hr and 7 day time point samples (Figure 4*C*). Of note, the 7 day time point following 1000 cGy exposure could not be analyzed since we were unable to collect sufficient RNA from these PB samples to allow gene array hybridization to be performed. Although we found that time did not impact the accuracy of PB gene expression profiles in predicting radiation status, the lists of genes which comprised these PB signatures changed significantly over 7 days (Table S4). No genes were found in common between the 6 hr predictors and the 24 hr or 7 day PB signatures of radiation in 50 cGy-, 200 cGy-, or 1000 cGy-treated mice (Table S2). A single gene, Galectin 1 (Lgals1), a carbohydrate binding protein that is involved in the induction of cell death [31], was found in common between the 24 hr and 7 day predictors of 50 cGy.

### Specificity of PB signatures

**Figure 5:**
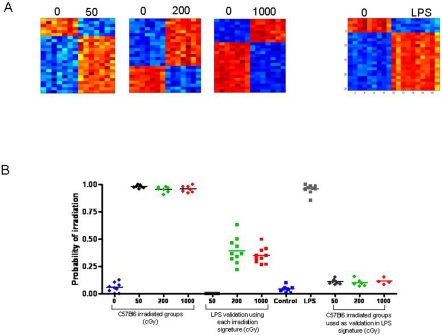
Peripheral blood profiles of irradiation and LPS-treatment are highly specific. (A) Heat maps representing unique metagene profiles are shown which were utilized to distinguish 3 different levels of irradiation (left) and to distinguish LPS-treatment (right) in C57Bl6 mice. (B) The graph at left represents the predictive capabilities of the PB irradiation signatures in the female C57Bl6 mice in predicting dosage profiles at 50 cGy (black), 200 cGy (green), and 1000 cGy (red); the middle graph represents the predictive capabilities of the irradiation signatures when validated against the LPS-treated samples (squares); at right, the LPS signature was validated against the C57Bl6 irradiated mice and the predicted probabilities for 50 cGy (black), 200 cGy (green), and 1000 cGy (red) are shown.

In addition to inter-individual variations [12], human populations are heterogeneous with respect to health status and medical conditions. Therefore, it is critical to determine whether PB gene expression profiles of radiation response are specific to radiation exposure itself or whether these signatures are potentially confounded by other genotoxic stresses. We chose to compare the PB gene expression response to ionizing radiation exposure with that of gram-negative bacterial sepsis, since this syndrome can be expected to induce similar multiorgan toxicity as is observed following radiation injury [15]–[17], [32]. A pattern of gene expression could be identified which effectively distinguished female C57Bl6 mice treated with Escherichia coli-derived lipopolysaccharide (LPS), experiencing sepsis syndrome, from untreated female C57Bl6 mice (Figure 5*A*). Applying a leave-one-out cross-validation analysis, we found that the PB signature for 50 cGy irradiation in C57Bl6 mice correctly predicted the status of all LPS-treated C57Bl6 mice as non-irradiated, suggesting robust specificity of the signature for low level (50 cGy) irradiation and sepsis syndrome (Figure 5*B*). The PB signatures for 200 cGy and 1000 cGy also correctly predicted the LPS-treated mice as non-irradiated, although these probabilities were less robust than the application of the 50 cGy signature (Figure 5*B*). The PB signature of LPS-treatment also correctly predicted the status of all irradiated mice as “non-LPS treated” (Figure 5*B*, right). These data indicate that the PB gene expression profiles of radiation response and bacterial sepsis are quite specific and able to distinguish one condition from the other with a high level of accuracy. No overlap was observed between the genes which comprised the PB signature of LPS-sepsis and the PB signatures of radiation exposure in C57Bl6 mice (Table S5).

### PB signatures of radiation and chemotherapy are specific in humans

**Figure 6:**
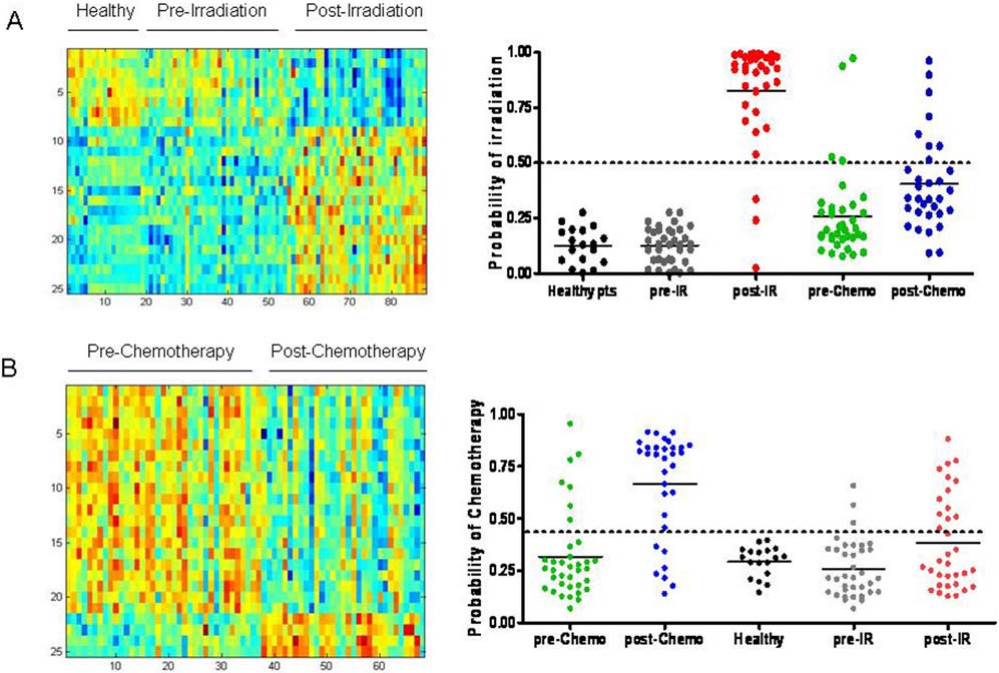
PB metagene profiles of human radiation exposure and chemotherapy treatment are accurate and specific relative to each other. (A) The heat map on the left depicts the expression profiles of genes (rows) selected to discriminate the human samples (columns); high expression is depicted as red, and low expression is depicted as blue. A leave-one-out cross-validation assay (at right) demonstrated that the PB metagene of radiation was capable of distinguishing healthy donors (black), non-irradiated patients (gray), irradiated patients (red), pre-chemotherapy treatment patients (green), and post-chemotherapy patients (blue). A ROC curve analysis was used to define a cut-off for sensitivity and specificity of the predictive model of radiation. The dotted line represents this threshold of sensitivity and specificity. (B) The heatmap on the left depicts an expression profile of chemotherapy treatment that distinguishes chemotherapy-treated versus untreated patients. A leave-one-out cross-validation assay demonstrated that this PB metagene of chemotherapy treatment could accurately distinguish pre-chemotherapy patients (green), chemotherapy-treated patients (blue), healthy individuals (black), pre-irradiated patients (gray) and irradiated patients (red).

In order to extend the analysis of PB signature specificity to humans, we collected PB from a population of healthy individuals (n = 18), patients who had undergone total body irradiation as conditioning prior to hematopoietic stem cell transplantation (n = 47) and patients who had undergone alkylator-based chemotherapy conditioning alone (n = 41). RNA of sufficient quality was available from 18 healthy donor samples, 36 pre-irradiated patients, 34 post-irradiated patients, 36 pre-chemotherapy treatment patients and 32 post-chemotherapy patients (Table S6). A supervised binary regression analysis identified a metagene profile of 25 genes that distinguished the healthy individuals and the non-irradiated patients from the irradiated patients (Figure 6*A*). A leave-one-out cross validation analysis demonstrated that this PB predictor of human radiation response was 100% accurate in predicting the healthy individuals and the non-irradiated patients and 91% accurate at predicting the irradiated patients (Figure 6*A*).

In order to test the specificity of this PB signature of human radiation response, we next tested its accuracy in predicting the status of patients who had undergone chemotherapy treatment alone. This signature correctly predicted 89% of the non-irradiated, pre-chemotherapy patients as non-irradiated and 75% of the chemotherapy-treated patients as non-irradiated (Figure 6*A*). Interestingly, 2 of the post-chemotherapy patients had a prior history of total lymphoid irradiation and both of these were mispredicted as “irradiated”, suggesting perhaps that a durable molecular response to radiation was evident in these patients. Considering the entire population, the overall accuracy of the PB predictor of radiation was 90%. Within the chemotherapy-treated patients, a PB signature could be identified that appeared to distinguish untreated patients from chemotherapy-treated patients (Figure 6*B*). A leave-one-out cross-validation analysis demonstrated that this PB signature of chemotherapy treatment was 81% accurate at distinguishing the untreated patients and 78% accurate at predicting the chemotherapy-treated patients (Figure 6*B*). Furthermore, the chemotherapy metagene profile demonstrated 100% accuracy in predicting the status of healthy individuals, 92% accuracy in predicting the non-irradiated patients, and 62% accuracy in predicting the PB samples from irradiated patients as not having received chemotherapy (Figure 6*B*). The overall accuracy of the PB predictor of chemotherapy-treatment was 81%. Interestingly, no overlapping genes were identified between the PB signature of radiation and the PB signature of chemotherapy treatment (Tables S7 and S8). It is also worth noting that all 12 of the post-irradiation patients whose status was mispredicted by the PB chemotherapy signature had received prior chemotherapy in the treatment of their underlying disease.

## Discussion

Numerous studies now highlight the power of gene expression profiling to characterize the biological phenotype of complex diseases. We and others have shown the potential clinical utility of gene expression profiles in cancer research, in which the identification of patterns of gene expression within tumors has led to the characterization of tumor subtypes, prognostic categories and prediction of therapeutic response [33]–[37]. Beyond analysis of tumor tissues, it has also been suggested that gene expression profiling of the peripheral blood can provide indication of infections, cancer, heart disease, allograft rejection, environmental exposures and as a means of biological threat detection [3]–[11], [38], [39]. While the concept of PB cells as sentinels of disease is not new, it remains unclear whether PB gene expression profiles that have been associated with various conditions are specific for those diseases or rather reflect a common molecular response to a variety of genotoxic stresses. Given the dynamic nature of the cellular composition of PB blood [12] and the complexity of cellular responses over time [12], the durability of PB signatures over time is also uncertain and could affect the diagnostic utility of this approach for public health screening.

We sought to address the capacity for PB gene expression profiles to distinguish an environmental exposure, in this case ionizing radiation, versus other medical conditions and to examine the impact of time, gender and genotype on the accuracy of these profiles. We found that PB gene expression signatures can be identified which accurately predict irradiated from non-irradiated mice and distinguish different levels of radiation exposure, all within a heterogeneous population with respect to gender, genotype and time from exposure. These results suggest the potential for PB gene expression profiling to be applied successfully in the screening for an environmental exposure. Previous studies have indicated that inter-individual variation in gene expression occurs within healthy individuals [12] and may therefore limit the accuracy of PB gene expression profiling to detect diseases or exposures. Our results demonstrate that the environmental exposure tested here, ionizing radiation, induced a pronounced and characteristic alteration in PB gene expression such that a PB expression profile was highly predictive of radiation status in a population with variable gender, genotype and time of analysis. From a practical standpoint, these data suggest the potential utility of this approach for biodosimetric screening of a heterogeneous human population in the event of a purposeful or accidental radiological or nuclear event [15]–[17].

This study revealed that sex differences can impact the accuracy of this approach, particularly in distinguishing mice exposed to lower dose irradiation from non-irradiated controls. These results imply that aspects of the PB response to ionizing radiation are specified by sex-associated genes. Whitney et al. [12] previously showed that sex differences were associated with variation in PB autosomal gene expression in healthy individuals. Our study suggests that sex differences may contribute to characteristically distinct PB molecular responses to environmental stress (radiation) and the accuracy of PB gene expression profiling for medical screening can be affected by sex. These sex-related differences in PB response to ionizing radiation are perhaps illustrated by the fact that only 2 genes overlapped between the male and female PB signatures of 50 cGy (Ccng1 and Dda3).

Interestingly, differences in genotype did not significantly impact the accuracy of the PB gene expression signatures to distinguish radiation response such that PB signatures from C57Bl6 mice displayed 100% accuracy in predicting the status of BALB/c mice and vice versa. This observation demonstrates that, while genotype differences can account for some variation in PB gene expression [12], the alterations in PB gene expression induced by 3 different levels of radiation exposure are such that PB expression profiling is highly accurate in distinguishing all irradiated mice across different genotypes. Very few genes were found in common between the 2 strains of mice at each level of radiation exposure, indicating that diverse sets of genes contribute to the PB response to radiation and that unique sets of genes can be identified which are predictive of radiation response.

The time of PB collection following radiation exposure had no significant impact on the accuracy of PB signatures to predict radiation status or distinguish different levels of exposure. First, the accuracy of PB signatures to predict radiation status and distinguish different levels of radiation exposure did not decay over time. Second, when we applied a PB signature from a single time point (6 hrs) against PB samples collected from mice at other time points (24 hr and 7 days), the accuracy of the prediction remained 100% in all cases. Therefore, time as a single variable did not lessen the accuracy of this approach to distinguish irradiated from non-irradiated animals. However, the content of the genes which comprised the PB signatures changed significantly as a function of time and <20% of the genes overlapped between the PB signatures of radiation at 6 hr, 24 hr, and 7 days. Taken together, these data indicate that PB predictors of radiation response do change over time, but PB signatures can continuously be identified through 7 days that are highly accurate at predicting radiation status and distinguishing different levels of radiation exposure. From a practical perspective, these results suggest that the application of a single reference set of “radiation response” genes would be unlikely to provide the most sensitive screen for radiation exposure over time. Conversely, reference lists of PB genes that are specific for different time points could be applied in the screening for radiation exposure provided that the time of exposure was known.

A critical question to be addressed in the development of PB gene expression profiling to detect medical conditions or exposures is the specificity of PB gene expression changes in response to genotoxic stresses. The PB signatures of 3 different doses of radiation displayed 100% accuracy in identifying septic animals as non-irradiated and the PB signature of sepsis was also 100% accurate in identifying irradiated mice as non-septic. These results demonstrate specificity in the PB responses to ionizing radiation and sepsis. These data also provide *in vivo* validation of a prior report by Boldrick et al. [40] in which human PB mononuclear cells were found to have a stereotypic response to LPS exposure in vitro and specific alterations in gene expression were observed in response to different strains of bacteria [40]. Ramilo et al. also recently reported that distinct patterns of PB gene expression can be identified among patients with different bacterial infections [11]. We found no genes in common between the PB signatures of radiation exposure and the PB signature of gram negative sepsis. Taken together, our results demonstrate that the *in vivo* PB molecular responses to ionizing radiation and bacterial sepsis are quite distinct and can be utilized to distinguish one condition from the other with a high level of accuracy.

Our analyses of expression signatures in human patients demonstrated that it is possible to utilize PB gene expression profiles to distinguish individuals who have been exposed to an environmental hazard, ionizing radiation, within a heterogeneous human population with a high level of accuracy. It will be important to further test the accuracy of this PB predictor of human radiation exposure in a human population exposed to lower dose irradiation (e.g. 0.1–1 cGy), as might be expected via occupational exposures (e.g. radiology technicians, nuclear power plant workers)[41]–[43]. A potential pitfall in the clinical application of PB gene expression profiling would be that variations in PB gene expression in people would be such that it might be difficult to distinguish the effects of a given exposure or medical condition from expected background alterations in gene expression [12]. However, Whitney et al. [12] showed that the alterations in PB gene expression observed in patients with lymphoma or bacterial infection was significantly greater than the relatively narrow variation observed in healthy individuals [12]. Our study confirms that PB gene expression profiles can be successfully applied to detect a specific exposure in a heterogeneous human population and that inter-individual differences in PB gene expression do not significantly confound the utility of this approach.

We further showed that unique PB gene expression profiles can be identified which distinguish chemotherapy-treated patients versus patients who had not received chemotherapy with an overall accuracy of 81% and 78%, respectively. Similar to the PB signature of radiation, the PB signature of chemotherapy demonstrated accuracy and specificity in distinguishing healthy individuals and pre-irradiated patients (100% and 92% accuracy, respectively). However, the accuracy of the PB signature of chemotherapy was more limited when tested against patients who received radiation conditioning (62%). This observation provides the basis for further investigation as to which families of genes may be represented in both the PB molecular response to radiation and chemotherapy. However, since all 12 of the post-irradiation patients whose status was mispredicted by the PB chemotherapy signature had received combination chemotherapy within the prior year, the true specificity of this PB signature of chemotherapy cannot be addressed via this comparison. We are currently enrolling additional patients to this study who have not undergone prior chemotherapy to further test the specificity of a PB metagene of chemotherapy treatment.

Peripheral blood is a readily accessible source of tissue which has the potential to provide a window to the presence of disease or exposures. Early studies applying PB gene expression analysis have demonstrated that this approach is sensitive for the detection of patterns of gene expression in association with a variety of medical conditions [3]–[12], [27]. It remains to be determined whether PB gene expression profiles can be successfully applied in medical practice or public health screening for the early detection of specific diseases or environmental exposures. Our results demonstrate that PB gene expression profiles can be identified in mice and humans which are specific, accurate over time, and not confounded by inter-individual differences.

## Supporting Information

Table S1 (0.43 MB DOC)

Table S2 (0.09 MB DOC)

Table S3 (0.20 MB DOC)

Table S4 (0.49 MB DOC)

Table S5 (0.08 MB DOC)

Table S6 (0.03 MB DOC)

Table S7 (0.08 MB DOC)

Table S8 (0.08 MB DOC)
