# Correction: Gene Expression Signatures of Radiation Response Are Specific, Durable and Accurate in Mice and Humans

**DOI:** 10.1371/annotation/83c37842-8867-4cfb-a271-9e4bf36fb9b8

**Published:** 2008-05-13

**Authors:** Sarah K. Meadows, Holly K. Dressman, Garrett G. Muramoto, Heather Himburg, Alice Salter, ZhengZheng Wei, Geoffrey S. Ginsburg, Nelson J. Chao, Joseph R. Nevins, John P. Chute

The seventh author's name appears incorrectly. It should be: Geoffrey S. Ginsburg

