# Supplementary material for: Gene Expression Signatures of Radiation Response Are Specific, Durable and Accurate in Mice and Humans
Source: PLoS One. 2008 Apr 2;3(4):e1912. doi: 10.1371/journal.pone.0001912 (PMC2271127; doi:10.1371/journal.pone.0001912)
Supplement: Table S1 — (0.43 MB DOC) [file pone.0001912.s001.doc]

Table S1. Genes that distinguish radiation responses in male and female C57Bl6 mice. Operon Oligo ID can be queried in the OMAD database ([http://omad.operon.com](http://omad.operon.com/))

| **Operon OligoID** | **Gene Symbol** | **RefSeq** | **Genbank** | **Description** |
| --- | --- | --- | --- | --- |
| **MALES** |  |  |  |  |
| **50 Gy** |  |  |  |  |
| [M200013484](http://omad.operon.com/mouseV3/transcript.php?what=M200013484) | [9030617O03Rik](http://www.informatics.jax.org/searches/accession_report.cgi?id=MGI%3A2444813) | [NM_145448](http://srs.sanger.ac.uk/srsbin/cgi-bin/wgetz?-e+%5BREFSEQ-ID:NM_145448%5D) | [BC021385](http://www.ebi.ac.uk/cgi-bin/emblfetch?BC021385) | - |
| [M200000800](http://omad.operon.com/mouseV3/transcript.php?what=M200000800) | [Ccng1](http://www.informatics.jax.org/searches/accession_report.cgi?id=MGI%3A102890) | [NM_009831](http://srs.sanger.ac.uk/srsbin/cgi-bin/wgetz?-e+%5BREFSEQ-ID:NM_009831%5D) | [AB005559](http://www.ebi.ac.uk/cgi-bin/emblfetch?AB005559) | CYCLIN G1 (CYCLIN G] |
| [M200004687](http://omad.operon.com/mouseV3/transcript.php?what=M200004687) | [Dda3-pending](http://www.informatics.jax.org/searches/accession_report.cgi?id=MGI%3A1913099) | [NM_019976](http://srs.sanger.ac.uk/srsbin/cgi-bin/wgetz?-e+%5BREFSEQ-ID:NM_019976%5D) | [AK041835](http://www.ebi.ac.uk/cgi-bin/emblfetch?AK041835) | DIFFERENTIAL DISPLAY AND ACTIVATED BY P53; P53-REGULATED DDA3. |
| [M200003784](http://omad.operon.com/mouseV3/transcript.php?what=M200003784) | [Bax](http://www.informatics.jax.org/searches/accession_report.cgi?id=MGI%3A99702) | [NM_007527](http://srs.sanger.ac.uk/srsbin/cgi-bin/wgetz?-e+%5BREFSEQ-ID:NM_007527%5D) | [L22472](http://www.ebi.ac.uk/cgi-bin/emblfetch?L22472) | APOPTOSIS REGULATOR BAX, MEMBRANE ISOFORM ALPHA. |
| [M200007794](http://omad.operon.com/mouseV3/transcript.php?what=M200007794) | [Wig1](http://www.informatics.jax.org/searches/accession_report.cgi?id=MGI%3A1195270) | [NM_009517](http://srs.sanger.ac.uk/srsbin/cgi-bin/wgetz?-e+%5BREFSEQ-ID:NM_009517%5D) | [AF012923](http://www.ebi.ac.uk/cgi-bin/emblfetch?AF012923) | WILD-TYPE P53-INDUCED GENE 1. |
| [M200016031](http://omad.operon.com/mouseV3/transcript.php?what=M200016031) | [Polk](http://www.informatics.jax.org/searches/accession_report.cgi?id=MGI%3A1349767) | [NM_012048](http://srs.sanger.ac.uk/srsbin/cgi-bin/wgetz?-e+%5BREFSEQ-ID:NM_012048%5D) | [AB040764](http://www.ebi.ac.uk/cgi-bin/emblfetch?AB040764) | POLYMERASE (DNA DIRECTED), KAPPA; DINB HOMOLOG 1 (E. COLI); DNA DAMAGE-INDUCIBLE PROETIN B; DNA DAMAGE-INDUCIBLE PROTEIN B; POLYMERASE (DNA DIRECTED) KAPPA. |
| [M200000935](http://omad.operon.com/mouseV3/transcript.php?what=M200000935) | [Gcdh](http://www.informatics.jax.org/searches/accession_report.cgi?id=MGI%3A104541) | [NM_008097](http://srs.sanger.ac.uk/srsbin/cgi-bin/wgetz?-e+%5BREFSEQ-ID:NM_008097%5D) | [U18992](http://www.ebi.ac.uk/cgi-bin/emblfetch?U18992) | GLUTARYL-COA DEHYDROGENASE, MITOCHONDRIAL PRECURSOR (EC 1.3.99.7) (GCD). |
| [M300010491](http://omad.operon.com/mouseV3/transcript.php?what=M300010491) | [D030041N15Rik](http://www.informatics.jax.org/searches/accession_report.cgi?id=MGI%3A2443767) | [NM_153416](http://srs.sanger.ac.uk/srsbin/cgi-bin/wgetz?-e+%5BREFSEQ-ID:NM_153416%5D) | [BC018191](http://www.ebi.ac.uk/cgi-bin/emblfetch?BC018191) | ALADIN (ADRACALIN).] |
| [M200003481](http://omad.operon.com/mouseV3/transcript.php?what=M200003481) | [2210412K09Rik](http://www.informatics.jax.org/searches/accession_report.cgi?id=MGI%3A1924209) | [NM_029814](http://srs.sanger.ac.uk/srsbin/cgi-bin/wgetz?-e+%5BREFSEQ-ID:NM_029814%5D) | [BC006947](http://www.ebi.ac.uk/cgi-bin/emblfetch?BC006947) | -- |
| [M200006137](http://omad.operon.com/mouseV3/transcript.php?what=M200006137) | [Stinp](http://www.informatics.jax.org/searches/accession_report.cgi?id=MGI%3A1926609) | [NM_021897](http://srs.sanger.ac.uk/srsbin/cgi-bin/wgetz?-e+%5BREFSEQ-ID:NM_021897%5D) | [AY034612](http://www.ebi.ac.uk/cgi-bin/emblfetch?AY034612) | STRESS INDUCED PROTEIN; THYMUS EXPRESSED ACIDIC PROTEIN. |
| [M300008376](http://omad.operon.com/mouseV3/transcript.php?what=M300008376) | [Pon2](http://www.informatics.jax.org/searches/accession_report.cgi?id=MGI%3A106687) | [NM_008896](http://srs.sanger.ac.uk/srsbin/cgi-bin/wgetz?-e+%5BREFSEQ-ID:NM_008896%5D) | [L48514](http://www.ebi.ac.uk/cgi-bin/emblfetch?L48514) | SERUM PARAOXONASE/ARYLESTERASE 2 (EC 3.1.1.2) (EC 3.1.8.1) (PON 2) (SERUM ARYLDIAKYLPHOSPHATASE 2) (A-ESTERASE 2) (AROMATIC ESTERASE 2).] |
| [M200006229](http://omad.operon.com/mouseV3/transcript.php?what=M200006229) | [Dstn](http://www.informatics.jax.org/searches/accession_report.cgi?id=MGI%3A1929270) | [NM_019771](http://srs.sanger.ac.uk/srsbin/cgi-bin/wgetz?-e+%5BREFSEQ-ID:NM_019771%5D) | [AB025406](http://www.ebi.ac.uk/cgi-bin/emblfetch?AB025406) | DESTRIN (ACTIN-DEPOLYMERIZING FACTOR) (ADF). |
| [M300013831](http://omad.operon.com/mouseV3/transcript.php?what=M300013831) | [Myo15](http://www.informatics.jax.org/searches/accession_report.cgi?id=MGI%3A1261811) | [NM_010862](http://srs.sanger.ac.uk/srsbin/cgi-bin/wgetz?-e+%5BREFSEQ-ID:NM_010862%5D) | [AB014510](http://www.ebi.ac.uk/cgi-bin/emblfetch?AB014510) | MYOSIN XV (UNCONVENTIONAL MYOSIN-15). |
| [M200009374](http://omad.operon.com/mouseV3/transcript.php?what=M200009374) | [2310045N01Rik](http://www.informatics.jax.org/searches/accession_report.cgi?id=MGI%3A1919618) | [NM_008578](http://srs.sanger.ac.uk/srsbin/cgi-bin/wgetz?-e+%5BREFSEQ-ID:NM_008578%5D) | [AK009829](http://www.ebi.ac.uk/cgi-bin/emblfetch?AK009829) | MYOCYTE-SPECIFIC ENHANCER FACTOR 2B. |
| [M200015906](http://omad.operon.com/mouseV3/transcript.php?what=M200015906) | [5530601I19Rik](http://www.informatics.jax.org/searches/accession_report.cgi?id=MGI%3A1918698) | [NM_027797](http://srs.sanger.ac.uk/srsbin/cgi-bin/wgetz?-e+%5BREFSEQ-ID:NM_027797%5D) | [BC022756](http://www.ebi.ac.uk/cgi-bin/emblfetch?BC022756) | -- |
| [M200004993](http://omad.operon.com/mouseV3/transcript.php?what=M200004993) | [Ifi47](http://www.informatics.jax.org/searches/accession_report.cgi?id=MGI%3A99448) | [NM_008330](http://srs.sanger.ac.uk/srsbin/cgi-bin/wgetz?-e+%5BREFSEQ-ID:NM_008330%5D) | [M63630](http://www.ebi.ac.uk/cgi-bin/emblfetch?M63630) | INTERFERON GAMMA INDUCIBLE PROTEIN; INTERFERON GAMMA INDUCIBLE PROTEIN, 47 KDA |
| [M200006667](http://omad.operon.com/mouseV3/transcript.php?what=M200006667) | [D11Ertd619e](http://www.informatics.jax.org/searches/accession_report.cgi?id=MGI%3A1277172) | [NM_026538](http://srs.sanger.ac.uk/srsbin/cgi-bin/wgetz?-e+%5BREFSEQ-ID:NM_026538%5D) | [AK011136](http://www.ebi.ac.uk/cgi-bin/emblfetch?AK011136) | PROBABLE ATP-DEPENDENT 61 KDA NUCLEOLAR RNA HELICASE. |
| [M200013613](http://omad.operon.com/mouseV3/transcript.php?what=M200013613) | [Gnrpx-pending](http://www.informatics.jax.org/searches/accession_report.cgi?id=MGI%3A1925920) | -- | [BC005565](http://www.ebi.ac.uk/cgi-bin/emblfetch?BC005565) | -- |
| [M300020474](http://omad.operon.com/mouseV3/transcript.php?what=M300020474) | -- | -- | -- | -- |
| [M200004237](http://omad.operon.com/mouseV3/transcript.php?what=M200004237) | [Ris2](http://www.informatics.jax.org/searches/accession_report.cgi?id=MGI%3A1914427) | [NM_026014](http://srs.sanger.ac.uk/srsbin/cgi-bin/wgetz?-e+%5BREFSEQ-ID:NM_026014%5D) | [AK028287](http://www.ebi.ac.uk/cgi-bin/emblfetch?AK028287) | RETROVIRAL INTEGRATION SITE 2; RETROVIRAL INTEGRATION SITE 1. |
| [M200005712](http://omad.operon.com/mouseV3/transcript.php?what=M200005712) | [Hexb](http://www.informatics.jax.org/searches/accession_report.cgi?id=MGI%3A96074) | [NM_010422](http://srs.sanger.ac.uk/srsbin/cgi-bin/wgetz?-e+%5BREFSEQ-ID:NM_010422%5D) | [U07741](http://www.ebi.ac.uk/cgi-bin/emblfetch?U07741) | BETA-HEXOSAMINIDASE BETA CHAIN PRECURSOR (EC 3.2.1.52) (N-ACETYL-BETA- GLUCOSAMINIDASE) (BETA-N-ACETYLHEXOSAMINIDASE) (HEXOSAMINIDASE B). |
| [M200000599](http://omad.operon.com/mouseV3/transcript.php?what=M200000599) | [Pps](http://www.informatics.jax.org/searches/accession_report.cgi?id=MGI%3A1194899) | [NM_008916](http://srs.sanger.ac.uk/srsbin/cgi-bin/wgetz?-e+%5BREFSEQ-ID:NM_008916%5D) | [AK054436](http://www.ebi.ac.uk/cgi-bin/emblfetch?AK054436) | PUTATIVE PHOSPHATASE; PI-5-PHOSPHATASE RELATED; PUTATIVE PI-5-PHOSPHATASE. [ |
| [M200014192](http://omad.operon.com/mouseV3/transcript.php?what=M200014192) | -- | [NM_053193](http://srs.sanger.ac.uk/srsbin/cgi-bin/wgetz?-e+%5BREFSEQ-ID:NM_053193%5D) | [AF322193](http://www.ebi.ac.uk/cgi-bin/emblfetch?AF322193) | CLEAVAGE AND POLYADENYLATION SPECIFICITY FACTOR, 160 KDA SUBUNIT (CPSF 160 KDA SUBUNIT). |
| [M200004343](http://omad.operon.com/mouseV3/transcript.php?what=M200004343) | [4833412N02Rik](http://www.informatics.jax.org/searches/accession_report.cgi?id=MGI%3A1921860) | [NM_029020](http://srs.sanger.ac.uk/srsbin/cgi-bin/wgetz?-e+%5BREFSEQ-ID:NM_029020%5D) | [AK030624](http://www.ebi.ac.uk/cgi-bin/emblfetch?AK030624) | -- |
| [M200002381](http://omad.operon.com/mouseV3/transcript.php?what=M200002381) | [Fanca](http://www.informatics.jax.org/searches/accession_report.cgi?id=MGI%3A1341823) | [NM_016925](http://srs.sanger.ac.uk/srsbin/cgi-bin/wgetz?-e+%5BREFSEQ-ID:NM_016925%5D) | [AF178934](http://www.ebi.ac.uk/cgi-bin/emblfetch?AF178934) | FANCONI ANEMIA, COMPLEMENTATION GROUP A. |
| **200 Gy** |  |  |  |  |
| [M200013484](http://omad.operon.com/mouseV3/transcript.php?what=M200013484) | [9030617O03Rik](http://www.informatics.jax.org/searches/accession_report.cgi?id=MGI%3A2444813) | [NM_145448](http://srs.sanger.ac.uk/srsbin/cgi-bin/wgetz?-e+%5BREFSEQ-ID:NM_145448%5D) | [BC021385](http://www.ebi.ac.uk/cgi-bin/emblfetch?BC021385) | -- |
| [M200000800](http://omad.operon.com/mouseV3/transcript.php?what=M200000800) | [Ccng1](http://www.informatics.jax.org/searches/accession_report.cgi?id=MGI%3A102890) | [NM_009831](http://srs.sanger.ac.uk/srsbin/cgi-bin/wgetz?-e+%5BREFSEQ-ID:NM_009831%5D) | [AB005559](http://www.ebi.ac.uk/cgi-bin/emblfetch?AB005559) | CYCLIN G1 (CYCLIN G). |
| [M200016031](http://omad.operon.com/mouseV3/transcript.php?what=M200016031) | [Polk](http://www.informatics.jax.org/searches/accession_report.cgi?id=MGI%3A1349767) | [NM_012048](http://srs.sanger.ac.uk/srsbin/cgi-bin/wgetz?-e+%5BREFSEQ-ID:NM_012048%5D) | [AB040764](http://www.ebi.ac.uk/cgi-bin/emblfetch?AB040764) | POLYMERASE (DNA DIRECTED), KAPPA; DINB HOMOLOG 1 (E. COLI); DNA DAMAGE-INDUCIBLE PROETIN B; DNA DAMAGE-INDUCIBLE PROTEIN B; POLYMERASE (DNA DIRECTED) KAPPA. |
| [M200007794](http://omad.operon.com/mouseV3/transcript.php?what=M200007794) | [Wig1](http://www.informatics.jax.org/searches/accession_report.cgi?id=MGI%3A1195270) | [NM_009517](http://srs.sanger.ac.uk/srsbin/cgi-bin/wgetz?-e+%5BREFSEQ-ID:NM_009517%5D) | [AF012923](http://www.ebi.ac.uk/cgi-bin/emblfetch?AF012923) | WILD-TYPE P53-INDUCED GENE 1. |
| [M300006854](http://omad.operon.com/mouseV3/transcript.php?what=M300006854) | [Sec8](http://www.informatics.jax.org/searches/accession_report.cgi?id=MGI%3A1096376) | [NM_009148](http://srs.sanger.ac.uk/srsbin/cgi-bin/wgetz?-e+%5BREFSEQ-ID:NM_009148%5D) | [BC034644](http://www.ebi.ac.uk/cgi-bin/emblfetch?BC034644) | EXOCYST COMPLEX COMPONENT SEC8. [Source:SWISSPROT;Acc:O35382] |
| [M200006137](http://omad.operon.com/mouseV3/transcript.php?what=M200006137) | [Stinp](http://www.informatics.jax.org/searches/accession_report.cgi?id=MGI%3A1926609) | [NM_021897](http://srs.sanger.ac.uk/srsbin/cgi-bin/wgetz?-e+%5BREFSEQ-ID:NM_021897%5D) | [AY034612](http://www.ebi.ac.uk/cgi-bin/emblfetch?AY034612) | STRESS INDUCED PROTEIN; THYMUS EXPRESSED ACIDIC PROTEIN. |
| [M200007477](http://omad.operon.com/mouseV3/transcript.php?what=M200007477) | [2310047O13Rik](http://www.informatics.jax.org/searches/accession_report.cgi?id=MGI%3A1914210) | [NM_024185](http://srs.sanger.ac.uk/srsbin/cgi-bin/wgetz?-e+%5BREFSEQ-ID:NM_024185%5D) | [BC027202](http://www.ebi.ac.uk/cgi-bin/emblfetch?BC027202) | -- |
| [M300020474](http://omad.operon.com/mouseV3/transcript.php?what=M300020474) | -- | -- | -- | -- |
| [M200003982](http://omad.operon.com/mouseV3/transcript.php?what=M200003982) | [Golga5](http://www.informatics.jax.org/searches/accession_report.cgi?id=MGI%3A1351475) | [NM_013747](http://srs.sanger.ac.uk/srsbin/cgi-bin/wgetz?-e+%5BREFSEQ-ID:NM_013747%5D) | [AF026274](http://www.ebi.ac.uk/cgi-bin/emblfetch?AF026274) | GOLGI AUTOANTIGEN, GOLGIN SUBFAMILY A, 5. |
| [M300020472](http://omad.operon.com/mouseV3/transcript.php?what=M300020472) | -- | -- | -- | -- |
| [M200004045](http://omad.operon.com/mouseV3/transcript.php?what=M200004045) | [AI504353](http://www.informatics.jax.org/searches/accession_report.cgi?id=MGI%3A2141989) | [NM_153419](http://srs.sanger.ac.uk/srsbin/cgi-bin/wgetz?-e+%5BREFSEQ-ID:NM_153419%5D) | [BC008121](http://www.ebi.ac.uk/cgi-bin/emblfetch?BC008121) | GLUTAMATE RICH WD REPEAT PROTEIN GRWD.] |
| [M200002527](http://omad.operon.com/mouseV3/transcript.php?what=M200002527) | [Cnbp](http://www.informatics.jax.org/searches/accession_report.cgi?id=MGI%3A88431) | [NM_013493](http://srs.sanger.ac.uk/srsbin/cgi-bin/wgetz?-e+%5BREFSEQ-ID:NM_013493%5D) | [U20326](http://www.ebi.ac.uk/cgi-bin/emblfetch?U20326) | CELLULAR NUCLEIC ACID BINDING PROTEIN (CNBP).] |
| [M200014192](http://omad.operon.com/mouseV3/transcript.php?what=M200014192) | -- | [NM_053193](http://srs.sanger.ac.uk/srsbin/cgi-bin/wgetz?-e+%5BREFSEQ-ID:NM_053193%5D) | [AF322193](http://www.ebi.ac.uk/cgi-bin/emblfetch?AF322193) | CLEAVAGE AND POLYADENYLATION SPECIFICITY FACTOR, 160 KDA SUBUNIT (CPSF 160 KDA SUBUNIT).] |
| [M300000277](http://omad.operon.com/mouseV3/transcript.php?what=M300000277) | [2310004L02Rik](http://www.informatics.jax.org/searches/accession_report.cgi?id=MGI%3A1913599) | [NM_025504](http://srs.sanger.ac.uk/srsbin/cgi-bin/wgetz?-e+%5BREFSEQ-ID:NM_025504%5D) | [AK009150](http://www.ebi.ac.uk/cgi-bin/emblfetch?AK009150) | -- |
| [M200012890](http://omad.operon.com/mouseV3/transcript.php?what=M200012890) | [Smarca4](http://www.informatics.jax.org/searches/accession_report.cgi?id=MGI%3A88192) | -- | [BC026672](http://www.ebi.ac.uk/cgi-bin/emblfetch?BC026672) | -- |
| [M200005377](http://omad.operon.com/mouseV3/transcript.php?what=M200005377) | [Itpr3](http://www.informatics.jax.org/searches/accession_report.cgi?id=MGI%3A96624) | [NM_080553](http://srs.sanger.ac.uk/srsbin/cgi-bin/wgetz?-e+%5BREFSEQ-ID:NM_080553%5D) | [Z71174](http://www.ebi.ac.uk/cgi-bin/emblfetch?Z71174) | INOSITOL 1,4,5-TRISPHOSPHATE RECEPTOR TYPE 3 (TYPE 3 INOSITOL 1,4,5- TRISPHOSPHATE RECEPTOR) (TYPE 3 INSP3 RECEPTOR) (IP3 RECEPTOR ISOFORM 3) (INSP3R3) (FRAGMENT). [ |
| [M200002473](http://omad.operon.com/mouseV3/transcript.php?what=M200002473) | [Acas2l](http://www.informatics.jax.org/searches/accession_report.cgi?id=MGI%3A1915988) | [NM_080575](http://srs.sanger.ac.uk/srsbin/cgi-bin/wgetz?-e+%5BREFSEQ-ID:NM_080575%5D) | [AK088244](http://www.ebi.ac.uk/cgi-bin/emblfetch?AK088244) | ACETYL-COA SYNTHETASE 2-LIKE; ACETYL-COENZYME A SYNTHETASE 2. |
| [M300011684](http://omad.operon.com/mouseV3/transcript.php?what=M300011684) | [Pold1](http://www.informatics.jax.org/searches/accession_report.cgi?id=MGI%3A97741) | [NM_011131](http://srs.sanger.ac.uk/srsbin/cgi-bin/wgetz?-e+%5BREFSEQ-ID:NM_011131%5D) | [AF024570](http://www.ebi.ac.uk/cgi-bin/emblfetch?AF024570) | DNA POLYMERASE DELTA CATALYTIC SUBUNIT (EC 2.7.7.7). |
| [M300009152](http://omad.operon.com/mouseV3/transcript.php?what=M300009152) | [Tpst1](http://www.informatics.jax.org/searches/accession_report.cgi?id=MGI%3A1298231) | [NM_013837](http://srs.sanger.ac.uk/srsbin/cgi-bin/wgetz?-e+%5BREFSEQ-ID:NM_013837%5D) | [AF038008](http://www.ebi.ac.uk/cgi-bin/emblfetch?AF038008) | PROTEIN-TYROSINE SULFOTRANSFERASE 1 (EC 2.8.2.20) (TYROSYLPROTEIN SULFOTRANSFERASE-1) (TPST-1). |
| [M200014327](http://omad.operon.com/mouseV3/transcript.php?what=M200014327) | [Bcar3](http://www.informatics.jax.org/searches/accession_report.cgi?id=MGI%3A1352501) | [NM_013867](http://srs.sanger.ac.uk/srsbin/cgi-bin/wgetz?-e+%5BREFSEQ-ID:NM_013867%5D) | [BC023930](http://www.ebi.ac.uk/cgi-bin/emblfetch?BC023930) | BREAST CANCER ANTI-ESTROGEN RESISTANCE3 |
| [M300013112](http://omad.operon.com/mouseV3/transcript.php?what=M300013112) | -- | -- | [J00595](http://www.ebi.ac.uk/cgi-bin/emblfetch?J00595) | IG LAMBDA-2 CHAIN C REGION. |
| [M200006566](http://omad.operon.com/mouseV3/transcript.php?what=M200006566) | [Gga2](http://www.informatics.jax.org/searches/accession_report.cgi?id=MGI%3A1921355) | -- | [AK004632](http://www.ebi.ac.uk/cgi-bin/emblfetch?AK004632) | -- |
| [M300007254](http://omad.operon.com/mouseV3/transcript.php?what=M300007254) | -- | [NM_172900](http://srs.sanger.ac.uk/srsbin/cgi-bin/wgetz?-e+%5BREFSEQ-ID:NM_172900%5D) | -- | -- |
| [M200009317](http://omad.operon.com/mouseV3/transcript.php?what=M200009317) | [Scd1](http://www.informatics.jax.org/searches/accession_report.cgi?id=MGI%3A98239) | [NM_009127](http://srs.sanger.ac.uk/srsbin/cgi-bin/wgetz?-e+%5BREFSEQ-ID:NM_009127%5D) | [BC007474](http://www.ebi.ac.uk/cgi-bin/emblfetch?BC007474) | ACYL-COA DESATURASE 1 (EC 1.14.19.1) (STEAROYL-COA DESATURASE 1) (FATTY ACID DESATURASE 1) (DELTA(9)-DESATURASE 1). |
| [M200001144](http://omad.operon.com/mouseV3/transcript.php?what=M200001144) | [Cd79b](http://www.informatics.jax.org/searches/accession_report.cgi?id=MGI%3A96431) | [NM_008339](http://srs.sanger.ac.uk/srsbin/cgi-bin/wgetz?-e+%5BREFSEQ-ID:NM_008339%5D) | [AF002279](http://www.ebi.ac.uk/cgi-bin/emblfetch?AF002279) | B-CELL ANTIGEN RECEPTOR COMPLEX ASSOCIATED PROTEIN BETA-CHAIN PRECURSOR (B-CELL-SPECIFIC GLYCOPROTEIN B29) (IMMUNOGLOBULIN- ASSOCIATED B29 PROTEIN) (IG-BETA) (CD79B). |
| [M200004687](http://omad.operon.com/mouseV3/transcript.php?what=M200004687) | [Dda3-pending](http://www.informatics.jax.org/searches/accession_report.cgi?id=MGI%3A1913099) | [NM_019976](http://srs.sanger.ac.uk/srsbin/cgi-bin/wgetz?-e+%5BREFSEQ-ID:NM_019976%5D) | [AK041835](http://www.ebi.ac.uk/cgi-bin/emblfetch?AK041835) | DIFFERENTIAL DISPLAY AND ACTIVATED BY P53; P53-REGULATED DDA3. |
| [M300020088](http://omad.operon.com/mouseV3/transcript.php?what=M300020088) | -- | -- | -- | -- |
| [M300004256](http://omad.operon.com/mouseV3/transcript.php?what=M300004256) | [Fth](http://www.informatics.jax.org/searches/accession_report.cgi?id=MGI%3A95588) | [NM_010239](http://srs.sanger.ac.uk/srsbin/cgi-bin/wgetz?-e+%5BREFSEQ-ID:NM_010239%5D) | [M24509](http://www.ebi.ac.uk/cgi-bin/emblfetch?M24509) | FERRITIN HEAVY CHAIN (FERRITIN H SUBUNIT). |
| [M300014099](http://omad.operon.com/mouseV3/transcript.php?what=M300014099) | [Actl](http://www.informatics.jax.org/searches/accession_report.cgi?id=MGI%3A109429) | [NM_013798](http://srs.sanger.ac.uk/srsbin/cgi-bin/wgetz?-e+%5BREFSEQ-ID:NM_013798%5D) | [AF195094](http://www.ebi.ac.uk/cgi-bin/emblfetch?AF195094) | ACTIN-LIKE. |
| [M300020371](http://omad.operon.com/mouseV3/transcript.php?what=M300020371) | -- | -- | -- | -- |
| [M200006851](http://omad.operon.com/mouseV3/transcript.php?what=M200006851) | -- | [NM_026467](http://srs.sanger.ac.uk/srsbin/cgi-bin/wgetz?-e+%5BREFSEQ-ID:NM_026467%5D) | -- | RIBOSOMAL PROTEIN S27-LIKE. |
| [M300015889](http://omad.operon.com/mouseV3/transcript.php?what=M300015889) | -- | -- | -- | -- |
| [M300019801](http://omad.operon.com/mouseV3/transcript.php?what=M300019801) | -- | -- | -- | -- |
| [M300018553](http://omad.operon.com/mouseV3/transcript.php?what=M300018553) | -- | -- | -- | -- |
| [M300021441](http://omad.operon.com/mouseV3/transcript.php?what=M300021441) | -- | -- | -- | -- |
| [M300015305](http://omad.operon.com/mouseV3/transcript.php?what=M300015305) | -- | -- | -- | -- |
| [M300019335](http://omad.operon.com/mouseV3/transcript.php?what=M300019335) | [Gapd](http://www.informatics.jax.org/searches/accession_report.cgi?id=MGI%3A95640) | [NM_008084](http://srs.sanger.ac.uk/srsbin/cgi-bin/wgetz?-e+%5BREFSEQ-ID:NM_008084%5D) | [AK002273](http://www.ebi.ac.uk/cgi-bin/emblfetch?AK002273) | GLYCERALDEHYDE 3-PHOSPHATE DEHYDROGENASE (EC 1.2.1.12) (GAPDH). |
| [M300020777](http://omad.operon.com/mouseV3/transcript.php?what=M300020777) | -- | -- | -- | -- |
| [M200003258](http://omad.operon.com/mouseV3/transcript.php?what=M200003258) | [Cox8a](http://www.informatics.jax.org/searches/accession_report.cgi?id=MGI%3A105959) | [NM_007750](http://srs.sanger.ac.uk/srsbin/cgi-bin/wgetz?-e+%5BREFSEQ-ID:NM_007750%5D) | [U37721](http://www.ebi.ac.uk/cgi-bin/emblfetch?U37721) | CYTOCHROME C OXIDASE POLYPEPTIDE VIII-LIVER, MITOCHONDRIAL PRECURSOR (EC 1.9.3.1). |
| [M300014515](http://omad.operon.com/mouseV3/transcript.php?what=M300014515) | -- | -- | -- | -- |
| [M300018314](http://omad.operon.com/mouseV3/transcript.php?what=M300018314) | -- | -- | -- | -- |
| [M200001083](http://omad.operon.com/mouseV3/transcript.php?what=M200001083) | [Hspa9a](http://www.informatics.jax.org/searches/accession_report.cgi?id=MGI%3A96245) | [NM_010481](http://srs.sanger.ac.uk/srsbin/cgi-bin/wgetz?-e+%5BREFSEQ-ID:NM_010481%5D) | [AK002634](http://www.ebi.ac.uk/cgi-bin/emblfetch?AK002634) | STRESS-70 PROTEIN, MITOCHONDRIAL PRECURSOR (75 KDA GLUCOSE REGULATED PROTEIN) (GRP 75) (PEPTIDE-BINDING PROTEIN 74) (PBP74) (P66 MOT) (MORTALIN). |
| [M300018559](http://omad.operon.com/mouseV3/transcript.php?what=M300018559) | -- | -- | -- | -- |
| [M300012796](http://omad.operon.com/mouseV3/transcript.php?what=M300012796) | [Hmgn1](http://www.informatics.jax.org/searches/accession_report.cgi?id=MGI%3A96120) | [NM_008251](http://srs.sanger.ac.uk/srsbin/cgi-bin/wgetz?-e+%5BREFSEQ-ID:NM_008251%5D) | [X53476](http://www.ebi.ac.uk/cgi-bin/emblfetch?X53476) | NONHISTONE CHROMOSOMAL PROTEIN HMG-14 (HIGH-MOBILITY GROUP NUCLEOSOME BINDING DOMAIN 1). |
| [M200000777](http://omad.operon.com/mouseV3/transcript.php?what=M200000777) | [G3bp-pending](http://www.informatics.jax.org/searches/accession_report.cgi?id=MGI%3A1351465) | [NM_013716](http://srs.sanger.ac.uk/srsbin/cgi-bin/wgetz?-e+%5BREFSEQ-ID:NM_013716%5D) | [AB001927](http://www.ebi.ac.uk/cgi-bin/emblfetch?AB001927) | RAS-GTPASE-ACTIVATING PROTEIN BINDING PROTEIN 1 (GAP SH3-DOMAIN BINDING PROTEIN 1) (G3BP-1). |
| [M300021668](http://omad.operon.com/mouseV3/transcript.php?what=M300021668) | -- | -- | -- | -- |
| [M300002115](http://omad.operon.com/mouseV3/transcript.php?what=M300002115) | [Xpo1](http://www.informatics.jax.org/searches/accession_report.cgi?id=MGI%3A2144013) | [NM_134014](http://srs.sanger.ac.uk/srsbin/cgi-bin/wgetz?-e+%5BREFSEQ-ID:NM_134014%5D) | [BC025628](http://www.ebi.ac.uk/cgi-bin/emblfetch?BC025628) | EXPORTIN 1, CRM1 HOMOLOG; EXPRESSED SEQUENCE AA420417. |
| [M300017554](http://omad.operon.com/mouseV3/transcript.php?what=M300017554) | [4930415K17Rik](http://www.informatics.jax.org/searches/accession_report.cgi?id=MGI%3A1914643) | [NM_133687](http://srs.sanger.ac.uk/srsbin/cgi-bin/wgetz?-e+%5BREFSEQ-ID:NM_133687%5D) | [BC016207](http://www.ebi.ac.uk/cgi-bin/emblfetch?BC016207) | -- |
| [M300004265](http://omad.operon.com/mouseV3/transcript.php?what=M300004265) | [Ms4a1](http://www.informatics.jax.org/searches/accession_report.cgi?id=MGI%3A88321) | [NM_007641](http://srs.sanger.ac.uk/srsbin/cgi-bin/wgetz?-e+%5BREFSEQ-ID:NM_007641%5D) | [AK017903](http://www.ebi.ac.uk/cgi-bin/emblfetch?AK017903) | B-CELL SURFACE PROTEIN CD20 HOMOLOG (B-CELL DIFFERENTIATION ANTIGEN LY-44). |
| [M200001144](http://omad.operon.com/mouseV3/transcript.php?what=M200001144) | [Cd79b](http://www.informatics.jax.org/searches/accession_report.cgi?id=MGI%3A96431) | [NM_008339](http://srs.sanger.ac.uk/srsbin/cgi-bin/wgetz?-e+%5BREFSEQ-ID:NM_008339%5D) | [AF002279](http://www.ebi.ac.uk/cgi-bin/emblfetch?AF002279) | B-CELL ANTIGEN RECEPTOR COMPLEX ASSOCIATED PROTEIN BETA-CHAIN PRECURSOR (B-CELL-SPECIFIC GLYCOPROTEIN B29) (IMMUNOGLOBULIN- ASSOCIATED B29 PROTEIN) (IG-BETA) (CD79B). |
| **1000 Gy** |  |  |  |  |
| [M200007547](http://omad.operon.com/mouseV3/transcript.php?what=M200007547) | [Phlda3](http://www.informatics.jax.org/searches/accession_report.cgi?id=MGI%3A1351485) | [NM_013750](http://srs.sanger.ac.uk/srsbin/cgi-bin/wgetz?-e+%5BREFSEQ-ID:NM_013750%5D) | [BC023408](http://www.ebi.ac.uk/cgi-bin/emblfetch?BC023408) | PLECKSTRIN HOMOLOGY-LIKE DOMAIN, FAMILY A, MEMBER 3; TDAG/LPL HOMOLOG 1. |
| [M200016031](http://omad.operon.com/mouseV3/transcript.php?what=M200016031) | [Polk](http://www.informatics.jax.org/searches/accession_report.cgi?id=MGI%3A1349767) | [NM_012048](http://srs.sanger.ac.uk/srsbin/cgi-bin/wgetz?-e+%5BREFSEQ-ID:NM_012048%5D) | [AB040764](http://www.ebi.ac.uk/cgi-bin/emblfetch?AB040764) | POLYMERASE (DNA DIRECTED), KAPPA; DINB HOMOLOG 1 (E. COLI); DNA DAMAGE-INDUCIBLE PROETIN B; DNA DAMAGE-INDUCIBLE PROTEIN B; POLYMERASE (DNA DIRECTED) KAPPA. |
| [M200004687](http://omad.operon.com/mouseV3/transcript.php?what=M200004687) | [Dda3-pending](http://www.informatics.jax.org/searches/accession_report.cgi?id=MGI%3A1913099) | [NM_019976](http://srs.sanger.ac.uk/srsbin/cgi-bin/wgetz?-e+%5BREFSEQ-ID:NM_019976%5D) | [AK041835](http://www.ebi.ac.uk/cgi-bin/emblfetch?AK041835) | DIFFERENTIAL DISPLAY AND ACTIVATED BY P53; P53-REGULATED DDA3. |
| [M200007578](http://omad.operon.com/mouseV3/transcript.php?what=M200007578) | [Cdkn1a](http://www.informatics.jax.org/searches/accession_report.cgi?id=MGI%3A104556) | [NM_007669](http://srs.sanger.ac.uk/srsbin/cgi-bin/wgetz?-e+%5BREFSEQ-ID:NM_007669%5D) | [U24173](http://www.ebi.ac.uk/cgi-bin/emblfetch?U24173) | CYCLIN-DEPENDENT KINASE INHIBITOR 1 (P21) (CDK-INTERACTING PROTEIN 1) (MELANOMA DIFFERENTIATION ASSOCIATED PROTEIN). |
| [M200007794](http://omad.operon.com/mouseV3/transcript.php?what=M200007794) | [Wig1](http://www.informatics.jax.org/searches/accession_report.cgi?id=MGI%3A1195270) | [NM_009517](http://srs.sanger.ac.uk/srsbin/cgi-bin/wgetz?-e+%5BREFSEQ-ID:NM_009517%5D) | [AF012923](http://www.ebi.ac.uk/cgi-bin/emblfetch?AF012923) | WILD-TYPE P53-INDUCED GENE 1. |
| [M200015712](http://omad.operon.com/mouseV3/transcript.php?what=M200015712) | [3300002K07Rik](http://www.informatics.jax.org/searches/accession_report.cgi?id=MGI%3A1917675) | [NM_152809](http://srs.sanger.ac.uk/srsbin/cgi-bin/wgetz?-e+%5BREFSEQ-ID:NM_152809%5D) | [BC033601](http://www.ebi.ac.uk/cgi-bin/emblfetch?BC033601) | -- |
| [M300000277](http://omad.operon.com/mouseV3/transcript.php?what=M300000277) | [2310004L02Rik](http://www.informatics.jax.org/searches/accession_report.cgi?id=MGI%3A1913599) | [NM_025504](http://srs.sanger.ac.uk/srsbin/cgi-bin/wgetz?-e+%5BREFSEQ-ID:NM_025504%5D) | [AK009150](http://www.ebi.ac.uk/cgi-bin/emblfetch?AK009150) | -- |
| [M300003012](http://omad.operon.com/mouseV3/transcript.php?what=M300003012) | -- | -- | -- | -- |
| [M200009576](http://omad.operon.com/mouseV3/transcript.php?what=M200009576) | [Recc1](http://www.informatics.jax.org/searches/accession_report.cgi?id=MGI%3A97891) | [NM_011258](http://srs.sanger.ac.uk/srsbin/cgi-bin/wgetz?-e+%5BREFSEQ-ID:NM_011258%5D) | [U15037](http://www.ebi.ac.uk/cgi-bin/emblfetch?U15037) | ACTIVATOR 1 140 KDA SUBUNIT (REPLICATION FACTOR C LARGE SUBUNIT) (A1 140 KDA SUBUNIT) (RF-C 140 KDA SUBUNIT) (ACTIVATOR 1 LARGE SUBUNIT) (A1-P145) (DIFFERENTIATION SPECIFIC ELEMENT BINDING PROTEIN) (ISRE-BINDING PROTEIN). |
| [M300011684](http://omad.operon.com/mouseV3/transcript.php?what=M300011684) | [Pold1](http://www.informatics.jax.org/searches/accession_report.cgi?id=MGI%3A97741) | [NM_011131](http://srs.sanger.ac.uk/srsbin/cgi-bin/wgetz?-e+%5BREFSEQ-ID:NM_011131%5D) | [AF024570](http://www.ebi.ac.uk/cgi-bin/emblfetch?AF024570) | DNA POLYMERASE DELTA CATALYTIC SUBUNIT (EC 2.7.7.7). |
| [M300010073](http://omad.operon.com/mouseV3/transcript.php?what=M300010073) | -- | -- | -- | -- |
| [M200004560](http://omad.operon.com/mouseV3/transcript.php?what=M200004560) | -- | [NM_026942](http://srs.sanger.ac.uk/srsbin/cgi-bin/wgetz?-e+%5BREFSEQ-ID:NM_026942%5D) | -- | -- |
| [M200005905](http://omad.operon.com/mouseV3/transcript.php?what=M200005905) | -- | -- | [BC022623](http://www.ebi.ac.uk/cgi-bin/emblfetch?BC022623) | -- |
| [M200002473](http://omad.operon.com/mouseV3/transcript.php?what=M200002473) | [Acas2l](http://www.informatics.jax.org/searches/accession_report.cgi?id=MGI%3A1915988) | [NM_080575](http://srs.sanger.ac.uk/srsbin/cgi-bin/wgetz?-e+%5BREFSEQ-ID:NM_080575%5D) | [AK088244](http://www.ebi.ac.uk/cgi-bin/emblfetch?AK088244) | ACETYL-COA SYNTHETASE 2-LIKE; ACETYL-COENZYME A SYNTHETASE 2. |
| [M200006174](http://omad.operon.com/mouseV3/transcript.php?what=M200006174) | [0610039P13Rik](http://www.informatics.jax.org/searches/accession_report.cgi?id=MGI%3A1921346) | [NM_028752](http://srs.sanger.ac.uk/srsbin/cgi-bin/wgetz?-e+%5BREFSEQ-ID:NM_028752%5D) | [BC021548](http://www.ebi.ac.uk/cgi-bin/emblfetch?BC021548) | -- |
| [M200014932](http://omad.operon.com/mouseV3/transcript.php?what=M200014932) | [Swap70](http://www.informatics.jax.org/searches/accession_report.cgi?id=MGI%3A1298390) | [NM_009302](http://srs.sanger.ac.uk/srsbin/cgi-bin/wgetz?-e+%5BREFSEQ-ID:NM_009302%5D) | [AF053974](http://www.ebi.ac.uk/cgi-bin/emblfetch?AF053974) | SWAP COMPLEX PROTEIN; SWAP COMPLEX PROTEIN, 70 KDA. |
| [M200006566](http://omad.operon.com/mouseV3/transcript.php?what=M200006566) | [Gga2](http://www.informatics.jax.org/searches/accession_report.cgi?id=MGI%3A1921355) | -- | [AK004632](http://www.ebi.ac.uk/cgi-bin/emblfetch?AK004632) | -- |
| [M200000662](http://omad.operon.com/mouseV3/transcript.php?what=M200000662) | [Dtx1](http://www.informatics.jax.org/searches/accession_report.cgi?id=MGI%3A1352744) | [NM_008052](http://srs.sanger.ac.uk/srsbin/cgi-bin/wgetz?-e+%5BREFSEQ-ID:NM_008052%5D) | [AB015422](http://www.ebi.ac.uk/cgi-bin/emblfetch?AB015422) | DELTEX 1 HOMOLOG (DROSOPHILA); FRACTIONATED X-IRRADIATION INDUCED TRANSCRIPT 1. |
| [M300007360](http://omad.operon.com/mouseV3/transcript.php?what=M300007360) | -- | -- | -- | -- |
| [M300013112](http://omad.operon.com/mouseV3/transcript.php?what=M300013112) | -- | -- | [J00595](http://www.ebi.ac.uk/cgi-bin/emblfetch?J00595) | IG LAMBDA-2 CHAIN C REGION. |
| [M300004265](http://omad.operon.com/mouseV3/transcript.php?what=M300004265) | [Ms4a1](http://www.informatics.jax.org/searches/accession_report.cgi?id=MGI%3A88321) | [NM_007641](http://srs.sanger.ac.uk/srsbin/cgi-bin/wgetz?-e+%5BREFSEQ-ID:NM_007641%5D) | [AK017903](http://www.ebi.ac.uk/cgi-bin/emblfetch?AK017903) | B-CELL SURFACE PROTEIN CD20 HOMOLOG (B-CELL DIFFERENTIATION ANTIGEN LY-44). |
| [M300007254](http://omad.operon.com/mouseV3/transcript.php?what=M300007254) | -- | [NM_172900](http://srs.sanger.ac.uk/srsbin/cgi-bin/wgetz?-e+%5BREFSEQ-ID:NM_172900%5D) | -- | -- |
| [M300000491](http://omad.operon.com/mouseV3/transcript.php?what=M300000491) | -- | -- | [AF287275](http://www.ebi.ac.uk/cgi-bin/emblfetch?AF287275) | IG LAMBDA-1 CHAIN V REGION PRECURSOR. |
| [M200009317](http://omad.operon.com/mouseV3/transcript.php?what=M200009317) | [Scd1](http://www.informatics.jax.org/searches/accession_report.cgi?id=MGI%3A98239) | [NM_009127](http://srs.sanger.ac.uk/srsbin/cgi-bin/wgetz?-e+%5BREFSEQ-ID:NM_009127%5D) | [BC007474](http://www.ebi.ac.uk/cgi-bin/emblfetch?BC007474) | ACYL-COA DESATURASE 1 (EC 1.14.19.1) (STEAROYL-COA DESATURASE 1) (FATTY ACID DESATURASE 1) (DELTA(9)-DESATURASE 1). |
| [M200001144](http://omad.operon.com/mouseV3/transcript.php?what=M200001144) | [Cd79b](http://www.informatics.jax.org/searches/accession_report.cgi?id=MGI%3A96431) | [NM_008339](http://srs.sanger.ac.uk/srsbin/cgi-bin/wgetz?-e+%5BREFSEQ-ID:NM_008339%5D) | [AF002279](http://www.ebi.ac.uk/cgi-bin/emblfetch?AF002279) | B-CELL ANTIGEN RECEPTOR COMPLEX ASSOCIATED PROTEIN BETA-CHAIN PRECURSOR (B-CELL-SPECIFIC GLYCOPROTEIN B29) (IMMUNOGLOBULIN- ASSOCIATED B29 PROTEIN) (IG-BETA) (CD79B). |

| **FEMALES** |  |  |  |  |
| --- | --- | --- | --- | --- |
| **50 Gy** |  |  |  |  |
| [M300002291](http://omad.operon.com/mouseV3/transcript.php?what=M300002291) | -- | -- | -- | -- |
| [M200004687](http://omad.operon.com/mouseV3/transcript.php?what=M200004687) | [Dda3-pending](http://www.informatics.jax.org/searches/accession_report.cgi?id=MGI%3A1913099) | [NM_019976](http://srs.sanger.ac.uk/srsbin/cgi-bin/wgetz?-e+%5BREFSEQ-ID:NM_019976%5D) | [AK041835](http://www.ebi.ac.uk/cgi-bin/emblfetch?AK041835) | DIFFERENTIAL DISPLAY AND ACTIVATED BY P53; P53-REGULATED DDA3. |
| [M200000800](http://omad.operon.com/mouseV3/transcript.php?what=M200000800) | [Ccng1](http://www.informatics.jax.org/searches/accession_report.cgi?id=MGI%3A102890) | [NM_009831](http://srs.sanger.ac.uk/srsbin/cgi-bin/wgetz?-e+%5BREFSEQ-ID:NM_009831%5D) | [AB005559](http://www.ebi.ac.uk/cgi-bin/emblfetch?AB005559) | CYCLIN G1 (CYCLIN G) |
| [M300016629](http://omad.operon.com/mouseV3/transcript.php?what=M300016629) | -- | -- | -- | -- |
| [M300020491](http://omad.operon.com/mouseV3/transcript.php?what=M300020491) | -- | -- | [U38498](http://www.ebi.ac.uk/cgi-bin/emblfetch?U38498) | GUANINE NUCLEOTIDE-BINDING PROTEIN G(I)/G(S)/G(O) GAMMA-5 SUBUNIT. |
| [M300015969](http://omad.operon.com/mouseV3/transcript.php?what=M300015969) | -- | -- | -- | -- |
| [M200006491](http://omad.operon.com/mouseV3/transcript.php?what=M200006491) | [Pgls](http://www.informatics.jax.org/searches/accession_report.cgi?id=MGI%3A1913421) | [NM_025396](http://srs.sanger.ac.uk/srsbin/cgi-bin/wgetz?-e+%5BREFSEQ-ID:NM_025396%5D) | [BC006594](http://www.ebi.ac.uk/cgi-bin/emblfetch?BC006594) | 6-PHOSPHOGLUCONOLACTONASE. |
| [M300010063](http://omad.operon.com/mouseV3/transcript.php?what=M300010063) | -- | -- | -- | -- |
| [M300016018](http://omad.operon.com/mouseV3/transcript.php?what=M300016018) | -- | [NM_023133](http://srs.sanger.ac.uk/srsbin/cgi-bin/wgetz?-e+%5BREFSEQ-ID:NM_023133%5D) | -- | RIBOSOMAL PROTEIN S19. |
| [M200002378](http://omad.operon.com/mouseV3/transcript.php?what=M200002378) | [S100a13](http://www.informatics.jax.org/searches/accession_report.cgi?id=MGI%3A109581) | [NM_009113](http://srs.sanger.ac.uk/srsbin/cgi-bin/wgetz?-e+%5BREFSEQ-ID:NM_009113%5D) | [BC005687](http://www.ebi.ac.uk/cgi-bin/emblfetch?BC005687) | S100 CALCIUM-BINDING PROTEIN A13. |
| [M300019659](http://omad.operon.com/mouseV3/transcript.php?what=M300019659) | -- | -- | -- | -- |
| [M300019012](http://omad.operon.com/mouseV3/transcript.php?what=M300019012) | -- | -- | -- | -- |
| [M300009287](http://omad.operon.com/mouseV3/transcript.php?what=M300009287) | -- | -- | -- | -- |
| [M300002125](http://omad.operon.com/mouseV3/transcript.php?what=M300002125) | -- | -- | -- | -- |
| [M300008077](http://omad.operon.com/mouseV3/transcript.php?what=M300008077) | [Ei24](http://www.informatics.jax.org/searches/accession_report.cgi?id=MGI%3A108090) | [NM_007915](http://srs.sanger.ac.uk/srsbin/cgi-bin/wgetz?-e+%5BREFSEQ-ID:NM_007915%5D) | [U41751](http://www.ebi.ac.uk/cgi-bin/emblfetch?U41751) | ETOPOSIDE-INDUCED PROTEIN 2.4. |
| [M200006774](http://omad.operon.com/mouseV3/transcript.php?what=M200006774) | [2400001E08Rik](http://www.informatics.jax.org/searches/accession_report.cgi?id=MGI%3A1913758) | [NM_025605](http://srs.sanger.ac.uk/srsbin/cgi-bin/wgetz?-e+%5BREFSEQ-ID:NM_025605%5D) | [BC020142](http://www.ebi.ac.uk/cgi-bin/emblfetch?BC020142) | -- |
| [M300008474](http://omad.operon.com/mouseV3/transcript.php?what=M300008474) | [D10Jhu81e](http://www.informatics.jax.org/searches/accession_report.cgi?id=MGI%3A1351861) | [NM_138601](http://srs.sanger.ac.uk/srsbin/cgi-bin/wgetz?-e+%5BREFSEQ-ID:NM_138601%5D) | [AB041855](http://www.ebi.ac.uk/cgi-bin/emblfetch?AB041855) | -- |
| [M200000096](http://omad.operon.com/mouseV3/transcript.php?what=M200000096) | [B3Gat3](http://www.informatics.jax.org/searches/accession_report.cgi?id=MGI%3A1919977) | [NM_024256](http://srs.sanger.ac.uk/srsbin/cgi-bin/wgetz?-e+%5BREFSEQ-ID:NM_024256%5D) | [BC002103](http://www.ebi.ac.uk/cgi-bin/emblfetch?BC002103) | GALACTOSYLGALACTOSYLXYLOSYLPROTEIN 3-BETA-GLUCURONOSYLTRANSFERASE 3 (EC 2.4.1.135) (BETA-1,3-GLUCURONYLTRANSFERASE 3) (GLUCURONOSYLTRANSFERASE-I) (GLCAT-I) (UDP-GLCUA:GAL BETA-1,3-GAL-R GLUCURONYLTRANSFERASE) (GLCUAT-I). |
| [M300000948](http://omad.operon.com/mouseV3/transcript.php?what=M300000948) | -- | -- | [AA277150](http://www.ebi.ac.uk/cgi-bin/emblfetch?AA277150) | CLATHRIN COAT ASSEMBLY PROTEIN AP17 (CLATHRIN COAT ASSOCIATED PROTEIN AP17) (PLASMA MEMBRANE ADAPTOR AP-2 17 KDA PROTEIN) (HA2 17 KDA SUBUNIT) (CLATHRIN ASSEMBLY PROTEIN 2 SMALL CHAIN). |
| [M300001725](http://omad.operon.com/mouseV3/transcript.php?what=M300001725) | -- | [NM_175015](http://srs.sanger.ac.uk/srsbin/cgi-bin/wgetz?-e+%5BREFSEQ-ID:NM_175015%5D) | [AA275923](http://www.ebi.ac.uk/cgi-bin/emblfetch?AA275923) | ATP SYNTHASE LIPID-BINDING PROTEIN, MITOCHONDRIAL PRECURSOR (EC 3.6.3.14) (ATP SYNTHASE PROTEOLIPID P3) (ATPASE PROTEIN 9) (ATPASE SUBUNIT C). |
| [M300006374](http://omad.operon.com/mouseV3/transcript.php?what=M300006374) | [Psmc2](http://www.informatics.jax.org/searches/accession_report.cgi?id=MGI%3A109555) | -- | [BC005462](http://www.ebi.ac.uk/cgi-bin/emblfetch?BC005462) | 26S PROTEASE REGULATORY SUBUNIT 7 (MSS1 PROTEIN). |
| [M300005124](http://omad.operon.com/mouseV3/transcript.php?what=M300005124) | [5730454B08Rik](http://www.informatics.jax.org/searches/accession_report.cgi?id=MGI%3A1917829) | [NM_144530](http://srs.sanger.ac.uk/srsbin/cgi-bin/wgetz?-e+%5BREFSEQ-ID:NM_144530%5D) | [BC005786](http://www.ebi.ac.uk/cgi-bin/emblfetch?BC005786) | -- |
| [M200000777](http://omad.operon.com/mouseV3/transcript.php?what=M200000777) | [G3bp-pending](http://www.informatics.jax.org/searches/accession_report.cgi?id=MGI%3A1351465) | [NM_013716](http://srs.sanger.ac.uk/srsbin/cgi-bin/wgetz?-e+%5BREFSEQ-ID:NM_013716%5D) | [AB001927](http://www.ebi.ac.uk/cgi-bin/emblfetch?AB001927) | RAS-GTPASE-ACTIVATING PROTEIN BINDING PROTEIN 1 (GAP SH3-DOMAIN BINDING PROTEIN 1) (G3BP-1). |
| [M200003749](http://omad.operon.com/mouseV3/transcript.php?what=M200003749) | -- | -- | -- | -- |
| [M300018559](http://omad.operon.com/mouseV3/transcript.php?what=M300018559) | -- | -- | -- | -- |
| **200 Gy** |  |  |  |  |
| [M200004687](http://omad.operon.com/mouseV3/transcript.php?what=M200004687) | [Dda3-pending](http://www.informatics.jax.org/searches/accession_report.cgi?id=MGI%3A1913099) | [NM_019976](http://srs.sanger.ac.uk/srsbin/cgi-bin/wgetz?-e+%5BREFSEQ-ID:NM_019976%5D) | [AK041835](http://www.ebi.ac.uk/cgi-bin/emblfetch?AK041835) | DIFFERENTIAL DISPLAY AND ACTIVATED BY P53; P53-REGULATED DDA3. |
| [M300020088](http://omad.operon.com/mouseV3/transcript.php?what=M300020088) | -- | -- | -- | -- |
| [M300004256](http://omad.operon.com/mouseV3/transcript.php?what=M300004256) | [Fth](http://www.informatics.jax.org/searches/accession_report.cgi?id=MGI%3A95588) | [NM_010239](http://srs.sanger.ac.uk/srsbin/cgi-bin/wgetz?-e+%5BREFSEQ-ID:NM_010239%5D) | [M24509](http://www.ebi.ac.uk/cgi-bin/emblfetch?M24509) | FERRITIN HEAVY CHAIN (FERRITIN H SUBUNIT). |
| [M300014099](http://omad.operon.com/mouseV3/transcript.php?what=M300014099) | [Actl](http://www.informatics.jax.org/searches/accession_report.cgi?id=MGI%3A109429) | [NM_013798](http://srs.sanger.ac.uk/srsbin/cgi-bin/wgetz?-e+%5BREFSEQ-ID:NM_013798%5D) | [AF195094](http://www.ebi.ac.uk/cgi-bin/emblfetch?AF195094) | ACTIN-LIKE. |
| [M300020371](http://omad.operon.com/mouseV3/transcript.php?what=M300020371) | -- | -- | -- | -- |
| [M200006851](http://omad.operon.com/mouseV3/transcript.php?what=M200006851) | -- | [NM_026467](http://srs.sanger.ac.uk/srsbin/cgi-bin/wgetz?-e+%5BREFSEQ-ID:NM_026467%5D) | -- | RIBOSOMAL PROTEIN S27-LIKE. |
| [M300015889](http://omad.operon.com/mouseV3/transcript.php?what=M300015889) | -- | -- | -- | -- |
| [M300019801](http://omad.operon.com/mouseV3/transcript.php?what=M300019801) | -- | -- | -- | -- |
| [M300018553](http://omad.operon.com/mouseV3/transcript.php?what=M300018553) | -- | -- | -- | -- |
| [M300021441](http://omad.operon.com/mouseV3/transcript.php?what=M300021441) | -- | -- | -- | -- |
| [M300015305](http://omad.operon.com/mouseV3/transcript.php?what=M300015305) | -- | -- | -- | -- |
| [M300019335](http://omad.operon.com/mouseV3/transcript.php?what=M300019335) | [Gapd](http://www.informatics.jax.org/searches/accession_report.cgi?id=MGI%3A95640) | [NM_008084](http://srs.sanger.ac.uk/srsbin/cgi-bin/wgetz?-e+%5BREFSEQ-ID:NM_008084%5D) | [AK002273](http://www.ebi.ac.uk/cgi-bin/emblfetch?AK002273) | GLYCERALDEHYDE 3-PHOSPHATE DEHYDROGENASE (EC 1.2.1.12) (GAPDH). |
| [M300020777](http://omad.operon.com/mouseV3/transcript.php?what=M300020777) | -- | -- | -- | -- |
| [M200003258](http://omad.operon.com/mouseV3/transcript.php?what=M200003258) | [Cox8a](http://www.informatics.jax.org/searches/accession_report.cgi?id=MGI%3A105959) | [NM_007750](http://srs.sanger.ac.uk/srsbin/cgi-bin/wgetz?-e+%5BREFSEQ-ID:NM_007750%5D) | [U37721](http://www.ebi.ac.uk/cgi-bin/emblfetch?U37721) | CYTOCHROME C OXIDASE POLYPEPTIDE VIII-LIVER, MITOCHONDRIAL PRECURSOR (EC 1.9.3.1). |
| [M300014515](http://omad.operon.com/mouseV3/transcript.php?what=M300014515) | -- | -- | -- | -- |
| [M300018314](http://omad.operon.com/mouseV3/transcript.php?what=M300018314) | -- | -- | -- | -- |
| [M200001083](http://omad.operon.com/mouseV3/transcript.php?what=M200001083) | [Hspa9a](http://www.informatics.jax.org/searches/accession_report.cgi?id=MGI%3A96245) | [NM_010481](http://srs.sanger.ac.uk/srsbin/cgi-bin/wgetz?-e+%5BREFSEQ-ID:NM_010481%5D) | [AK002634](http://www.ebi.ac.uk/cgi-bin/emblfetch?AK002634) | STRESS-70 PROTEIN, MITOCHONDRIAL PRECURSOR (75 KDA GLUCOSE REGULATED PROTEIN) (GRP 75) (PEPTIDE-BINDING PROTEIN 74) (PBP74) (P66 MOT) (MORTALIN). |
| [M300018559](http://omad.operon.com/mouseV3/transcript.php?what=M300018559) | -- | -- | -- | -- |
| [M300012796](http://omad.operon.com/mouseV3/transcript.php?what=M300012796) | [Hmgn1](http://www.informatics.jax.org/searches/accession_report.cgi?id=MGI%3A96120) | [NM_008251](http://srs.sanger.ac.uk/srsbin/cgi-bin/wgetz?-e+%5BREFSEQ-ID:NM_008251%5D) | [X53476](http://www.ebi.ac.uk/cgi-bin/emblfetch?X53476) | NONHISTONE CHROMOSOMAL PROTEIN HMG-14 (HIGH-MOBILITY GROUP NUCLEOSOME BINDING DOMAIN 1). |
| [M200000777](http://omad.operon.com/mouseV3/transcript.php?what=M200000777) | [G3bp-pending](http://www.informatics.jax.org/searches/accession_report.cgi?id=MGI%3A1351465) | [NM_013716](http://srs.sanger.ac.uk/srsbin/cgi-bin/wgetz?-e+%5BREFSEQ-ID:NM_013716%5D) | [AB001927](http://www.ebi.ac.uk/cgi-bin/emblfetch?AB001927) | RAS-GTPASE-ACTIVATING PROTEIN BINDING PROTEIN 1 (GAP SH3-DOMAIN BINDING PROTEIN 1) (G3BP-1). |
| [M300021668](http://omad.operon.com/mouseV3/transcript.php?what=M300021668) | -- | -- | -- | -- |
| [M300002115](http://omad.operon.com/mouseV3/transcript.php?what=M300002115) | [Xpo1](http://www.informatics.jax.org/searches/accession_report.cgi?id=MGI%3A2144013) | [NM_134014](http://srs.sanger.ac.uk/srsbin/cgi-bin/wgetz?-e+%5BREFSEQ-ID:NM_134014%5D) | [BC025628](http://www.ebi.ac.uk/cgi-bin/emblfetch?BC025628) | EXPORTIN 1, CRM1 HOMOLOG; EXPRESSED SEQUENCE AA420417. |
| [M300017554](http://omad.operon.com/mouseV3/transcript.php?what=M300017554) | [4930415K17Rik](http://www.informatics.jax.org/searches/accession_report.cgi?id=MGI%3A1914643) | [NM_133687](http://srs.sanger.ac.uk/srsbin/cgi-bin/wgetz?-e+%5BREFSEQ-ID:NM_133687%5D) | [BC016207](http://www.ebi.ac.uk/cgi-bin/emblfetch?BC016207) | -- |
| [M300004265](http://omad.operon.com/mouseV3/transcript.php?what=M300004265) | [Ms4a1](http://www.informatics.jax.org/searches/accession_report.cgi?id=MGI%3A88321) | [NM_007641](http://srs.sanger.ac.uk/srsbin/cgi-bin/wgetz?-e+%5BREFSEQ-ID:NM_007641%5D) | [AK017903](http://www.ebi.ac.uk/cgi-bin/emblfetch?AK017903) | B-CELL SURFACE PROTEIN CD20 HOMOLOG (B-CELL DIFFERENTIATION ANTIGEN LY-44). |
| [M200001144](http://omad.operon.com/mouseV3/transcript.php?what=M200001144) | [Cd79b](http://www.informatics.jax.org/searches/accession_report.cgi?id=MGI%3A96431) | [NM_008339](http://srs.sanger.ac.uk/srsbin/cgi-bin/wgetz?-e+%5BREFSEQ-ID:NM_008339%5D) | [AF002279](http://www.ebi.ac.uk/cgi-bin/emblfetch?AF002279) | B-CELL ANTIGEN RECEPTOR COMPLEX ASSOCIATED PROTEIN BETA-CHAIN PRECURSOR (B-CELL-SPECIFIC GLYCOPROTEIN B29) (IMMUNOGLOBULIN- ASSOCIATED B29 PROTEIN) (IG-BETA) (CD79B). |
| **1000 Gy** |  |  |  |  |
| [M200004687](http://omad.operon.com/mouseV3/transcript.php?what=M200004687) | [Dda3-pending](http://www.informatics.jax.org/searches/accession_report.cgi?id=MGI%3A1913099) | [NM_019976](http://srs.sanger.ac.uk/srsbin/cgi-bin/wgetz?-e+%5BREFSEQ-ID:NM_019976%5D) | [AK041835](http://www.ebi.ac.uk/cgi-bin/emblfetch?AK041835) | DIFFERENTIAL DISPLAY AND ACTIVATED BY P53; P53-REGULATED DDA3. |
| [M300008077](http://omad.operon.com/mouseV3/transcript.php?what=M300008077) | [Ei24](http://www.informatics.jax.org/searches/accession_report.cgi?id=MGI%3A108090) | [NM_007915](http://srs.sanger.ac.uk/srsbin/cgi-bin/wgetz?-e+%5BREFSEQ-ID:NM_007915%5D) | [U41751](http://www.ebi.ac.uk/cgi-bin/emblfetch?U41751) | ETOPOSIDE-INDUCED PROTEIN 2.4. |
| [M300011848](http://omad.operon.com/mouseV3/transcript.php?what=M300011848) | -- | [NM_173445](http://srs.sanger.ac.uk/srsbin/cgi-bin/wgetz?-e+%5BREFSEQ-ID:NM_173445%5D) | -- | -- |
| [M300020371](http://omad.operon.com/mouseV3/transcript.php?what=M300020371) | -- | -- | -- | -- |
| [M300019400](http://omad.operon.com/mouseV3/transcript.php?what=M300019400) | -- | -- | -- | -- |
| [M300019801](http://omad.operon.com/mouseV3/transcript.php?what=M300019801) | -- | -- | -- | -- |
| [M300014889](http://omad.operon.com/mouseV3/transcript.php?what=M300014889) | [Gapd](http://www.informatics.jax.org/searches/accession_report.cgi?id=MGI%3A95640) | [NM_008084](http://srs.sanger.ac.uk/srsbin/cgi-bin/wgetz?-e+%5BREFSEQ-ID:NM_008084%5D) | [AK002273](http://www.ebi.ac.uk/cgi-bin/emblfetch?AK002273) | GLYCERALDEHYDE 3-PHOSPHATE DEHYDROGENASE (EC 1.2.1.12) (GAPDH). |
| [M300019335](http://omad.operon.com/mouseV3/transcript.php?what=M300019335) | [Gapd](http://www.informatics.jax.org/searches/accession_report.cgi?id=MGI%3A95640) | [NM_008084](http://srs.sanger.ac.uk/srsbin/cgi-bin/wgetz?-e+%5BREFSEQ-ID:NM_008084%5D) | [AK002273](http://www.ebi.ac.uk/cgi-bin/emblfetch?AK002273) | GLYCERALDEHYDE 3-PHOSPHATE DEHYDROGENASE (EC 1.2.1.12) (GAPDH). |
| [M300000465](http://omad.operon.com/mouseV3/transcript.php?what=M300000465) | [2610301D06Rik](http://www.informatics.jax.org/searches/accession_report.cgi?id=MGI%3A1914410) | [NM_026007](http://srs.sanger.ac.uk/srsbin/cgi-bin/wgetz?-e+%5BREFSEQ-ID:NM_026007%5D) | [AK014277](http://www.ebi.ac.uk/cgi-bin/emblfetch?AK014277) | ELONGATION FACTOR 1-GAMMA (EF-1-GAMMA) (EEF-1B GAMMA). |
| [M300019589](http://omad.operon.com/mouseV3/transcript.php?what=M300019589) | -- | -- | -- | -- |
| [M300012879](http://omad.operon.com/mouseV3/transcript.php?what=M300012879) | -- | -- | [AK007389](http://www.ebi.ac.uk/cgi-bin/emblfetch?AK007389) | SMALL NUCLEAR RIBONUCLEOPROTEIN SM D2 (SNRNP CORE PROTEIN D2) (SM-D2). |
| [M300002970](http://omad.operon.com/mouseV3/transcript.php?what=M300002970) | [5730420B22Rik](http://www.informatics.jax.org/searches/accession_report.cgi?id=MGI%3A1917811) | [NM_172597](http://srs.sanger.ac.uk/srsbin/cgi-bin/wgetz?-e+%5BREFSEQ-ID:NM_172597%5D) | [AK017582](http://www.ebi.ac.uk/cgi-bin/emblfetch?AK017582) | -- |
| [M300021668](http://omad.operon.com/mouseV3/transcript.php?what=M300021668) | -- | -- | -- | -- |
| [M300011495](http://omad.operon.com/mouseV3/transcript.php?what=M300011495) | -- | -- | [BG088667](http://www.ebi.ac.uk/cgi-bin/emblfetch?BG088667) | SESTRIN 1 (P53-REGULATED PROTEIN PA26). |
| [M300017752](http://omad.operon.com/mouseV3/transcript.php?what=M300017752) | -- | -- | [AF516285](http://www.ebi.ac.uk/cgi-bin/emblfetch?AF516285) | ANTI-VIPASE LIGHT CHAIN VARIABLE REGION (FRAGMENT). |
| [M300007254](http://omad.operon.com/mouseV3/transcript.php?what=M300007254) | -- | [NM_172900](http://srs.sanger.ac.uk/srsbin/cgi-bin/wgetz?-e+%5BREFSEQ-ID:NM_172900%5D) | -- | -- |
| [M200006566](http://omad.operon.com/mouseV3/transcript.php?what=M200006566) | [Gga2](http://www.informatics.jax.org/searches/accession_report.cgi?id=MGI%3A1921355) | -- | [AK004632](http://www.ebi.ac.uk/cgi-bin/emblfetch?AK004632) | -- |
| [M200006174](http://omad.operon.com/mouseV3/transcript.php?what=M200006174) | [0610039P13Rik](http://www.informatics.jax.org/searches/accession_report.cgi?id=MGI%3A1921346) | [NM_028752](http://srs.sanger.ac.uk/srsbin/cgi-bin/wgetz?-e+%5BREFSEQ-ID:NM_028752%5D) | [BC021548](http://www.ebi.ac.uk/cgi-bin/emblfetch?BC021548) | -- |
| [M200000312](http://omad.operon.com/mouseV3/transcript.php?what=M200000312) | [Ly6d](http://www.informatics.jax.org/searches/accession_report.cgi?id=MGI%3A96881) | [NM_010742](http://srs.sanger.ac.uk/srsbin/cgi-bin/wgetz?-e+%5BREFSEQ-ID:NM_010742%5D) | [L40419](http://www.ebi.ac.uk/cgi-bin/emblfetch?L40419) | LYMPHOCYTE ANTIGEN LY-6D PRECURSOR (THYMOCYTE B CELL ANTIGEN) (THB). |
| [M200000320](http://omad.operon.com/mouseV3/transcript.php?what=M200000320) | [Pou2af1](http://www.informatics.jax.org/searches/accession_report.cgi?id=MGI%3A105086) | [NM_011136](http://srs.sanger.ac.uk/srsbin/cgi-bin/wgetz?-e+%5BREFSEQ-ID:NM_011136%5D) | [U43788](http://www.ebi.ac.uk/cgi-bin/emblfetch?U43788) | POU DOMAIN CLASS 2, ASSOCIATING FACTOR 1 (B-CELL-SPECIFIC COACTIVATOR OBF-1) (OCT BINDING FACTOR 1) (BOB-1) (BOB1) (OCA-B). |
| [M200001703](http://omad.operon.com/mouseV3/transcript.php?what=M200001703) | [Cd19](http://www.informatics.jax.org/searches/accession_report.cgi?id=MGI%3A88319) | [NM_009844](http://srs.sanger.ac.uk/srsbin/cgi-bin/wgetz?-e+%5BREFSEQ-ID:NM_009844%5D) | [M84372](http://www.ebi.ac.uk/cgi-bin/emblfetch?M84372) | B-LYMPHOCYTE ANTIGEN CD19 PRECURSOR (B-LYMPHOCYTE SURFACE ANTIGEN B4) (LEU-12) (DIFFERENTIATION ANTIGEN CD19). |
| [M200000715](http://omad.operon.com/mouseV3/transcript.php?what=M200000715) | [BB219290](http://www.informatics.jax.org/searches/accession_report.cgi?id=MGI%3A2138647) | [NM_145141](http://srs.sanger.ac.uk/srsbin/cgi-bin/wgetz?-e+%5BREFSEQ-ID:NM_145141%5D) | [AF426462](http://www.ebi.ac.uk/cgi-bin/emblfetch?AF426462) | FC RECEPTOR HOMOLOG EXPRESSED IN B CELLS; FC RECEPTOR RELATED PROTEIN X. |
| [M200002822](http://omad.operon.com/mouseV3/transcript.php?what=M200002822) | [Blnk](http://www.informatics.jax.org/searches/accession_report.cgi?id=MGI%3A96878) | [NM_008528](http://srs.sanger.ac.uk/srsbin/cgi-bin/wgetz?-e+%5BREFSEQ-ID:NM_008528%5D) | [AJ298054](http://www.ebi.ac.uk/cgi-bin/emblfetch?AJ298054) | B-CELL LINKER; LYMPHOCYTE ANTIGEN 57. |
| [M200001144](http://omad.operon.com/mouseV3/transcript.php?what=M200001144) | [Cd79b](http://www.informatics.jax.org/searches/accession_report.cgi?id=MGI%3A96431) | [NM_008339](http://srs.sanger.ac.uk/srsbin/cgi-bin/wgetz?-e+%5BREFSEQ-ID:NM_008339%5D) | [AF002279](http://www.ebi.ac.uk/cgi-bin/emblfetch?AF002279) | B-CELL ANTIGEN RECEPTOR COMPLEX ASSOCIATED PROTEIN BETA-CHAIN PRECURSOR (B-CELL-SPECIFIC GLYCOPROTEIN B29) (IMMUNOGLOBULIN- ASSOCIATED B29 PROTEIN) (IG-BETA) (CD79B). |
| [M200009317](http://omad.operon.com/mouseV3/transcript.php?what=M200009317) | [Scd1](http://www.informatics.jax.org/searches/accession_report.cgi?id=MGI%3A98239) | [NM_009127](http://srs.sanger.ac.uk/srsbin/cgi-bin/wgetz?-e+%5BREFSEQ-ID:NM_009127%5D) | [BC007474](http://www.ebi.ac.uk/cgi-bin/emblfetch?BC007474) | ACYL-COA DESATURASE 1 (EC 1.14.19.1) (STEAROYL-COA DESATURASE 1) (FATTY ACID DESATURASE 1) (DELTA(9)-DESATURASE 1). |
