# Supplementary material for: Gene Expression Signatures of Radiation Response Are Specific, Durable and Accurate in Mice and Humans
Source: PLoS One. 2008 Apr 2;3(4):e1912. doi: 10.1371/journal.pone.0001912 (PMC2271127; doi:10.1371/journal.pone.0001912)
Supplement: Table S2 — (0.09 MB DOC) [file pone.0001912.s002.doc]

Table S2. Genes that overlap between mouse groups. Operon Oligo ID can be queried in the OMAD database ([http://omad.operon.com](http://omad.operon.com/))

| **Operon OligoID** | **Gene Symbol** | **RefSeq** | **Genbank** | **Description** |
| --- | --- | --- | --- | --- |
| **SEX** |  |  |  |  |
| **C57Bl6 M and C57Bl6 F** |  |  |  |  |
| **M vs F 50cGy** |  |  |  |  |
| [M200000800](http://omad.operon.com/mouseV3/transcript.php?what=M200000800) | [Ccng](http://www.informatics.jax.org/searches/accession_report.cgi?id=MGI%3A2444813)1 | [NM_009831](http://srs.sanger.ac.uk/srsbin/cgi-bin/wgetz?-e+%5BREFSEQ-ID:NM_009831%5D) | [AB005559](http://www.ebi.ac.uk/cgi-bin/emblfetch?AB005559) | CYCLIN G1 (CYCLIN G) |
| [M200004687](http://omad.operon.com/mouseV3/transcript.php?what=M200004687) | [Dda3](http://www.informatics.jax.org/searches/accession_report.cgi?id=MGI%3A1913099) | [NM_019976](http://srs.sanger.ac.uk/srsbin/cgi-bin/wgetz?-e+%5BREFSEQ-ID:NM_019976%5D) | [AK041835](http://www.ebi.ac.uk/cgi-bin/emblfetch?AK041835) | DIFFERENTIAL DISPLAY AND ACTIVATED BY P53; P53-REGULATED DDA3. |
| **M vs F 200cGy** |  |  |  |  |
| [M200001144](http://omad.operon.com/mouseV3/transcript.php?what=M200001144) | [Cd79b](http://www.informatics.jax.org/searches/accession_report.cgi?id=MGI%3A96431) | [NM_008339](http://srs.sanger.ac.uk/srsbin/cgi-bin/wgetz?-e+%5BREFSEQ-ID:NM_008339%5D) | [AF002279](http://www.ebi.ac.uk/cgi-bin/emblfetch?AF002279) | B-CELL ANTIGEN RECEPTOR COMPLEX ASSOCIATED PROTEIN BETA-CHAIN PRECURSOR (B-CELL-SPECIFIC GLYCOPROTEIN B29) (IMMUNOGLOBULIN- ASSOCIATED B29 PROTEIN) (IG-BETA) (CD79B). |
| **M vs F 1000cGy** |  |  |  |  |
| [M200001144](http://omad.operon.com/mouseV3/transcript.php?what=M200001144) | [Cd79b](http://www.informatics.jax.org/searches/accession_report.cgi?id=MGI%3A96431) | [NM_008339](http://srs.sanger.ac.uk/srsbin/cgi-bin/wgetz?-e+%5BREFSEQ-ID:NM_008339%5D) | [AF002279](http://www.ebi.ac.uk/cgi-bin/emblfetch?AF002279) | B-CELL ANTIGEN RECEPTOR COMPLEX ASSOCIATED PROTEIN BETA-CHAIN PRECURSOR (B-CELL-SPECIFIC GLYCOPROTEIN B29) (IMMUNOGLOBULIN- ASSOCIATED B29 PROTEIN) (IG-BETA) (CD79B). |
| [M200004687](http://omad.operon.com/mouseV3/transcript.php?what=M200004687) | [Dda3](http://www.informatics.jax.org/searches/accession_report.cgi?id=MGI%3A1913099) | [NM_019976](http://srs.sanger.ac.uk/srsbin/cgi-bin/wgetz?-e+%5BREFSEQ-ID:NM_019976%5D) | [AK041835](http://www.ebi.ac.uk/cgi-bin/emblfetch?AK041835) | DIFFERENTIAL DISPLAY AND ACTIVATED BY P53; P53-REGULATED DDA3. |
| [M200009317](http://omad.operon.com/mouseV3/transcript.php?what=M200009317) | [Scd1](http://www.informatics.jax.org/searches/accession_report.cgi?id=MGI%3A98239) | [NM_009127](http://srs.sanger.ac.uk/srsbin/cgi-bin/wgetz?-e+%5BREFSEQ-ID:NM_009127%5D) | [BC007474](http://www.ebi.ac.uk/cgi-bin/emblfetch?BC007474) | ACYL-COA DESATURASE 1 (EC 1.14.19.1) (STEAROYL-COA DESATURASE 1) (FATTY ACID DESATURASE 1) (DELTA(9)-DESATURASE 1). |
| [M200006566](http://omad.operon.com/mouseV3/transcript.php?what=M200006566) | [Gga2](http://www.informatics.jax.org/searches/accession_report.cgi?id=MGI%3A1921355) | -- | [AK004632](http://www.ebi.ac.uk/cgi-bin/emblfetch?AK004632) | -- |
| M200006174 |  |  |  |  |
| M300007254 |  |  |  |  |
|  |  |  |  |  |
| **GENOTYPE** |  |  |  |  |
| **C57Bl6 F and BALB/c F** |  |  |  |  |
| **Bl vs BA 50cGy** |  |  |  |  |
| [M200000800](http://omad.operon.com/mouseV3/transcript.php?what=M200000800) | [Ccng](http://www.informatics.jax.org/searches/accession_report.cgi?id=MGI%3A2444813)1 | [NM_009831](http://srs.sanger.ac.uk/srsbin/cgi-bin/wgetz?-e+%5BREFSEQ-ID:NM_009831%5D) | [AB005559](http://www.ebi.ac.uk/cgi-bin/emblfetch?AB005559) | CYCLIN G1 (CYCLIN G) |
| [M200004687](http://omad.operon.com/mouseV3/transcript.php?what=M200004687) | [Dda3](http://www.informatics.jax.org/searches/accession_report.cgi?id=MGI%3A1913099) | [NM_019976](http://srs.sanger.ac.uk/srsbin/cgi-bin/wgetz?-e+%5BREFSEQ-ID:NM_019976%5D) | [AK041835](http://www.ebi.ac.uk/cgi-bin/emblfetch?AK041835) | DIFFERENTIAL DISPLAY AND ACTIVATED BY P53; P53-REGULATED DDA3. |
| [M300008077](http://omad.operon.com/mouseV3/transcript.php?what=M300008077) | [Ei24](http://www.informatics.jax.org/searches/accession_report.cgi?id=MGI%3A108090) | [NM_007915](http://srs.sanger.ac.uk/srsbin/cgi-bin/wgetz?-e+%5BREFSEQ-ID:NM_007915%5D) | [U41751](http://www.ebi.ac.uk/cgi-bin/emblfetch?U41751) | ETOPOSIDE-INDUCED PROTEIN 2.4. |
| **Bl vs BA 200cGy** |  |  |  |  |
| [M200004687](http://omad.operon.com/mouseV3/transcript.php?what=M200004687) | [Dda3](http://www.informatics.jax.org/searches/accession_report.cgi?id=MGI%3A1913099) | [NM_019976](http://srs.sanger.ac.uk/srsbin/cgi-bin/wgetz?-e+%5BREFSEQ-ID:NM_019976%5D) | [AK041835](http://www.ebi.ac.uk/cgi-bin/emblfetch?AK041835) | DIFFERENTIAL DISPLAY AND ACTIVATED BY P53; P53-REGULATED DDA3. |
| **Bl vs BA 1000cGy** |  |  |  |  |
| [M200004687](http://omad.operon.com/mouseV3/transcript.php?what=M200004687) | [Dda3](http://www.informatics.jax.org/searches/accession_report.cgi?id=MGI%3A1913099) | [NM_019976](http://srs.sanger.ac.uk/srsbin/cgi-bin/wgetz?-e+%5BREFSEQ-ID:NM_019976%5D) | [AK041835](http://www.ebi.ac.uk/cgi-bin/emblfetch?AK041835) | DIFFERENTIAL DISPLAY AND ACTIVATED BY P53; P53-REGULATED DDA3. |
|  |  |  |  |  |
| **TIME** |  |  |  |  |
| **Within C57Bl6 F** |  |  |  |  |
| **6hr vs 24hr 50cGy** |  |  |  |  |
| None |  |  |  |  |
| **6hr vs 24hr 200cGy** |  |  |  |  |
| None |  |  |  |  |
| **6hr vs 24hr 1000cGy** |  |  |  |  |
| None |  |  |  |  |
| **6hr vs 7d 50cGy** |  |  |  |  |
| None |  |  |  |  |
| **6hr vs 7d 200cGy** |  |  |  |  |
| None |  |  |  |  |
| **24h vs 7d 50cGy** |  |  |  |  |
| M300000165 | Lgals1 | NM_008495 | AK004298 | GALECTIN-1 (BETA-GALACTOSIDE-BINDING LECTIN L-14-I) (LACTOSE-BINDING LECTIN 1) (S-LAC LECTIN 1) |
| **24h vs 7d 200cGy** |  |  |  |  |
| None |  |  |  |  |
|  |  |  |  |  |
