# Supplementary material for: Gene Expression Signatures of Radiation Response Are Specific, Durable and Accurate in Mice and Humans
Source: PLoS One. 2008 Apr 2;3(4):e1912. doi: 10.1371/journal.pone.0001912 (PMC2271127; doi:10.1371/journal.pone.0001912)
Supplement: Table S3 — (0.20 MB DOC) [file pone.0001912.s003.doc]

Table S3. Genes that distinguish radiation responses in BALB/c mice. Operon Oligo ID can be queried in the OMAD database ([http://omad.operon.com](http://omad.operon.com/))

| **Operon OligoID** | **Gene Symbol** | **RefSeq** | **Genbank** | **Description** |
| --- | --- | --- | --- | --- |
| **50 Gy** |  |  |  |  |
| [M200013484](http://omad.operon.com/mouseV3/transcript.php?what=M200013484) | [9030617O03Rik](http://www.informatics.jax.org/searches/accession_report.cgi?id=MGI%3A2444813) | [NM_145448](http://srs.sanger.ac.uk/srsbin/cgi-bin/wgetz?-e+%5BREFSEQ-ID:NM_145448%5D) | [BC021385](http://www.ebi.ac.uk/cgi-bin/emblfetch?BC021385) | -- |
| [M200004687](http://omad.operon.com/mouseV3/transcript.php?what=M200004687) | [Dda3-pending](http://www.informatics.jax.org/searches/accession_report.cgi?id=MGI%3A1913099) | [NM_019976](http://srs.sanger.ac.uk/srsbin/cgi-bin/wgetz?-e+%5BREFSEQ-ID:NM_019976%5D) | [AK041835](http://www.ebi.ac.uk/cgi-bin/emblfetch?AK041835) | DIFFERENTIAL DISPLAY AND ACTIVATED BY P53; P53-REGULATED DDA3. |
| [M300000487](http://omad.operon.com/mouseV3/transcript.php?what=M300000487) | [Bax](http://www.informatics.jax.org/searches/accession_report.cgi?id=MGI%3A99702) | [NM_007527](http://srs.sanger.ac.uk/srsbin/cgi-bin/wgetz?-e+%5BREFSEQ-ID:NM_007527%5D) | [L22472](http://www.ebi.ac.uk/cgi-bin/emblfetch?L22472) | APOPTOSIS REGULATOR BAX, MEMBRANE ISOFORM ALPHA. |
| [M300006855](http://omad.operon.com/mouseV3/transcript.php?what=M300006855) | [Sec8](http://www.informatics.jax.org/searches/accession_report.cgi?id=MGI%3A1096376) | [NM_009148](http://srs.sanger.ac.uk/srsbin/cgi-bin/wgetz?-e+%5BREFSEQ-ID:NM_009148%5D) | [BC034644](http://www.ebi.ac.uk/cgi-bin/emblfetch?BC034644) | EXOCYST COMPLEX COMPONENT SEC8. |
| [M300001199](http://omad.operon.com/mouseV3/transcript.php?what=M300001199) | -- | -- | [BC002257](http://www.ebi.ac.uk/cgi-bin/emblfetch?BC002257) | -- |
| [M200000800](http://omad.operon.com/mouseV3/transcript.php?what=M200000800) | [Ccng1](http://www.informatics.jax.org/searches/accession_report.cgi?id=MGI%3A102890) | [NM_009831](http://srs.sanger.ac.uk/srsbin/cgi-bin/wgetz?-e+%5BREFSEQ-ID:NM_009831%5D) | [AB005559](http://www.ebi.ac.uk/cgi-bin/emblfetch?AB005559) | CYCLIN G1 (CYCLIN G). |
| [M200016031](http://omad.operon.com/mouseV3/transcript.php?what=M200016031) | [Polk](http://www.informatics.jax.org/searches/accession_report.cgi?id=MGI%3A1349767) | [NM_012048](http://srs.sanger.ac.uk/srsbin/cgi-bin/wgetz?-e+%5BREFSEQ-ID:NM_012048%5D) | [AB040764](http://www.ebi.ac.uk/cgi-bin/emblfetch?AB040764) | POLYMERASE (DNA DIRECTED), KAPPA; DINB HOMOLOG 1 (E. COLI); DNA DAMAGE-INDUCIBLE PROETIN B; DNA DAMAGE-INDUCIBLE PROTEIN B; POLYMERASE (DNA DIRECTED) KAPPA. |
| [M200003784](http://omad.operon.com/mouseV3/transcript.php?what=M200003784) | [Bax](http://www.informatics.jax.org/searches/accession_report.cgi?id=MGI%3A99702) | [NM_007527](http://srs.sanger.ac.uk/srsbin/cgi-bin/wgetz?-e+%5BREFSEQ-ID:NM_007527%5D) | [L22472](http://www.ebi.ac.uk/cgi-bin/emblfetch?L22472) | APOPTOSIS REGULATOR BAX, MEMBRANE ISOFORM ALPHA. |
| [M200007547](http://omad.operon.com/mouseV3/transcript.php?what=M200007547) | [Phlda3](http://www.informatics.jax.org/searches/accession_report.cgi?id=MGI%3A1351485) | [NM_013750](http://srs.sanger.ac.uk/srsbin/cgi-bin/wgetz?-e+%5BREFSEQ-ID:NM_013750%5D) | [BC023408](http://www.ebi.ac.uk/cgi-bin/emblfetch?BC023408) | PLECKSTRIN HOMOLOGY-LIKE DOMAIN, FAMILY A, MEMBER 3; TDAG/LPL HOMOLOG 1. |
| [M300010491](http://omad.operon.com/mouseV3/transcript.php?what=M300010491) | [D030041N15Rik](http://www.informatics.jax.org/searches/accession_report.cgi?id=MGI%3A2443767) | [NM_153416](http://srs.sanger.ac.uk/srsbin/cgi-bin/wgetz?-e+%5BREFSEQ-ID:NM_153416%5D) | [BC018191](http://www.ebi.ac.uk/cgi-bin/emblfetch?BC018191) | ALADIN (ADRACALIN). |
| [M200006364](http://omad.operon.com/mouseV3/transcript.php?what=M200006364) | [Dcxr](http://www.informatics.jax.org/searches/accession_report.cgi?id=MGI%3A1915130) | [NM_026428](http://srs.sanger.ac.uk/srsbin/cgi-bin/wgetz?-e+%5BREFSEQ-ID:NM_026428%5D) | [AK004023](http://www.ebi.ac.uk/cgi-bin/emblfetch?AK004023) | DIACETYL/L-XYLULOSE REDUCTASE. |
| [M300007324](http://omad.operon.com/mouseV3/transcript.php?what=M300007324) | [2700083B06Rik](http://www.informatics.jax.org/searches/accession_report.cgi?id=MGI%3A1915298) | [NM_026531](http://srs.sanger.ac.uk/srsbin/cgi-bin/wgetz?-e+%5BREFSEQ-ID:NM_026531%5D) | [BC022614](http://www.ebi.ac.uk/cgi-bin/emblfetch?BC022614) | -- |
| [M300000486](http://omad.operon.com/mouseV3/transcript.php?what=M300000486) | [Bax](http://www.informatics.jax.org/searches/accession_report.cgi?id=MGI%3A99702) | [NM_007527](http://srs.sanger.ac.uk/srsbin/cgi-bin/wgetz?-e+%5BREFSEQ-ID:NM_007527%5D) | [L22472](http://www.ebi.ac.uk/cgi-bin/emblfetch?L22472) | APOPTOSIS REGULATOR BAX, MEMBRANE ISOFORM ALPHA. |
| [M200007794](http://omad.operon.com/mouseV3/transcript.php?what=M200007794) | [Wig1](http://www.informatics.jax.org/searches/accession_report.cgi?id=MGI%3A1195270) | [NM_009517](http://srs.sanger.ac.uk/srsbin/cgi-bin/wgetz?-e+%5BREFSEQ-ID:NM_009517%5D) | [AF012923](http://www.ebi.ac.uk/cgi-bin/emblfetch?AF012923) | WILD-TYPE P53-INDUCED GENE 1. |
| [M300008077](http://omad.operon.com/mouseV3/transcript.php?what=M300008077) | [Ei24](http://www.informatics.jax.org/searches/accession_report.cgi?id=MGI%3A108090) | [NM_007915](http://srs.sanger.ac.uk/srsbin/cgi-bin/wgetz?-e+%5BREFSEQ-ID:NM_007915%5D) | [U41751](http://www.ebi.ac.uk/cgi-bin/emblfetch?U41751) | ETOPOSIDE-INDUCED PROTEIN 2.4. |
| [M300003395](http://omad.operon.com/mouseV3/transcript.php?what=M300003395) | [Ly6e](http://www.informatics.jax.org/searches/accession_report.cgi?id=MGI%3A106651) | [NM_008529](http://srs.sanger.ac.uk/srsbin/cgi-bin/wgetz?-e+%5BREFSEQ-ID:NM_008529%5D) | [U47737](http://www.ebi.ac.uk/cgi-bin/emblfetch?U47737) | LYMPHOCYTE ANTIGEN LY-6E PRECURSOR (THYMIC SHARED ANTIGEN-1) (TSA-1) (STEM CELL ANTIGEN 2). |
| [M200003474](http://omad.operon.com/mouseV3/transcript.php?what=M200003474) | [D730042P09Rik](http://www.informatics.jax.org/searches/accession_report.cgi?id=MGI%3A1925112) | [NM_144543](http://srs.sanger.ac.uk/srsbin/cgi-bin/wgetz?-e+%5BREFSEQ-ID:NM_144543%5D) | [AB080370](http://www.ebi.ac.uk/cgi-bin/emblfetch?AB080370) | THYMOCYTE PROTEIN THY28. |
| [M200012250](http://omad.operon.com/mouseV3/transcript.php?what=M200012250) | [Scd2](http://www.informatics.jax.org/searches/accession_report.cgi?id=MGI%3A98240) | [NM_009128](http://srs.sanger.ac.uk/srsbin/cgi-bin/wgetz?-e+%5BREFSEQ-ID:NM_009128%5D) | [M26270](http://www.ebi.ac.uk/cgi-bin/emblfetch?M26270) | ACYL-COA DESATURASE 2 (EC 1.14.19.1) (STEAROYL-COA DESATURASE 2) (FATTY ACID DESATURASE 2) (DELTA(9)-DESATURASE 2). |
| [M200000655](http://omad.operon.com/mouseV3/transcript.php?what=M200000655) | [Tnfrsf6](http://www.informatics.jax.org/searches/accession_report.cgi?id=MGI%3A95484) | [NM_007987](http://srs.sanger.ac.uk/srsbin/cgi-bin/wgetz?-e+%5BREFSEQ-ID:NM_007987%5D) | [S56486](http://www.ebi.ac.uk/cgi-bin/emblfetch?S56486) | TUMOR NECROSIS FACTOR RECEPTOR SUPERFAMILY MEMBER 6 PRECURSOR (FASL RECEPTOR) (APOPTOSIS-MEDIATING SURFACE ANTIGEN FAS) (APO-1 ANTIGEN) (CD95). |
| [M200008006](http://omad.operon.com/mouseV3/transcript.php?what=M200008006) | [2410089B13Rik](http://www.informatics.jax.org/searches/accession_report.cgi?id=MGI%3A1920939) | -- | [AK010745](http://www.ebi.ac.uk/cgi-bin/emblfetch?AK010745) | -- |
| [M200000279](http://omad.operon.com/mouseV3/transcript.php?what=M200000279) | [Ly6e](http://www.informatics.jax.org/searches/accession_report.cgi?id=MGI%3A106651) | [NM_008529](http://srs.sanger.ac.uk/srsbin/cgi-bin/wgetz?-e+%5BREFSEQ-ID:NM_008529%5D) | [U47737](http://www.ebi.ac.uk/cgi-bin/emblfetch?U47737) | LYMPHOCYTE ANTIGEN LY-6E PRECURSOR (THYMIC SHARED ANTIGEN-1) (TSA-1) (STEM CELL ANTIGEN 2). |
| [M200000354](http://omad.operon.com/mouseV3/transcript.php?what=M200000354) | [ORF21](http://www.informatics.jax.org/searches/accession_report.cgi?id=MGI%3A2136890) | [NM_145482](http://srs.sanger.ac.uk/srsbin/cgi-bin/wgetz?-e+%5BREFSEQ-ID:NM_145482%5D) | [BC029101](http://www.ebi.ac.uk/cgi-bin/emblfetch?BC029101) | -- |
| [M300002140](http://omad.operon.com/mouseV3/transcript.php?what=M300002140) | [D11Ertd603e](http://www.informatics.jax.org/searches/accession_report.cgi?id=MGI%3A1277103) | [NM_026023](http://srs.sanger.ac.uk/srsbin/cgi-bin/wgetz?-e+%5BREFSEQ-ID:NM_026023%5D) | [AK004388](http://www.ebi.ac.uk/cgi-bin/emblfetch?AK004388) | -- |
| [M300002232](http://omad.operon.com/mouseV3/transcript.php?what=M300002232) | [Ppm1d](http://www.informatics.jax.org/searches/accession_report.cgi?id=MGI%3A1858214) | [NM_016910](http://srs.sanger.ac.uk/srsbin/cgi-bin/wgetz?-e+%5BREFSEQ-ID:NM_016910%5D) | [AF200464](http://www.ebi.ac.uk/cgi-bin/emblfetch?AF200464) | PROTEIN PHOSPHATASE 2C DELTA ISOFORM (EC 3.1.3.16) (PP2C-DELTA) (P53- INDUCED PROTEIN PHOSPHATASE 1) (PROTEIN PHOSPHATASE MAGNESIUM- DEPENDENT 1 DELTA). |
| [M300002800](http://omad.operon.com/mouseV3/transcript.php?what=M300002800) | [Zfp369](http://www.informatics.jax.org/searches/accession_report.cgi?id=MGI%3A2176229) | -- | [BC036565](http://www.ebi.ac.uk/cgi-bin/emblfetch?BC036565) | NEUROTROPHIN RECEPTOR INTERACTING FACTOR 2. |
| **200 Gy** |  |  |  |  |
| [M200004687](http://omad.operon.com/mouseV3/transcript.php?what=M200004687) | [Dda3-pending](http://www.informatics.jax.org/searches/accession_report.cgi?id=MGI%3A1913099) | [NM_019976](http://srs.sanger.ac.uk/srsbin/cgi-bin/wgetz?-e+%5BREFSEQ-ID:NM_019976%5D) | [AK041835](http://www.ebi.ac.uk/cgi-bin/emblfetch?AK041835) | DIFFERENTIAL DISPLAY AND ACTIVATED BY P53; P53-REGULATED DDA3. |
| [M300020088](http://omad.operon.com/mouseV3/transcript.php?what=M300020088) | -- | -- | -- | -- |
| [M300004256](http://omad.operon.com/mouseV3/transcript.php?what=M300004256) | [Fth](http://www.informatics.jax.org/searches/accession_report.cgi?id=MGI%3A95588) | [NM_010239](http://srs.sanger.ac.uk/srsbin/cgi-bin/wgetz?-e+%5BREFSEQ-ID:NM_010239%5D) | [M24509](http://www.ebi.ac.uk/cgi-bin/emblfetch?M24509) | FERRITIN HEAVY CHAIN (FERRITIN H SUBUNIT). |
| [M300014099](http://omad.operon.com/mouseV3/transcript.php?what=M300014099) | [Actl](http://www.informatics.jax.org/searches/accession_report.cgi?id=MGI%3A109429) | [NM_013798](http://srs.sanger.ac.uk/srsbin/cgi-bin/wgetz?-e+%5BREFSEQ-ID:NM_013798%5D) | [AF195094](http://www.ebi.ac.uk/cgi-bin/emblfetch?AF195094) | ACTIN-LIKE. |
| [M300020371](http://omad.operon.com/mouseV3/transcript.php?what=M300020371) | -- | -- | -- | -- |
| [M200006851](http://omad.operon.com/mouseV3/transcript.php?what=M200006851) | -- | [NM_026467](http://srs.sanger.ac.uk/srsbin/cgi-bin/wgetz?-e+%5BREFSEQ-ID:NM_026467%5D) | -- | RIBOSOMAL PROTEIN S27-LIKE. |
| [M300015889](http://omad.operon.com/mouseV3/transcript.php?what=M300015889) | -- | -- | -- | -- |
| [M300019801](http://omad.operon.com/mouseV3/transcript.php?what=M300019801) | -- | -- | -- | -- |
| [M300018553](http://omad.operon.com/mouseV3/transcript.php?what=M300018553) | -- | -- | -- | -- |
| [M300021441](http://omad.operon.com/mouseV3/transcript.php?what=M300021441) | -- | -- | -- | -- |
| [M300015305](http://omad.operon.com/mouseV3/transcript.php?what=M300015305) | -- | -- | -- | -- |
| [M300019335](http://omad.operon.com/mouseV3/transcript.php?what=M300019335) | [Gapd](http://www.informatics.jax.org/searches/accession_report.cgi?id=MGI%3A95640) | [NM_008084](http://srs.sanger.ac.uk/srsbin/cgi-bin/wgetz?-e+%5BREFSEQ-ID:NM_008084%5D) | [AK002273](http://www.ebi.ac.uk/cgi-bin/emblfetch?AK002273) | GLYCERALDEHYDE 3-PHOSPHATE DEHYDROGENASE (EC 1.2.1.12) (GAPDH). |
| [M300020777](http://omad.operon.com/mouseV3/transcript.php?what=M300020777) | -- | -- | -- | -- |
| [M200003258](http://omad.operon.com/mouseV3/transcript.php?what=M200003258) | [Cox8a](http://www.informatics.jax.org/searches/accession_report.cgi?id=MGI%3A105959) | [NM_007750](http://srs.sanger.ac.uk/srsbin/cgi-bin/wgetz?-e+%5BREFSEQ-ID:NM_007750%5D) | [U37721](http://www.ebi.ac.uk/cgi-bin/emblfetch?U37721) | CYTOCHROME C OXIDASE POLYPEPTIDE VIII-LIVER, MITOCHONDRIAL PRECURSOR (EC 1.9.3.1). |
| [M300014515](http://omad.operon.com/mouseV3/transcript.php?what=M300014515) | -- | -- | -- | -- |
| [M300018314](http://omad.operon.com/mouseV3/transcript.php?what=M300018314) | -- | -- | -- | -- |
| [M200001083](http://omad.operon.com/mouseV3/transcript.php?what=M200001083) | [Hspa9a](http://www.informatics.jax.org/searches/accession_report.cgi?id=MGI%3A96245) | [NM_010481](http://srs.sanger.ac.uk/srsbin/cgi-bin/wgetz?-e+%5BREFSEQ-ID:NM_010481%5D) | [AK002634](http://www.ebi.ac.uk/cgi-bin/emblfetch?AK002634) | STRESS-70 PROTEIN, MITOCHONDRIAL PRECURSOR (75 KDA GLUCOSE REGULATED PROTEIN) (GRP 75) (PEPTIDE-BINDING PROTEIN 74) (PBP74) (P66 MOT) (MORTALIN). |
| [M300018559](http://omad.operon.com/mouseV3/transcript.php?what=M300018559) | -- | -- | -- | -- |
| [M300012796](http://omad.operon.com/mouseV3/transcript.php?what=M300012796) | [Hmgn1](http://www.informatics.jax.org/searches/accession_report.cgi?id=MGI%3A96120) | [NM_008251](http://srs.sanger.ac.uk/srsbin/cgi-bin/wgetz?-e+%5BREFSEQ-ID:NM_008251%5D) | [X53476](http://www.ebi.ac.uk/cgi-bin/emblfetch?X53476) | NONHISTONE CHROMOSOMAL PROTEIN HMG-14 (HIGH-MOBILITY GROUP NUCLEOSOME BINDING DOMAIN 1). |
| [M200000777](http://omad.operon.com/mouseV3/transcript.php?what=M200000777) | [G3bp-pending](http://www.informatics.jax.org/searches/accession_report.cgi?id=MGI%3A1351465) | [NM_013716](http://srs.sanger.ac.uk/srsbin/cgi-bin/wgetz?-e+%5BREFSEQ-ID:NM_013716%5D) | [AB001927](http://www.ebi.ac.uk/cgi-bin/emblfetch?AB001927) | RAS-GTPASE-ACTIVATING PROTEIN BINDING PROTEIN 1 (GAP SH3-DOMAIN BINDING PROTEIN 1) (G3BP-1). |
| [M300021668](http://omad.operon.com/mouseV3/transcript.php?what=M300021668) | -- | -- | -- | -- |
| [M300002115](http://omad.operon.com/mouseV3/transcript.php?what=M300002115) | [Xpo1](http://www.informatics.jax.org/searches/accession_report.cgi?id=MGI%3A2144013) | [NM_134014](http://srs.sanger.ac.uk/srsbin/cgi-bin/wgetz?-e+%5BREFSEQ-ID:NM_134014%5D) | [BC025628](http://www.ebi.ac.uk/cgi-bin/emblfetch?BC025628) | EXPORTIN 1, CRM1 HOMOLOG; EXPRESSED SEQUENCE AA420417. |
| [M300017554](http://omad.operon.com/mouseV3/transcript.php?what=M300017554) | [4930415K17Rik](http://www.informatics.jax.org/searches/accession_report.cgi?id=MGI%3A1914643) | [NM_133687](http://srs.sanger.ac.uk/srsbin/cgi-bin/wgetz?-e+%5BREFSEQ-ID:NM_133687%5D) | [BC016207](http://www.ebi.ac.uk/cgi-bin/emblfetch?BC016207) | -- |
| [M300004265](http://omad.operon.com/mouseV3/transcript.php?what=M300004265) | [Ms4a1](http://www.informatics.jax.org/searches/accession_report.cgi?id=MGI%3A88321) | [NM_007641](http://srs.sanger.ac.uk/srsbin/cgi-bin/wgetz?-e+%5BREFSEQ-ID:NM_007641%5D) | [AK017903](http://www.ebi.ac.uk/cgi-bin/emblfetch?AK017903) | B-CELL SURFACE PROTEIN CD20 HOMOLOG (B-CELL DIFFERENTIATION ANTIGEN LY-44). |
| [M200001144](http://omad.operon.com/mouseV3/transcript.php?what=M200001144) | [Cd79b](http://www.informatics.jax.org/searches/accession_report.cgi?id=MGI%3A96431) | [NM_008339](http://srs.sanger.ac.uk/srsbin/cgi-bin/wgetz?-e+%5BREFSEQ-ID:NM_008339%5D) | [AF002279](http://www.ebi.ac.uk/cgi-bin/emblfetch?AF002279) | B-CELL ANTIGEN RECEPTOR COMPLEX ASSOCIATED PROTEIN BETA-CHAIN PRECURSOR (B-CELL-SPECIFIC GLYCOPROTEIN B29) (IMMUNOGLOBULIN- ASSOCIATED B29 PROTEIN) (IG-BETA) (CD79B). |
| **1000 Gy** |  |  |  |  |
| [M200004687](http://omad.operon.com/mouseV3/transcript.php?what=M200004687) | [Dda3-pending](http://www.informatics.jax.org/searches/accession_report.cgi?id=MGI%3A1913099) | [NM_019976](http://srs.sanger.ac.uk/srsbin/cgi-bin/wgetz?-e+%5BREFSEQ-ID:NM_019976%5D) | [AK041835](http://www.ebi.ac.uk/cgi-bin/emblfetch?AK041835) | DIFFERENTIAL DISPLAY AND ACTIVATED BY P53; P53-REGULATED DDA3. |
| [M300008077](http://omad.operon.com/mouseV3/transcript.php?what=M300008077) | [Ei24](http://www.informatics.jax.org/searches/accession_report.cgi?id=MGI%3A108090) | [NM_007915](http://srs.sanger.ac.uk/srsbin/cgi-bin/wgetz?-e+%5BREFSEQ-ID:NM_007915%5D) | [U41751](http://www.ebi.ac.uk/cgi-bin/emblfetch?U41751) | ETOPOSIDE-INDUCED PROTEIN 2.4. |
| [M300011848](http://omad.operon.com/mouseV3/transcript.php?what=M300011848) | -- | [NM_173445](http://srs.sanger.ac.uk/srsbin/cgi-bin/wgetz?-e+%5BREFSEQ-ID:NM_173445%5D) | -- | -- |
| [M300020371](http://omad.operon.com/mouseV3/transcript.php?what=M300020371) | -- | -- | -- | -- |
| [M300019400](http://omad.operon.com/mouseV3/transcript.php?what=M300019400) | -- | -- | -- | -- |
| [M300019801](http://omad.operon.com/mouseV3/transcript.php?what=M300019801) | -- | -- | -- | -- |
| [M300014889](http://omad.operon.com/mouseV3/transcript.php?what=M300014889) | [Gapd](http://www.informatics.jax.org/searches/accession_report.cgi?id=MGI%3A95640) | [NM_008084](http://srs.sanger.ac.uk/srsbin/cgi-bin/wgetz?-e+%5BREFSEQ-ID:NM_008084%5D) | [AK002273](http://www.ebi.ac.uk/cgi-bin/emblfetch?AK002273) | GLYCERALDEHYDE 3-PHOSPHATE DEHYDROGENASE (EC 1.2.1.12) (GAPDH). |
| [M300019335](http://omad.operon.com/mouseV3/transcript.php?what=M300019335) | [Gapd](http://www.informatics.jax.org/searches/accession_report.cgi?id=MGI%3A95640) | [NM_008084](http://srs.sanger.ac.uk/srsbin/cgi-bin/wgetz?-e+%5BREFSEQ-ID:NM_008084%5D) | [AK002273](http://www.ebi.ac.uk/cgi-bin/emblfetch?AK002273) | GLYCERALDEHYDE 3-PHOSPHATE DEHYDROGENASE (EC 1.2.1.12) (GAPDH). |
| [M300000465](http://omad.operon.com/mouseV3/transcript.php?what=M300000465) | [2610301D06Rik](http://www.informatics.jax.org/searches/accession_report.cgi?id=MGI%3A1914410) | [NM_026007](http://srs.sanger.ac.uk/srsbin/cgi-bin/wgetz?-e+%5BREFSEQ-ID:NM_026007%5D) | [AK014277](http://www.ebi.ac.uk/cgi-bin/emblfetch?AK014277) | ELONGATION FACTOR 1-GAMMA (EF-1-GAMMA) (EEF-1B GAMMA). |
| [M300019589](http://omad.operon.com/mouseV3/transcript.php?what=M300019589) | -- | -- | -- | -- |
| [M300012879](http://omad.operon.com/mouseV3/transcript.php?what=M300012879) | -- | -- | [AK007389](http://www.ebi.ac.uk/cgi-bin/emblfetch?AK007389) | SMALL NUCLEAR RIBONUCLEOPROTEIN SM D2 (SNRNP CORE PROTEIN D2) (SM-D2). |
| [M300002970](http://omad.operon.com/mouseV3/transcript.php?what=M300002970) | [5730420B22Rik](http://www.informatics.jax.org/searches/accession_report.cgi?id=MGI%3A1917811) | [NM_172597](http://srs.sanger.ac.uk/srsbin/cgi-bin/wgetz?-e+%5BREFSEQ-ID:NM_172597%5D) | [AK017582](http://www.ebi.ac.uk/cgi-bin/emblfetch?AK017582) | -- |
| [M300021668](http://omad.operon.com/mouseV3/transcript.php?what=M300021668) | -- | -- | -- | -- |
| [M300011495](http://omad.operon.com/mouseV3/transcript.php?what=M300011495) | -- | -- | [BG088667](http://www.ebi.ac.uk/cgi-bin/emblfetch?BG088667) | SESTRIN 1 (P53-REGULATED PROTEIN PA26). |
| [M300017752](http://omad.operon.com/mouseV3/transcript.php?what=M300017752) | -- | -- | [AF516285](http://www.ebi.ac.uk/cgi-bin/emblfetch?AF516285) | ANTI-VIPASE LIGHT CHAIN VARIABLE REGION (FRAGMENT). |
| [M300007254](http://omad.operon.com/mouseV3/transcript.php?what=M300007254) | -- | [NM_172900](http://srs.sanger.ac.uk/srsbin/cgi-bin/wgetz?-e+%5BREFSEQ-ID:NM_172900%5D) | -- | -- |
| [M200006566](http://omad.operon.com/mouseV3/transcript.php?what=M200006566) | [Gga2](http://www.informatics.jax.org/searches/accession_report.cgi?id=MGI%3A1921355) | -- | [AK004632](http://www.ebi.ac.uk/cgi-bin/emblfetch?AK004632) | -- |
| [M200006174](http://omad.operon.com/mouseV3/transcript.php?what=M200006174) | [0610039P13Rik](http://www.informatics.jax.org/searches/accession_report.cgi?id=MGI%3A1921346) | [NM_028752](http://srs.sanger.ac.uk/srsbin/cgi-bin/wgetz?-e+%5BREFSEQ-ID:NM_028752%5D) | [BC021548](http://www.ebi.ac.uk/cgi-bin/emblfetch?BC021548) | -- |
| [M200000312](http://omad.operon.com/mouseV3/transcript.php?what=M200000312) | [Ly6d](http://www.informatics.jax.org/searches/accession_report.cgi?id=MGI%3A96881) | [NM_010742](http://srs.sanger.ac.uk/srsbin/cgi-bin/wgetz?-e+%5BREFSEQ-ID:NM_010742%5D) | [L40419](http://www.ebi.ac.uk/cgi-bin/emblfetch?L40419) | LYMPHOCYTE ANTIGEN LY-6D PRECURSOR (THYMOCYTE B CELL ANTIGEN) (THB). |
| [M200000320](http://omad.operon.com/mouseV3/transcript.php?what=M200000320) | [Pou2af1](http://www.informatics.jax.org/searches/accession_report.cgi?id=MGI%3A105086) | [NM_011136](http://srs.sanger.ac.uk/srsbin/cgi-bin/wgetz?-e+%5BREFSEQ-ID:NM_011136%5D) | [U43788](http://www.ebi.ac.uk/cgi-bin/emblfetch?U43788) | POU DOMAIN CLASS 2, ASSOCIATING FACTOR 1 (B-CELL-SPECIFIC COACTIVATOR OBF-1) (OCT BINDING FACTOR 1) (BOB-1) (BOB1) (OCA-B). |
| [M200001703](http://omad.operon.com/mouseV3/transcript.php?what=M200001703) | [Cd19](http://www.informatics.jax.org/searches/accession_report.cgi?id=MGI%3A88319) | [NM_009844](http://srs.sanger.ac.uk/srsbin/cgi-bin/wgetz?-e+%5BREFSEQ-ID:NM_009844%5D) | [M84372](http://www.ebi.ac.uk/cgi-bin/emblfetch?M84372) | B-LYMPHOCYTE ANTIGEN CD19 PRECURSOR (B-LYMPHOCYTE SURFACE ANTIGEN B4) (LEU-12) (DIFFERENTIATION ANTIGEN CD19). |
| [M200000715](http://omad.operon.com/mouseV3/transcript.php?what=M200000715) | [BB219290](http://www.informatics.jax.org/searches/accession_report.cgi?id=MGI%3A2138647) | [NM_145141](http://srs.sanger.ac.uk/srsbin/cgi-bin/wgetz?-e+%5BREFSEQ-ID:NM_145141%5D) | [AF426462](http://www.ebi.ac.uk/cgi-bin/emblfetch?AF426462) | FC RECEPTOR HOMOLOG EXPRESSED IN B CELLS; FC RECEPTOR RELATED PROTEIN X. |
| [M200002822](http://omad.operon.com/mouseV3/transcript.php?what=M200002822) | [Blnk](http://www.informatics.jax.org/searches/accession_report.cgi?id=MGI%3A96878) | [NM_008528](http://srs.sanger.ac.uk/srsbin/cgi-bin/wgetz?-e+%5BREFSEQ-ID:NM_008528%5D) | [AJ298054](http://www.ebi.ac.uk/cgi-bin/emblfetch?AJ298054) | B-CELL LINKER; LYMPHOCYTE ANTIGEN 57. |
| [M200001144](http://omad.operon.com/mouseV3/transcript.php?what=M200001144) | [Cd79b](http://www.informatics.jax.org/searches/accession_report.cgi?id=MGI%3A96431) | [NM_008339](http://srs.sanger.ac.uk/srsbin/cgi-bin/wgetz?-e+%5BREFSEQ-ID:NM_008339%5D) | [AF002279](http://www.ebi.ac.uk/cgi-bin/emblfetch?AF002279) | B-CELL ANTIGEN RECEPTOR COMPLEX ASSOCIATED PROTEIN BETA-CHAIN PRECURSOR (B-CELL-SPECIFIC GLYCOPROTEIN B29) (IMMUNOGLOBULIN- ASSOCIATED B29 PROTEIN) (IG-BETA) (CD79B). |
| [M200009317](http://omad.operon.com/mouseV3/transcript.php?what=M200009317) | [Scd1](http://www.informatics.jax.org/searches/accession_report.cgi?id=MGI%3A98239) | [NM_009127](http://srs.sanger.ac.uk/srsbin/cgi-bin/wgetz?-e+%5BREFSEQ-ID:NM_009127%5D) | [BC007474](http://www.ebi.ac.uk/cgi-bin/emblfetch?BC007474) | ACYL-COA DESATURASE 1 (EC 1.14.19.1) (STEAROYL-COA DESATURASE 1) (FATTY ACID DESATURASE 1) (DELTA(9)-DESATURASE 1). |
