# Supplementary material for: Gene Expression Signatures of Radiation Response Are Specific, Durable and Accurate in Mice and Humans
Source: PLoS One. 2008 Apr 2;3(4):e1912. doi: 10.1371/journal.pone.0001912 (PMC2271127; doi:10.1371/journal.pone.0001912)
Supplement: Table S4 — (0.49 MB DOC) [file pone.0001912.s004.doc]

Table S4. Genes that distinguish the impact of time in C57Bl6 mice. Operon Oligo ID can be queried in the OMAD database ([http://omad.operon.com](http://omad.operon.com/))

| **Operon Oligo ID** | **Gene Symbol** | **RefSeq** | **Genbank** | **Description** |
| --- | --- | --- | --- | --- |
| **Female C57Bl6 6hr 50 cGy** |  |  |  |  |
| [M300002291](http://omad.operon.com/mouseV3/transcript.php?what=M300002291) | -- | -- | -- | -- |
| [M200004687](http://omad.operon.com/mouseV3/transcript.php?what=M200004687) | [Dda3-pending](http://www.informatics.jax.org/searches/accession_report.cgi?id=MGI%3A1913099) | [NM_019976](http://srs.sanger.ac.uk/srsbin/cgi-bin/wgetz?-e+%5BREFSEQ-ID:NM_019976%5D) | [AK041835](http://www.ebi.ac.uk/cgi-bin/emblfetch?AK041835) | DIFFERENTIAL DISPLAY AND ACTIVATED BY P53; P53-REGULATED DDA3. |
| [M200000800](http://omad.operon.com/mouseV3/transcript.php?what=M200000800) | [Ccng1](http://www.informatics.jax.org/searches/accession_report.cgi?id=MGI%3A102890) | [NM_009831](http://srs.sanger.ac.uk/srsbin/cgi-bin/wgetz?-e+%5BREFSEQ-ID:NM_009831%5D) | [AB005559](http://www.ebi.ac.uk/cgi-bin/emblfetch?AB005559) | CYCLIN G1 (CYCLIN G). |
| [M300016629](http://omad.operon.com/mouseV3/transcript.php?what=M300016629) | -- | -- | -- | -- |
| [M300020491](http://omad.operon.com/mouseV3/transcript.php?what=M300020491) | -- | -- | [U38498](http://www.ebi.ac.uk/cgi-bin/emblfetch?U38498) | GUANINE NUCLEOTIDE-BINDING PROTEIN G(I)/G(S)/G(O) GAMMA-5 SUBUNIT. |
| [M300015969](http://omad.operon.com/mouseV3/transcript.php?what=M300015969) | -- | -- | -- | -- |
| [M300010063](http://omad.operon.com/mouseV3/transcript.php?what=M300010063) | -- | -- | -- | -- |
| [M300016018](http://omad.operon.com/mouseV3/transcript.php?what=M300016018) | -- | [NM_023133](http://srs.sanger.ac.uk/srsbin/cgi-bin/wgetz?-e+%5BREFSEQ-ID:NM_023133%5D) | -- | RIBOSOMAL PROTEIN S19. |
| [M200002378](http://omad.operon.com/mouseV3/transcript.php?what=M200002378) | [S100a13](http://www.informatics.jax.org/searches/accession_report.cgi?id=MGI%3A109581) | [NM_009113](http://srs.sanger.ac.uk/srsbin/cgi-bin/wgetz?-e+%5BREFSEQ-ID:NM_009113%5D) | [BC005687](http://www.ebi.ac.uk/cgi-bin/emblfetch?BC005687) | S100 CALCIUM-BINDING PROTEIN A13. |
| [M300019659](http://omad.operon.com/mouseV3/transcript.php?what=M300019659) | -- | -- | -- | -- |
| [M300014141](http://omad.operon.com/mouseV3/transcript.php?what=M300014141) | [V1rc22](http://www.informatics.jax.org/searches/accession_report.cgi?id=MGI%3A2159458) | [NM_134177](http://srs.sanger.ac.uk/srsbin/cgi-bin/wgetz?-e+%5BREFSEQ-ID:NM_134177%5D) | [AY065478](http://www.ebi.ac.uk/cgi-bin/emblfetch?AY065478) | VOMERONASAL 1 RECEPTOR, C22. |
| [M300020488](http://omad.operon.com/mouseV3/transcript.php?what=M300020488) | -- | -- | [V00754](http://www.ebi.ac.uk/cgi-bin/emblfetch?V00754) | HISTONE H3.4 (EMBRYONIC). |
| [M300019012](http://omad.operon.com/mouseV3/transcript.php?what=M300019012) | -- | -- | -- | -- |
| [M300014338](http://omad.operon.com/mouseV3/transcript.php?what=M300014338) | -- | -- | -- | -- |
| [M300009287](http://omad.operon.com/mouseV3/transcript.php?what=M300009287) | -- | -- | -- | -- |
| [M300002125](http://omad.operon.com/mouseV3/transcript.php?what=M300002125) | -- | -- | -- | -- |
| [M300008077](http://omad.operon.com/mouseV3/transcript.php?what=M300008077) | [Ei24](http://www.informatics.jax.org/searches/accession_report.cgi?id=MGI%3A108090) | [NM_007915](http://srs.sanger.ac.uk/srsbin/cgi-bin/wgetz?-e+%5BREFSEQ-ID:NM_007915%5D) | [U41751](http://www.ebi.ac.uk/cgi-bin/emblfetch?U41751) | ETOPOSIDE-INDUCED PROTEIN 2.4. |
| [M200006774](http://omad.operon.com/mouseV3/transcript.php?what=M200006774) | [2400001E08Rik](http://www.informatics.jax.org/searches/accession_report.cgi?id=MGI%3A1913758) | [NM_025605](http://srs.sanger.ac.uk/srsbin/cgi-bin/wgetz?-e+%5BREFSEQ-ID:NM_025605%5D) | [BC020142](http://www.ebi.ac.uk/cgi-bin/emblfetch?BC020142) | -- |
| [M300008474](http://omad.operon.com/mouseV3/transcript.php?what=M300008474) | [D10Jhu81e](http://www.informatics.jax.org/searches/accession_report.cgi?id=MGI%3A1351861) | [NM_138601](http://srs.sanger.ac.uk/srsbin/cgi-bin/wgetz?-e+%5BREFSEQ-ID:NM_138601%5D) | [AB041855](http://www.ebi.ac.uk/cgi-bin/emblfetch?AB041855) | -- |
| [M200000096](http://omad.operon.com/mouseV3/transcript.php?what=M200000096) | [B3Gat3](http://www.informatics.jax.org/searches/accession_report.cgi?id=MGI%3A1919977) | [NM_024256](http://srs.sanger.ac.uk/srsbin/cgi-bin/wgetz?-e+%5BREFSEQ-ID:NM_024256%5D) | [BC002103](http://www.ebi.ac.uk/cgi-bin/emblfetch?BC002103) | GALACTOSYLGALACTOSYLXYLOSYLPROTEIN 3-BETA-GLUCURONOSYLTRANSFERASE 3 (EC 2.4.1.135) (BETA-1,3-GLUCURONYLTRANSFERASE 3) (GLUCURONOSYLTRANSFERASE-I) (GLCAT-I) (UDP-GLCUA:GAL BETA-1,3-GAL-R GLUCURONYLTRANSFERASE) (GLCUAT-I). |
| [M300006374](http://omad.operon.com/mouseV3/transcript.php?what=M300006374) | [Psmc2](http://www.informatics.jax.org/searches/accession_report.cgi?id=MGI%3A109555) | -- | [BC005462](http://www.ebi.ac.uk/cgi-bin/emblfetch?BC005462) | 26S PROTEASE REGULATORY SUBUNIT 7 (MSS1 PROTEIN). |
| [M300005124](http://omad.operon.com/mouseV3/transcript.php?what=M300005124) | [5730454B08Rik](http://www.informatics.jax.org/searches/accession_report.cgi?id=MGI%3A1917829) | [NM_144530](http://srs.sanger.ac.uk/srsbin/cgi-bin/wgetz?-e+%5BREFSEQ-ID:NM_144530%5D) | [BC005786](http://www.ebi.ac.uk/cgi-bin/emblfetch?BC005786) | -- |
| [M200000777](http://omad.operon.com/mouseV3/transcript.php?what=M200000777) | [G3bp-pending](http://www.informatics.jax.org/searches/accession_report.cgi?id=MGI%3A1351465) | [NM_013716](http://srs.sanger.ac.uk/srsbin/cgi-bin/wgetz?-e+%5BREFSEQ-ID:NM_013716%5D) | [AB001927](http://www.ebi.ac.uk/cgi-bin/emblfetch?AB001927) | RAS-GTPASE-ACTIVATING PROTEIN BINDING PROTEIN 1 (GAP SH3-DOMAIN BINDING PROTEIN 1) (G3BP-1). |
| [M200003749](http://omad.operon.com/mouseV3/transcript.php?what=M200003749) | -- | -- | -- | -- |
| [M300018559](http://omad.operon.com/mouseV3/transcript.php?what=M300018559) | -- | -- | -- | -- |
|  |  |  |  |  |
| **Female C57Bl6 6hr 200 cGy** | |  |  |  |
| [M200004687](http://omad.operon.com/mouseV3/transcript.php?what=M200004687) | [Dda3-pending](http://www.informatics.jax.org/searches/accession_report.cgi?id=MGI%3A1913099) | [NM_019976](http://srs.sanger.ac.uk/srsbin/cgi-bin/wgetz?-e+%5BREFSEQ-ID:NM_019976%5D) | [AK041835](http://www.ebi.ac.uk/cgi-bin/emblfetch?AK041835) | DIFFERENTIAL DISPLAY AND ACTIVATED BY P53; P53-REGULATED DDA3. |
| [M300020088](http://omad.operon.com/mouseV3/transcript.php?what=M300020088) | -- | -- | -- | -- |
| [M300004256](http://omad.operon.com/mouseV3/transcript.php?what=M300004256) | [Fth](http://www.informatics.jax.org/searches/accession_report.cgi?id=MGI%3A95588) | [NM_010239](http://srs.sanger.ac.uk/srsbin/cgi-bin/wgetz?-e+%5BREFSEQ-ID:NM_010239%5D) | [M24509](http://www.ebi.ac.uk/cgi-bin/emblfetch?M24509) | FERRITIN HEAVY CHAIN (FERRITIN H SUBUNIT). |
| [M300014099](http://omad.operon.com/mouseV3/transcript.php?what=M300014099) | [Actl](http://www.informatics.jax.org/searches/accession_report.cgi?id=MGI%3A109429) | [NM_013798](http://srs.sanger.ac.uk/srsbin/cgi-bin/wgetz?-e+%5BREFSEQ-ID:NM_013798%5D) | [AF195094](http://www.ebi.ac.uk/cgi-bin/emblfetch?AF195094) | ACTIN-LIKE. |
| [M300020371](http://omad.operon.com/mouseV3/transcript.php?what=M300020371) | -- | -- | -- | -- |
| [M200006851](http://omad.operon.com/mouseV3/transcript.php?what=M200006851) | -- | [NM_026467](http://srs.sanger.ac.uk/srsbin/cgi-bin/wgetz?-e+%5BREFSEQ-ID:NM_026467%5D) | -- | RIBOSOMAL PROTEIN S27-LIKE. |
| [M300015889](http://omad.operon.com/mouseV3/transcript.php?what=M300015889) | -- | -- | -- | -- |
| [M300019801](http://omad.operon.com/mouseV3/transcript.php?what=M300019801) | -- | -- | -- | -- |
| [M300018553](http://omad.operon.com/mouseV3/transcript.php?what=M300018553) | -- | -- | -- | -- |
| [M300021441](http://omad.operon.com/mouseV3/transcript.php?what=M300021441) | -- | -- | -- | -- |
| [M300015305](http://omad.operon.com/mouseV3/transcript.php?what=M300015305) | -- | -- | -- | -- |
| [M300019335](http://omad.operon.com/mouseV3/transcript.php?what=M300019335) | [Gapd](http://www.informatics.jax.org/searches/accession_report.cgi?id=MGI%3A95640) | [NM_008084](http://srs.sanger.ac.uk/srsbin/cgi-bin/wgetz?-e+%5BREFSEQ-ID:NM_008084%5D) | [AK002273](http://www.ebi.ac.uk/cgi-bin/emblfetch?AK002273) | GLYCERALDEHYDE 3-PHOSPHATE DEHYDROGENASE (EC 1.2.1.12) (GAPDH). |
| [M300020777](http://omad.operon.com/mouseV3/transcript.php?what=M300020777) | -- | -- | -- | -- |
| [M200003258](http://omad.operon.com/mouseV3/transcript.php?what=M200003258) | [Cox8a](http://www.informatics.jax.org/searches/accession_report.cgi?id=MGI%3A105959) | [NM_007750](http://srs.sanger.ac.uk/srsbin/cgi-bin/wgetz?-e+%5BREFSEQ-ID:NM_007750%5D) | [U37721](http://www.ebi.ac.uk/cgi-bin/emblfetch?U37721) | CYTOCHROME C OXIDASE POLYPEPTIDE VIII-LIVER, MITOCHONDRIAL PRECURSOR (EC 1.9.3.1). |
| [M300014515](http://omad.operon.com/mouseV3/transcript.php?what=M300014515) | -- | -- | -- | -- |
| [M300018314](http://omad.operon.com/mouseV3/transcript.php?what=M300018314) | -- | -- | -- | -- |
| [M200001083](http://omad.operon.com/mouseV3/transcript.php?what=M200001083) | [Hspa9a](http://www.informatics.jax.org/searches/accession_report.cgi?id=MGI%3A96245) | [NM_010481](http://srs.sanger.ac.uk/srsbin/cgi-bin/wgetz?-e+%5BREFSEQ-ID:NM_010481%5D) | [AK002634](http://www.ebi.ac.uk/cgi-bin/emblfetch?AK002634) | STRESS-70 PROTEIN, MITOCHONDRIAL PRECURSOR (75 KDA GLUCOSE REGULATED PROTEIN) (GRP 75) (PEPTIDE-BINDING PROTEIN 74) (PBP74) (P66 MOT) (MORTALIN). |
| [M300018559](http://omad.operon.com/mouseV3/transcript.php?what=M300018559) | -- | -- | -- | -- |
| [M300012796](http://omad.operon.com/mouseV3/transcript.php?what=M300012796) | [Hmgn1](http://www.informatics.jax.org/searches/accession_report.cgi?id=MGI%3A96120) | [NM_008251](http://srs.sanger.ac.uk/srsbin/cgi-bin/wgetz?-e+%5BREFSEQ-ID:NM_008251%5D) | [X53476](http://www.ebi.ac.uk/cgi-bin/emblfetch?X53476) | NONHISTONE CHROMOSOMAL PROTEIN HMG-14 (HIGH-MOBILITY GROUP NUCLEOSOME BINDING DOMAIN 1). |
| [M200000777](http://omad.operon.com/mouseV3/transcript.php?what=M200000777) | [G3bp-pending](http://www.informatics.jax.org/searches/accession_report.cgi?id=MGI%3A1351465) | [NM_013716](http://srs.sanger.ac.uk/srsbin/cgi-bin/wgetz?-e+%5BREFSEQ-ID:NM_013716%5D) | [AB001927](http://www.ebi.ac.uk/cgi-bin/emblfetch?AB001927) | RAS-GTPASE-ACTIVATING PROTEIN BINDING PROTEIN 1 (GAP SH3-DOMAIN BINDING PROTEIN 1) (G3BP-1). |
| [M300021668](http://omad.operon.com/mouseV3/transcript.php?what=M300021668) | -- | -- | -- | -- |
| [M300002115](http://omad.operon.com/mouseV3/transcript.php?what=M300002115) | [Xpo1](http://www.informatics.jax.org/searches/accession_report.cgi?id=MGI%3A2144013) | [NM_134014](http://srs.sanger.ac.uk/srsbin/cgi-bin/wgetz?-e+%5BREFSEQ-ID:NM_134014%5D) | [BC025628](http://www.ebi.ac.uk/cgi-bin/emblfetch?BC025628) | EXPORTIN 1, CRM1 HOMOLOG; EXPRESSED SEQUENCE AA420417. |
| [M300017554](http://omad.operon.com/mouseV3/transcript.php?what=M300017554) | [4930415K17Rik](http://www.informatics.jax.org/searches/accession_report.cgi?id=MGI%3A1914643) | [NM_133687](http://srs.sanger.ac.uk/srsbin/cgi-bin/wgetz?-e+%5BREFSEQ-ID:NM_133687%5D) | [BC016207](http://www.ebi.ac.uk/cgi-bin/emblfetch?BC016207) | -- |
| [M300004265](http://omad.operon.com/mouseV3/transcript.php?what=M300004265) | [Ms4a1](http://www.informatics.jax.org/searches/accession_report.cgi?id=MGI%3A88321) | [NM_007641](http://srs.sanger.ac.uk/srsbin/cgi-bin/wgetz?-e+%5BREFSEQ-ID:NM_007641%5D) | [AK017903](http://www.ebi.ac.uk/cgi-bin/emblfetch?AK017903) | B-CELL SURFACE PROTEIN CD20 HOMOLOG (B-CELL DIFFERENTIATION ANTIGEN LY-44). |
| [M200001144](http://omad.operon.com/mouseV3/transcript.php?what=M200001144) | [Cd79b](http://www.informatics.jax.org/searches/accession_report.cgi?id=MGI%3A96431) | [NM_008339](http://srs.sanger.ac.uk/srsbin/cgi-bin/wgetz?-e+%5BREFSEQ-ID:NM_008339%5D) | [AF002279](http://www.ebi.ac.uk/cgi-bin/emblfetch?AF002279) | B-CELL ANTIGEN RECEPTOR COMPLEX ASSOCIATED PROTEIN BETA-CHAIN PRECURSOR (B-CELL-SPECIFIC GLYCOPROTEIN B29) (IMMUNOGLOBULIN- ASSOCIATED B29 PROTEIN) (IG-BETA) (CD79B). |
|  |  |  |  |  |
| **Female C57Bl6 6hr 1000 cGy** | |  |  |  |
| [M200004687](http://omad.operon.com/mouseV3/transcript.php?what=M200004687) | [Dda3-pending](http://www.informatics.jax.org/searches/accession_report.cgi?id=MGI%3A1913099) | [NM_019976](http://srs.sanger.ac.uk/srsbin/cgi-bin/wgetz?-e+%5BREFSEQ-ID:NM_019976%5D) | [AK041835](http://www.ebi.ac.uk/cgi-bin/emblfetch?AK041835) | DIFFERENTIAL DISPLAY AND ACTIVATED BY P53; P53-REGULATED DDA3. |
| [M300008077](http://omad.operon.com/mouseV3/transcript.php?what=M300008077) | [Ei24](http://www.informatics.jax.org/searches/accession_report.cgi?id=MGI%3A108090) | [NM_007915](http://srs.sanger.ac.uk/srsbin/cgi-bin/wgetz?-e+%5BREFSEQ-ID:NM_007915%5D) | [U41751](http://www.ebi.ac.uk/cgi-bin/emblfetch?U41751) | ETOPOSIDE-INDUCED PROTEIN 2.4. |
| [M300011848](http://omad.operon.com/mouseV3/transcript.php?what=M300011848) | -- | [NM_173445](http://srs.sanger.ac.uk/srsbin/cgi-bin/wgetz?-e+%5BREFSEQ-ID:NM_173445%5D) | -- | -- |
| [M300020371](http://omad.operon.com/mouseV3/transcript.php?what=M300020371) | -- | -- | -- | -- |
| [M300019852](http://omad.operon.com/mouseV3/transcript.php?what=M300019852) | -- | -- | -- | -- |
| [M300019400](http://omad.operon.com/mouseV3/transcript.php?what=M300019400) | -- | -- | -- | -- |
| [M300019801](http://omad.operon.com/mouseV3/transcript.php?what=M300019801) | -- | -- | -- | -- |
| [M300014889](http://omad.operon.com/mouseV3/transcript.php?what=M300014889) | [Gapd](http://www.informatics.jax.org/searches/accession_report.cgi?id=MGI%3A95640) | [NM_008084](http://srs.sanger.ac.uk/srsbin/cgi-bin/wgetz?-e+%5BREFSEQ-ID:NM_008084%5D) | [AK002273](http://www.ebi.ac.uk/cgi-bin/emblfetch?AK002273) | GLYCERALDEHYDE 3-PHOSPHATE DEHYDROGENASE (EC 1.2.1.12) (GAPDH). |
| [M300000465](http://omad.operon.com/mouseV3/transcript.php?what=M300000465) | [2610301D06Rik](http://www.informatics.jax.org/searches/accession_report.cgi?id=MGI%3A1914410) | [NM_026007](http://srs.sanger.ac.uk/srsbin/cgi-bin/wgetz?-e+%5BREFSEQ-ID:NM_026007%5D) | [AK014277](http://www.ebi.ac.uk/cgi-bin/emblfetch?AK014277) | ELONGATION FACTOR 1-GAMMA (EF-1-GAMMA) (EEF-1B GAMMA). |
| [M300019589](http://omad.operon.com/mouseV3/transcript.php?what=M300019589) | -- | -- | -- | -- |
| [M300012879](http://omad.operon.com/mouseV3/transcript.php?what=M300012879) | -- | -- | [AK007389](http://www.ebi.ac.uk/cgi-bin/emblfetch?AK007389) | SMALL NUCLEAR RIBONUCLEOPROTEIN SM D2 (SNRNP CORE PROTEIN D2) (SM-D2). |
| [M300006168](http://omad.operon.com/mouseV3/transcript.php?what=M300006168) | -- | [NM_177045](http://srs.sanger.ac.uk/srsbin/cgi-bin/wgetz?-e+%5BREFSEQ-ID:NM_177045%5D) | -- | -- |
| [M300002970](http://omad.operon.com/mouseV3/transcript.php?what=M300002970) | [5730420B22Rik](http://www.informatics.jax.org/searches/accession_report.cgi?id=MGI%3A1917811) | [NM_172597](http://srs.sanger.ac.uk/srsbin/cgi-bin/wgetz?-e+%5BREFSEQ-ID:NM_172597%5D) | [AK017582](http://www.ebi.ac.uk/cgi-bin/emblfetch?AK017582) | -- |
| [M200009547](http://omad.operon.com/mouseV3/transcript.php?what=M200009547) | [Mybbp1a](http://www.informatics.jax.org/searches/accession_report.cgi?id=MGI%3A106181) | [NM_016776](http://srs.sanger.ac.uk/srsbin/cgi-bin/wgetz?-e+%5BREFSEQ-ID:NM_016776%5D) | [U63648](http://www.ebi.ac.uk/cgi-bin/emblfetch?U63648) | MYB BINDING PROTEIN (P160) 1A; NUCLEAR PROTEIN P160. |
| [M300021668](http://omad.operon.com/mouseV3/transcript.php?what=M300021668) | -- | -- | -- | -- |
| [M300011495](http://omad.operon.com/mouseV3/transcript.php?what=M300011495) | -- | -- | [BG088667](http://www.ebi.ac.uk/cgi-bin/emblfetch?BG088667) | SESTRIN 1 (P53-REGULATED PROTEIN PA26). |
| [M300017752](http://omad.operon.com/mouseV3/transcript.php?what=M300017752) | -- | -- | [AF516285](http://www.ebi.ac.uk/cgi-bin/emblfetch?AF516285) | ANTI-VIPASE LIGHT CHAIN VARIABLE REGION (FRAGMENT). |
| [M300007254](http://omad.operon.com/mouseV3/transcript.php?what=M300007254) | -- | [NM_172900](http://srs.sanger.ac.uk/srsbin/cgi-bin/wgetz?-e+%5BREFSEQ-ID:NM_172900%5D) | -- | -- |
| [M200006566](http://omad.operon.com/mouseV3/transcript.php?what=M200006566) | [Gga2](http://www.informatics.jax.org/searches/accession_report.cgi?id=MGI%3A1921355) | -- | [AK004632](http://www.ebi.ac.uk/cgi-bin/emblfetch?AK004632) | -- |
| [M200006174](http://omad.operon.com/mouseV3/transcript.php?what=M200006174) | [0610039P13Rik](http://www.informatics.jax.org/searches/accession_report.cgi?id=MGI%3A1921346) | [NM_028752](http://srs.sanger.ac.uk/srsbin/cgi-bin/wgetz?-e+%5BREFSEQ-ID:NM_028752%5D) | [BC021548](http://www.ebi.ac.uk/cgi-bin/emblfetch?BC021548) | -- |
| [M200000312](http://omad.operon.com/mouseV3/transcript.php?what=M200000312) | [Ly6d](http://www.informatics.jax.org/searches/accession_report.cgi?id=MGI%3A96881) | [NM_010742](http://srs.sanger.ac.uk/srsbin/cgi-bin/wgetz?-e+%5BREFSEQ-ID:NM_010742%5D) | [L40419](http://www.ebi.ac.uk/cgi-bin/emblfetch?L40419) | LYMPHOCYTE ANTIGEN LY-6D PRECURSOR (THYMOCYTE B CELL ANTIGEN) (THB). |
| [M200000320](http://omad.operon.com/mouseV3/transcript.php?what=M200000320) | [Pou2af1](http://www.informatics.jax.org/searches/accession_report.cgi?id=MGI%3A105086) | [NM_011136](http://srs.sanger.ac.uk/srsbin/cgi-bin/wgetz?-e+%5BREFSEQ-ID:NM_011136%5D) | [U43788](http://www.ebi.ac.uk/cgi-bin/emblfetch?U43788) | POU DOMAIN CLASS 2, ASSOCIATING FACTOR 1 (B-CELL-SPECIFIC COACTIVATOR OBF-1) (OCT BINDING FACTOR 1) (BOB-1) (BOB1) (OCA-B). |
| [M200002822](http://omad.operon.com/mouseV3/transcript.php?what=M200002822) | [Blnk](http://www.informatics.jax.org/searches/accession_report.cgi?id=MGI%3A96878) | [NM_008528](http://srs.sanger.ac.uk/srsbin/cgi-bin/wgetz?-e+%5BREFSEQ-ID:NM_008528%5D) | [AJ298054](http://www.ebi.ac.uk/cgi-bin/emblfetch?AJ298054) | B-CELL LINKER; LYMPHOCYTE ANTIGEN 57. |
| [M200001144](http://omad.operon.com/mouseV3/transcript.php?what=M200001144) | [Cd79b](http://www.informatics.jax.org/searches/accession_report.cgi?id=MGI%3A96431) | [NM_008339](http://srs.sanger.ac.uk/srsbin/cgi-bin/wgetz?-e+%5BREFSEQ-ID:NM_008339%5D) | [AF002279](http://www.ebi.ac.uk/cgi-bin/emblfetch?AF002279) | B-CELL ANTIGEN RECEPTOR COMPLEX ASSOCIATED PROTEIN BETA-CHAIN PRECURSOR (B-CELL-SPECIFIC GLYCOPROTEIN B29) (IMMUNOGLOBULIN- ASSOCIATED B29 PROTEIN) (IG-BETA) (CD79B). |
| [M200009317](http://omad.operon.com/mouseV3/transcript.php?what=M200009317) | [Scd1](http://www.informatics.jax.org/searches/accession_report.cgi?id=MGI%3A98239) | [NM_009127](http://srs.sanger.ac.uk/srsbin/cgi-bin/wgetz?-e+%5BREFSEQ-ID:NM_009127%5D) | [BC007474](http://www.ebi.ac.uk/cgi-bin/emblfetch?BC007474) | ACYL-COA DESATURASE 1 (EC 1.14.19.1) (STEAROYL-COA DESATURASE 1) (FATTY ACID DESATURASE 1) (DELTA(9)-DESATURASE 1). |
|  |  |  |  |  |
| **Female C57Bl6 24hr 50 cGy** | |  |  |  |
| [M300005062](http://omad.operon.com/mouseV3/transcript.php?what=M300005062) | [BC027756](http://www.informatics.jax.org/searches/accession_report.cgi?id=MGI%3A2384876) | [NM_145991](http://srs.sanger.ac.uk/srsbin/cgi-bin/wgetz?-e+%5BREFSEQ-ID:NM_145991%5D) | [AK080861](http://www.ebi.ac.uk/cgi-bin/emblfetch?AK080861) | -- |
| [M200005746](http://omad.operon.com/mouseV3/transcript.php?what=M200005746) | [1110020J08Rik](http://www.informatics.jax.org/searches/accession_report.cgi?id=MGI%3A1913419) | [NM_025394](http://srs.sanger.ac.uk/srsbin/cgi-bin/wgetz?-e+%5BREFSEQ-ID:NM_025394%5D) | [AK003864](http://www.ebi.ac.uk/cgi-bin/emblfetch?AK003864) | -- |
| [M200003036](http://omad.operon.com/mouseV3/transcript.php?what=M200003036) | [Nprl2-pending](http://www.informatics.jax.org/searches/accession_report.cgi?id=MGI%3A1914482) | [NM_018879](http://srs.sanger.ac.uk/srsbin/cgi-bin/wgetz?-e+%5BREFSEQ-ID:NM_018879%5D) | [BC026548](http://www.ebi.ac.uk/cgi-bin/emblfetch?BC026548) | G21 PROTEIN. |
| [M200004472](http://omad.operon.com/mouseV3/transcript.php?what=M200004472) | [Slc25a1](http://www.informatics.jax.org/searches/accession_report.cgi?id=MGI%3A1345283) | [NM_153150](http://srs.sanger.ac.uk/srsbin/cgi-bin/wgetz?-e+%5BREFSEQ-ID:NM_153150%5D) | [BC037087](http://www.ebi.ac.uk/cgi-bin/emblfetch?BC037087) | SOLUTE CARRIER FAMILY 25, MEMBER 1; DIGEORGE SYNDROME GENE J; SOLUTE CARRIER FAMILY 25 (MITOCHONDRIAL CARRIER; CITRATE TRANSPORTER) MEMBER 1; TRICARBOXYLATE TRANSPORT PROTEIN PRECURSOR. |
| [M200006750](http://omad.operon.com/mouseV3/transcript.php?what=M200006750) | [2410104I19Rik](http://www.informatics.jax.org/searches/accession_report.cgi?id=MGI%3A1915209) | [NM_133691](http://srs.sanger.ac.uk/srsbin/cgi-bin/wgetz?-e+%5BREFSEQ-ID:NM_133691%5D) | [BC010601](http://www.ebi.ac.uk/cgi-bin/emblfetch?BC010601) | -- |
| [M200009777](http://omad.operon.com/mouseV3/transcript.php?what=M200009777) | [Aco2](http://www.informatics.jax.org/searches/accession_report.cgi?id=MGI%3A87880) | [NM_080633](http://srs.sanger.ac.uk/srsbin/cgi-bin/wgetz?-e+%5BREFSEQ-ID:NM_080633%5D) | [BC004645](http://www.ebi.ac.uk/cgi-bin/emblfetch?BC004645) | ACONITASE 2, MITOCHONDRIAL. |
| [M200007587](http://omad.operon.com/mouseV3/transcript.php?what=M200007587) | [E130307M08Rik](http://www.informatics.jax.org/searches/accession_report.cgi?id=MGI%3A1915297) | [NM_026530](http://srs.sanger.ac.uk/srsbin/cgi-bin/wgetz?-e+%5BREFSEQ-ID:NM_026530%5D) | [BC017625](http://www.ebi.ac.uk/cgi-bin/emblfetch?BC017625) | -- |
| [M200002043](http://omad.operon.com/mouseV3/transcript.php?what=M200002043) | [Mcmd6](http://www.informatics.jax.org/searches/accession_report.cgi?id=MGI%3A1298227) | [NM_008567](http://srs.sanger.ac.uk/srsbin/cgi-bin/wgetz?-e+%5BREFSEQ-ID:NM_008567%5D) | [D86726](http://www.ebi.ac.uk/cgi-bin/emblfetch?D86726) | DNA REPLICATION LICENSING FACTOR MCM6 (MIS5 HOMOLOG). |
| [M200005598](http://omad.operon.com/mouseV3/transcript.php?what=M200005598) | [Cdk9](http://www.informatics.jax.org/searches/accession_report.cgi?id=MGI%3A1328368) | [NM_130860](http://srs.sanger.ac.uk/srsbin/cgi-bin/wgetz?-e+%5BREFSEQ-ID:NM_130860%5D) | [AF327431](http://www.ebi.ac.uk/cgi-bin/emblfetch?AF327431) | CYCLIN-DEPENDENT KINASE 9. |
| [M200006108](http://omad.operon.com/mouseV3/transcript.php?what=M200006108) | [Coro1b](http://www.informatics.jax.org/searches/accession_report.cgi?id=MGI%3A1345963) | [NM_011778](http://srs.sanger.ac.uk/srsbin/cgi-bin/wgetz?-e+%5BREFSEQ-ID:NM_011778%5D) | [AK008947](http://www.ebi.ac.uk/cgi-bin/emblfetch?AK008947) | CORONIN 1B (CORONIN 2). |
| [M300012497](http://omad.operon.com/mouseV3/transcript.php?what=M300012497) | [Rbms2](http://www.informatics.jax.org/searches/accession_report.cgi?id=MGI%3A1861776) | [NM_019711](http://srs.sanger.ac.uk/srsbin/cgi-bin/wgetz?-e+%5BREFSEQ-ID:NM_019711%5D) | [AK054482](http://www.ebi.ac.uk/cgi-bin/emblfetch?AK054482) | RNA BINDING MOTIF, SINGLE STRANDED INTERACTING PROTEIN 2; SCR3. |
| [M200003074](http://omad.operon.com/mouseV3/transcript.php?what=M200003074) | [Psmd3](http://www.informatics.jax.org/searches/accession_report.cgi?id=MGI%3A98858) | [NM_009439](http://srs.sanger.ac.uk/srsbin/cgi-bin/wgetz?-e+%5BREFSEQ-ID:NM_009439%5D) | [BC003197](http://www.ebi.ac.uk/cgi-bin/emblfetch?BC003197) | 26S PROTEASOME NON-ATPASE REGULATORY SUBUNIT 3 (26S PROTEASOME REGULATORY SUBUNIT S3) (PROTEASOME SUBUNIT P58) (TRANSPLANTATION ANTIGEN P91A) (TUM-P91A ANTIGEN). |
| [M300013135](http://omad.operon.com/mouseV3/transcript.php?what=M300013135) | -- | -- | [BC034540](http://www.ebi.ac.uk/cgi-bin/emblfetch?BC034540) | -- |
| [M300019447](http://omad.operon.com/mouseV3/transcript.php?what=M300019447) | -- | -- | [BC027368](http://www.ebi.ac.uk/cgi-bin/emblfetch?BC027368) | -- |
| [M200009417](http://omad.operon.com/mouseV3/transcript.php?what=M200009417) | [Mt2](http://www.informatics.jax.org/searches/accession_report.cgi?id=MGI%3A97172) | -- | [K02236](http://www.ebi.ac.uk/cgi-bin/emblfetch?K02236) | METALLOTHIONEIN-II (MT-II). |
| [M300021033](http://omad.operon.com/mouseV3/transcript.php?what=M300021033) | [Lgals3](http://www.informatics.jax.org/searches/accession_report.cgi?id=MGI%3A96778) | -- | [X16074](http://www.ebi.ac.uk/cgi-bin/emblfetch?X16074) | GALECTIN-3 (GALACTOSE-SPECIFIC LECTIN 3) (MAC-2 ANTIGEN) (IGE-BINDING PROTEIN) (35 KDA LECTIN) (CARBOHYDRATE BINDING PROTEIN 35) (CBP 35) (LAMININ-BINDING PROTEIN) (LECTIN L-29) (L-34 GALACTOSIDE-BINDING LECTIN). |
| [M300004485](http://omad.operon.com/mouseV3/transcript.php?what=M300004485) | [P4hb](http://www.informatics.jax.org/searches/accession_report.cgi?id=MGI%3A97464) | -- | [J05185](http://www.ebi.ac.uk/cgi-bin/emblfetch?J05185) | PROTEIN DISULFIDE ISOMERASE PRECURSOR (PDI) (EC 5.3.4.1) (PROLYL 4- HYDROXYLASE BETA SUBUNIT) (CELLULAR THYROID HORMONE BINDING PROTEIN) (P55) (ERP59). |
| [M200012720](http://omad.operon.com/mouseV3/transcript.php?what=M200012720) | -- | -- | [BC008093](http://www.ebi.ac.uk/cgi-bin/emblfetch?BC008093) | EUKARYOTIC TRANSLATION INITIATION FACTOR 5A (EIF-5A) (EIF-4D) (REV- BINDING FACTOR). |
| [M200006860](http://omad.operon.com/mouseV3/transcript.php?what=M200006860) | -- | [NM_010312](http://srs.sanger.ac.uk/srsbin/cgi-bin/wgetz?-e+%5BREFSEQ-ID:NM_010312%5D) | [U38505](http://www.ebi.ac.uk/cgi-bin/emblfetch?U38505) | GUANINE NUCLEOTIDE-BINDING PROTEIN G(I)/G(S)/G(T) BETA SUBUNIT 2 (TRANSDUCIN BETA CHAIN 2) (G PROTEIN BETA 2 SUBUNIT). |
| [M300011574](http://omad.operon.com/mouseV3/transcript.php?what=M300011574) | -- | -- | -- | -- |
| [M300015461](http://omad.operon.com/mouseV3/transcript.php?what=M300015461) | -- | -- | -- | -- |
| [M300021713](http://omad.operon.com/mouseV3/transcript.php?what=M300021713) | -- | -- | -- | -- |
| [M200009655](http://omad.operon.com/mouseV3/transcript.php?what=M200009655) | [Cct6a](http://www.informatics.jax.org/searches/accession_report.cgi?id=MGI%3A107943) | [NM_009838](http://srs.sanger.ac.uk/srsbin/cgi-bin/wgetz?-e+%5BREFSEQ-ID:NM_009838%5D) | [AB022159](http://www.ebi.ac.uk/cgi-bin/emblfetch?AB022159) | T-COMPLEX PROTEIN 1, ZETA SUBUNIT (TCP-1-ZETA) (CCT-ZETA) (CCT-ZETA- 1). |
| [M300004979](http://omad.operon.com/mouseV3/transcript.php?what=M300004979) | [Fn1](http://www.informatics.jax.org/searches/accession_report.cgi?id=MGI%3A95566) | -- | [BC004724](http://www.ebi.ac.uk/cgi-bin/emblfetch?BC004724) | -- |
| [M200014015](http://omad.operon.com/mouseV3/transcript.php?what=M200014015) | [Lgals1](http://www.informatics.jax.org/searches/accession_report.cgi?id=MGI%3A96777) | [NM_008495](http://srs.sanger.ac.uk/srsbin/cgi-bin/wgetz?-e+%5BREFSEQ-ID:NM_008495%5D) | [AK004298](http://www.ebi.ac.uk/cgi-bin/emblfetch?AK004298) | GALECTIN-1 (BETA-GALACTOSIDE-BINDING LECTIN L-14-I) (LACTOSE-BINDING LECTIN 1) (S-LAC LECTIN 1) (GALAPTIN) (14 KDA LECTIN). |
|  |  |  |  |  |
| **Female C57Bl6 24hr 200 cGy** | |  |  |  |
| [M300010249](http://omad.operon.com/mouseV3/transcript.php?what=M300010249) | [Txk](http://www.informatics.jax.org/searches/accession_report.cgi?id=MGI%3A102960) | [NM_013698](http://srs.sanger.ac.uk/srsbin/cgi-bin/wgetz?-e+%5BREFSEQ-ID:NM_013698%5D) | [L35268](http://www.ebi.ac.uk/cgi-bin/emblfetch?L35268) | TYROSINE-PROTEIN KINASE TXK (EC 2.7.1.112) (PTK-RL-18) (RESTING LYMPHOCYTE KINASE). |
| [M300010028](http://omad.operon.com/mouseV3/transcript.php?what=M300010028) | -- | -- | [BC026557](http://www.ebi.ac.uk/cgi-bin/emblfetch?BC026557) | SIMILAR TO PTD015 PROTEIN. |
| [M200009777](http://omad.operon.com/mouseV3/transcript.php?what=M200009777) | [Aco2](http://www.informatics.jax.org/searches/accession_report.cgi?id=MGI%3A87880) | [NM_080633](http://srs.sanger.ac.uk/srsbin/cgi-bin/wgetz?-e+%5BREFSEQ-ID:NM_080633%5D) | [BC004645](http://www.ebi.ac.uk/cgi-bin/emblfetch?BC004645) | ACONITASE 2, MITOCHONDRIAL. |
| [M200005598](http://omad.operon.com/mouseV3/transcript.php?what=M200005598) | [Cdk9](http://www.informatics.jax.org/searches/accession_report.cgi?id=MGI%3A1328368) | [NM_130860](http://srs.sanger.ac.uk/srsbin/cgi-bin/wgetz?-e+%5BREFSEQ-ID:NM_130860%5D) | [AF327431](http://www.ebi.ac.uk/cgi-bin/emblfetch?AF327431) | CYCLIN-DEPENDENT KINASE 9. |
| [M200000327](http://omad.operon.com/mouseV3/transcript.php?what=M200000327) | [Cct7](http://www.informatics.jax.org/searches/accession_report.cgi?id=MGI%3A107184) | [NM_007638](http://srs.sanger.ac.uk/srsbin/cgi-bin/wgetz?-e+%5BREFSEQ-ID:NM_007638%5D) | [AB022160](http://www.ebi.ac.uk/cgi-bin/emblfetch?AB022160) | T-COMPLEX PROTEIN 1, ETA SUBUNIT (TCP-1-ETA) (CCT-ETA). |
| [M200003578](http://omad.operon.com/mouseV3/transcript.php?what=M200003578) | [Bpnt1](http://www.informatics.jax.org/searches/accession_report.cgi?id=MGI%3A1338800) | [NM_011794](http://srs.sanger.ac.uk/srsbin/cgi-bin/wgetz?-e+%5BREFSEQ-ID:NM_011794%5D) | [AF125043](http://www.ebi.ac.uk/cgi-bin/emblfetch?AF125043) | BISPHOSPHATE 3'-NUCLEOTIDASE 1. |
| [M200002251](http://omad.operon.com/mouseV3/transcript.php?what=M200002251) | [Akr1b8](http://www.informatics.jax.org/searches/accession_report.cgi?id=MGI%3A107673) | [NM_008012](http://srs.sanger.ac.uk/srsbin/cgi-bin/wgetz?-e+%5BREFSEQ-ID:NM_008012%5D) | [U04204](http://www.ebi.ac.uk/cgi-bin/emblfetch?U04204) | ALDOSE REDUCTASE-RELATED PROTEIN 1 (EC 1.1.1.21) (AR) (ALDEHYDE REDUCTASE) (VAS DEFERENS ANDROGEN-DEPENDENT PROTEIN) (MVDP) (ALDO-KETO REDUCTASE FAMILY 1 MEMBER B7). |
| [M200012683](http://omad.operon.com/mouseV3/transcript.php?what=M200012683) | [Acat2](http://www.informatics.jax.org/searches/accession_report.cgi?id=MGI%3A87871) | -- | [BC012496](http://www.ebi.ac.uk/cgi-bin/emblfetch?BC012496) | T-COMPLEX PROTEIN (TCP-1X) (FRAGMENT). |
| [M300002824](http://omad.operon.com/mouseV3/transcript.php?what=M300002824) | [Hnrpk](http://www.informatics.jax.org/searches/accession_report.cgi?id=MGI%3A99894) | [NM_025279](http://srs.sanger.ac.uk/srsbin/cgi-bin/wgetz?-e+%5BREFSEQ-ID:NM_025279%5D) | [BC006694](http://www.ebi.ac.uk/cgi-bin/emblfetch?BC006694) | HETEROGENEOUS NUCLEAR RIBONUCLEOPROTEIN K (HNRNP K) (65 KDA PHOSPHOPROTEIN). |
| [M200007603](http://omad.operon.com/mouseV3/transcript.php?what=M200007603) | [0610009O03Rik](http://www.informatics.jax.org/searches/accession_report.cgi?id=MGI%3A1915544) | [NM_026660](http://srs.sanger.ac.uk/srsbin/cgi-bin/wgetz?-e+%5BREFSEQ-ID:NM_026660%5D) | [AK089055](http://www.ebi.ac.uk/cgi-bin/emblfetch?AK089055) | -- |
| [M200006373](http://omad.operon.com/mouseV3/transcript.php?what=M200006373) | [Nars](http://www.informatics.jax.org/searches/accession_report.cgi?id=MGI%3A1917473) | -- | [AK013880](http://www.ebi.ac.uk/cgi-bin/emblfetch?AK013880) | -- |
| [M200002442](http://omad.operon.com/mouseV3/transcript.php?what=M200002442) | [Cdk4](http://www.informatics.jax.org/searches/accession_report.cgi?id=MGI%3A88357) | [NM_009870](http://srs.sanger.ac.uk/srsbin/cgi-bin/wgetz?-e+%5BREFSEQ-ID:NM_009870%5D) | [X65069](http://www.ebi.ac.uk/cgi-bin/emblfetch?X65069) | CELL DIVISION PROTEIN KINASE 4 (EC 2.7.1.-) (CYCLIN-DEPENDENT KINASE 4) (PSK-J3) (CRK3). |
| [M200006712](http://omad.operon.com/mouseV3/transcript.php?what=M200006712) | [Shmt2](http://www.informatics.jax.org/searches/accession_report.cgi?id=MGI%3A1277989) | [NM_028230](http://srs.sanger.ac.uk/srsbin/cgi-bin/wgetz?-e+%5BREFSEQ-ID:NM_028230%5D) | [BC004825](http://www.ebi.ac.uk/cgi-bin/emblfetch?BC004825) | -- |
| [M200002501](http://omad.operon.com/mouseV3/transcript.php?what=M200002501) | [Lrp1](http://www.informatics.jax.org/searches/accession_report.cgi?id=MGI%3A96828) | [NM_008512](http://srs.sanger.ac.uk/srsbin/cgi-bin/wgetz?-e+%5BREFSEQ-ID:NM_008512%5D) | [AF367720](http://www.ebi.ac.uk/cgi-bin/emblfetch?AF367720) | LOW DENSITY LIPOPROTEIN RECEPTOR-RELATED PROTEIN 1; LOW DENSITY LIPOPROTEIN RECEPTOR RELATED PROTEIN; LOW DENSITY LIPOPROTEIN RECEPTOR RELATED PROTEIN 1. |
| [M200006860](http://omad.operon.com/mouseV3/transcript.php?what=M200006860) | -- | [NM_010312](http://srs.sanger.ac.uk/srsbin/cgi-bin/wgetz?-e+%5BREFSEQ-ID:NM_010312%5D) | [U38505](http://www.ebi.ac.uk/cgi-bin/emblfetch?U38505) | GUANINE NUCLEOTIDE-BINDING PROTEIN G(I)/G(S)/G(T) BETA SUBUNIT 2 (TRANSDUCIN BETA CHAIN 2) (G PROTEIN BETA 2 SUBUNIT). |
| [M300004485](http://omad.operon.com/mouseV3/transcript.php?what=M300004485) | [P4hb](http://www.informatics.jax.org/searches/accession_report.cgi?id=MGI%3A97464) | -- | [J05185](http://www.ebi.ac.uk/cgi-bin/emblfetch?J05185) | PROTEIN DISULFIDE ISOMERASE PRECURSOR (PDI) (EC 5.3.4.1) (PROLYL 4- HYDROXYLASE BETA SUBUNIT) (CELLULAR THYROID HORMONE BINDING PROTEIN) (P55) (ERP59). |
| [M200012927](http://omad.operon.com/mouseV3/transcript.php?what=M200012927) | [Angptl2](http://www.informatics.jax.org/searches/accession_report.cgi?id=MGI%3A1347002) | [NM_011923](http://srs.sanger.ac.uk/srsbin/cgi-bin/wgetz?-e+%5BREFSEQ-ID:NM_011923%5D) | [AF125176](http://www.ebi.ac.uk/cgi-bin/emblfetch?AF125176) | ANGIOPOIETIN-RELATED PROTEIN 2 PRECURSOR (ANGIOPOIETIN-LIKE 2). |
| [M300011172](http://omad.operon.com/mouseV3/transcript.php?what=M300011172) | -- | -- | -- | -- |
| [M200002468](http://omad.operon.com/mouseV3/transcript.php?what=M200002468) | [Alad](http://www.informatics.jax.org/searches/accession_report.cgi?id=MGI%3A96853) | [NM_008525](http://srs.sanger.ac.uk/srsbin/cgi-bin/wgetz?-e+%5BREFSEQ-ID:NM_008525%5D) | [X13752](http://www.ebi.ac.uk/cgi-bin/emblfetch?X13752) | DELTA-AMINOLEVULINIC ACID DEHYDRATASE (EC 4.2.1.24) (PORPHOBILINOGEN SYNTHASE) (ALADH). |
| [M300004916](http://omad.operon.com/mouseV3/transcript.php?what=M300004916) | [Col3a1](http://www.informatics.jax.org/searches/accession_report.cgi?id=MGI%3A88453) | -- | [X57983](http://www.ebi.ac.uk/cgi-bin/emblfetch?X57983) | COLLAGEN ALPHA 1(III) CHAIN PRECURSOR. |
| [M200000033](http://omad.operon.com/mouseV3/transcript.php?what=M200000033) | [Idb3](http://www.informatics.jax.org/searches/accession_report.cgi?id=MGI%3A96398) | [NM_008321](http://srs.sanger.ac.uk/srsbin/cgi-bin/wgetz?-e+%5BREFSEQ-ID:NM_008321%5D) | [M60523](http://www.ebi.ac.uk/cgi-bin/emblfetch?M60523) | DNA-BINDING PROTEIN INHIBITOR ID-3 (ID-LIKE PROTEIN INHIBITOR HLH 462). |
| [M200003353](http://omad.operon.com/mouseV3/transcript.php?what=M200003353) | [Anxa1](http://www.informatics.jax.org/searches/accession_report.cgi?id=MGI%3A96819) | [NM_010730](http://srs.sanger.ac.uk/srsbin/cgi-bin/wgetz?-e+%5BREFSEQ-ID:NM_010730%5D) | [M24554](http://www.ebi.ac.uk/cgi-bin/emblfetch?M24554) | ANNEXIN I (LIPOCORTIN I) (CALPACTIN II) (CHROMOBINDIN 9) (P35) (PHOSPHOLIPASE A2 INHIBITORY PROTEIN). |
| [M200014015](http://omad.operon.com/mouseV3/transcript.php?what=M200014015) | [Lgals1](http://www.informatics.jax.org/searches/accession_report.cgi?id=MGI%3A96777) | [NM_008495](http://srs.sanger.ac.uk/srsbin/cgi-bin/wgetz?-e+%5BREFSEQ-ID:NM_008495%5D) | [AK004298](http://www.ebi.ac.uk/cgi-bin/emblfetch?AK004298) | GALECTIN-1 (BETA-GALACTOSIDE-BINDING LECTIN L-14-I) (LACTOSE-BINDING LECTIN 1) (S-LAC LECTIN 1) (GALAPTIN) (14 KDA LECTIN). |
| [M200000992](http://omad.operon.com/mouseV3/transcript.php?what=M200000992) | [Bgn](http://www.informatics.jax.org/searches/accession_report.cgi?id=MGI%3A88158) | [NM_007542](http://srs.sanger.ac.uk/srsbin/cgi-bin/wgetz?-e+%5BREFSEQ-ID:NM_007542%5D) | [Y11758](http://www.ebi.ac.uk/cgi-bin/emblfetch?Y11758) | BIGLYCAN PRECURSOR (BONE/CARTILAGE PROTEOGLYCAN I) (PG-S1). |
| [M200003310](http://omad.operon.com/mouseV3/transcript.php?what=M200003310) | [AU044919](http://www.informatics.jax.org/searches/accession_report.cgi?id=MGI%3A2144967) | -- | [BC010327](http://www.ebi.ac.uk/cgi-bin/emblfetch?BC010327) | IG GAMMA-2B CHAIN C REGION, MEMBRANE-BOUND FORM. |
|  |  |  |  |  |
| **Female C57Bl6 24hr 1000 cGy** | |  |  |  |
| [M300000233](http://omad.operon.com/mouseV3/transcript.php?what=M300000233) | [Capns1](http://www.informatics.jax.org/searches/accession_report.cgi?id=MGI%3A88266) | [NM_009795](http://srs.sanger.ac.uk/srsbin/cgi-bin/wgetz?-e+%5BREFSEQ-ID:NM_009795%5D) | [BC018352](http://www.ebi.ac.uk/cgi-bin/emblfetch?BC018352) | CALCIUM-DEPENDENT PROTEASE, SMALL SUBUNIT (CALPAIN REGULATORY SUBUNIT) (CALCIUM-ACTIVATED NEUTRAL PROTEINASE) (CANP). |
| [M300001059](http://omad.operon.com/mouseV3/transcript.php?what=M300001059) | [D0H8S2298E](http://www.informatics.jax.org/searches/accession_report.cgi?id=MGI%3A1337129) | -- | [BC024492](http://www.ebi.ac.uk/cgi-bin/emblfetch?BC024492) | REPRODUCTION 8 (DNA SEGMENT, HUMAN S2298E). |
| [M300013845](http://omad.operon.com/mouseV3/transcript.php?what=M300013845) | [Atpaf2](http://www.informatics.jax.org/searches/accession_report.cgi?id=MGI%3A2180561) | [NM_145427](http://srs.sanger.ac.uk/srsbin/cgi-bin/wgetz?-e+%5BREFSEQ-ID:NM_145427%5D) | [BC013607](http://www.ebi.ac.uk/cgi-bin/emblfetch?BC013607) | ATP SYNTHASE MITOCHONDRIAL F1 COMPLEX ASSEMBLY FACTOR 2. |
| [M300004022](http://omad.operon.com/mouseV3/transcript.php?what=M300004022) | [Ermelin-pending](http://www.informatics.jax.org/searches/accession_report.cgi?id=MGI%3A2147279) | [NM_139143](http://srs.sanger.ac.uk/srsbin/cgi-bin/wgetz?-e+%5BREFSEQ-ID:NM_139143%5D) | [AB071697](http://www.ebi.ac.uk/cgi-bin/emblfetch?AB071697) | ENDOPLASMIC RETICULUM MEMBRANE PROTEIN; EXPRESSED SEQUENCE AI853222. |
| [M200004159](http://omad.operon.com/mouseV3/transcript.php?what=M200004159) | [Nono](http://www.informatics.jax.org/searches/accession_report.cgi?id=MGI%3A1855692) | [NM_023144](http://srs.sanger.ac.uk/srsbin/cgi-bin/wgetz?-e+%5BREFSEQ-ID:NM_023144%5D) | [AK013444](http://www.ebi.ac.uk/cgi-bin/emblfetch?AK013444) | NON-POU-DOMAIN-CONTAINING, OCTAMER BINDING PROTEIN; NON-POU-DOMAIN-CONTAINING, OCTAMER-BINDING PROTEIN. |
| [M200003982](http://omad.operon.com/mouseV3/transcript.php?what=M200003982) | [Golga5](http://www.informatics.jax.org/searches/accession_report.cgi?id=MGI%3A1351475) | [NM_013747](http://srs.sanger.ac.uk/srsbin/cgi-bin/wgetz?-e+%5BREFSEQ-ID:NM_013747%5D) | [AF026274](http://www.ebi.ac.uk/cgi-bin/emblfetch?AF026274) | GOLGI AUTOANTIGEN, GOLGIN SUBFAMILY A, 5. |
| [M200000385](http://omad.operon.com/mouseV3/transcript.php?what=M200000385) | [Slc1a7](http://www.informatics.jax.org/searches/accession_report.cgi?id=MGI%3A105305) | [NM_009201](http://srs.sanger.ac.uk/srsbin/cgi-bin/wgetz?-e+%5BREFSEQ-ID:NM_009201%5D) | [D85044](http://www.ebi.ac.uk/cgi-bin/emblfetch?D85044) | NEUTRAL AMINO ACID TRANSPORTER B (INSULIN-ACTIVATED AMINO ACID TRANSPORTER) (ASC-LIKE NA(+) DEPENDENT NEUTRAL AMINO ACID TRANSPORTER ASCT2). |
| [M300006374](http://omad.operon.com/mouseV3/transcript.php?what=M300006374) | [Psmc2](http://www.informatics.jax.org/searches/accession_report.cgi?id=MGI%3A109555) | -- | [BC005462](http://www.ebi.ac.uk/cgi-bin/emblfetch?BC005462) | 26S PROTEASE REGULATORY SUBUNIT 7 (MSS1 PROTEIN). |
| [M200004383](http://omad.operon.com/mouseV3/transcript.php?what=M200004383) | [Cse1l](http://www.informatics.jax.org/searches/accession_report.cgi?id=MGI%3A1339951) | [NM_023565](http://srs.sanger.ac.uk/srsbin/cgi-bin/wgetz?-e+%5BREFSEQ-ID:NM_023565%5D) | [AF301152](http://www.ebi.ac.uk/cgi-bin/emblfetch?AF301152) | IMPORTIN-ALPHA RE-EXPORTER (CHROMOSOME SEGREGATION 1-LIKE PROTEIN) (CELLULAR APOPTOSIS SUSCEPTIBILITY PROTEIN). |
| [M200005955](http://omad.operon.com/mouseV3/transcript.php?what=M200005955) | [1810019E15Rik](http://www.informatics.jax.org/searches/accession_report.cgi?id=MGI%3A1914254) | -- | [AK007546](http://www.ebi.ac.uk/cgi-bin/emblfetch?AK007546) | -- |
| [M200005912](http://omad.operon.com/mouseV3/transcript.php?what=M200005912) | [Narg1](http://www.informatics.jax.org/searches/accession_report.cgi?id=MGI%3A1922088) | [NM_053089](http://srs.sanger.ac.uk/srsbin/cgi-bin/wgetz?-e+%5BREFSEQ-ID:NM_053089%5D) | [BC030167](http://www.ebi.ac.uk/cgi-bin/emblfetch?BC030167) | NMDA RECEPTOR-REGULATED GENE 1; N-TERMINAL ACEYLTRANSFERASE 1. |
| [M200001798](http://omad.operon.com/mouseV3/transcript.php?what=M200001798) | [Lbr](http://www.informatics.jax.org/searches/accession_report.cgi?id=MGI%3A2138281) | [NM_133815](http://srs.sanger.ac.uk/srsbin/cgi-bin/wgetz?-e+%5BREFSEQ-ID:NM_133815%5D) | [BC042522](http://www.ebi.ac.uk/cgi-bin/emblfetch?BC042522) | LAMIN B RECEPTOR; ICHTHYOSIS. |
| [M200015331](http://omad.operon.com/mouseV3/transcript.php?what=M200015331) | [AV278559](http://www.informatics.jax.org/searches/accession_report.cgi?id=MGI%3A2147677) | [NM_134152](http://srs.sanger.ac.uk/srsbin/cgi-bin/wgetz?-e+%5BREFSEQ-ID:NM_134152%5D) | [AB071194](http://www.ebi.ac.uk/cgi-bin/emblfetch?AB071194) | -- |
| [M300022323](http://omad.operon.com/mouseV3/transcript.php?what=M300022323) | -- | -- | -- | -- |
| [M300021610](http://omad.operon.com/mouseV3/transcript.php?what=M300021610) | -- | -- | -- | -- |
| [M300017722](http://omad.operon.com/mouseV3/transcript.php?what=M300017722) | -- | [NM_024266](http://srs.sanger.ac.uk/srsbin/cgi-bin/wgetz?-e+%5BREFSEQ-ID:NM_024266%5D) | [X62482](http://www.ebi.ac.uk/cgi-bin/emblfetch?X62482) | 40S RIBOSOMAL PROTEIN S25. |
| [M200003662](http://omad.operon.com/mouseV3/transcript.php?what=M200003662) | [Hprt](http://www.informatics.jax.org/searches/accession_report.cgi?id=MGI%3A96217) | [NM_013556](http://srs.sanger.ac.uk/srsbin/cgi-bin/wgetz?-e+%5BREFSEQ-ID:NM_013556%5D) | [K01514](http://www.ebi.ac.uk/cgi-bin/emblfetch?K01514) | HYPOXANTHINE-GUANINE PHOSPHORIBOSYLTRANSFERASE (EC 2.4.2.8) (HGPRT) (HGPRTASE) (HPRT B). |
| [M300004429](http://omad.operon.com/mouseV3/transcript.php?what=M300004429) | [Blnk](http://www.informatics.jax.org/searches/accession_report.cgi?id=MGI%3A96878) | [NM_008528](http://srs.sanger.ac.uk/srsbin/cgi-bin/wgetz?-e+%5BREFSEQ-ID:NM_008528%5D) | [AJ222814](http://www.ebi.ac.uk/cgi-bin/emblfetch?AJ222814) | B-CELL LINKER; LYMPHOCYTE ANTIGEN 57. |
| [M300018162](http://omad.operon.com/mouseV3/transcript.php?what=M300018162) | -- | -- | -- | -- |
| [M300013112](http://omad.operon.com/mouseV3/transcript.php?what=M300013112) | -- | -- | [J00595](http://www.ebi.ac.uk/cgi-bin/emblfetch?J00595) | IG LAMBDA-2 CHAIN C REGION. |
| [M300011693](http://omad.operon.com/mouseV3/transcript.php?what=M300011693) | -- | -- | -- | -- |
| [M300000425](http://omad.operon.com/mouseV3/transcript.php?what=M300000425) | [Rps11](http://www.informatics.jax.org/searches/accession_report.cgi?id=MGI%3A1351329) | [NM_013725](http://srs.sanger.ac.uk/srsbin/cgi-bin/wgetz?-e+%5BREFSEQ-ID:NM_013725%5D) | [AK005147](http://www.ebi.ac.uk/cgi-bin/emblfetch?AK005147) | 40S RIBOSOMAL PROTEIN S11. |
| [M300017758](http://omad.operon.com/mouseV3/transcript.php?what=M300017758) | -- | [NM_027015](http://srs.sanger.ac.uk/srsbin/cgi-bin/wgetz?-e+%5BREFSEQ-ID:NM_027015%5D) | -- | RIBOSOMAL PROTEIN S27. |
| [M300004265](http://omad.operon.com/mouseV3/transcript.php?what=M300004265) | [Ms4a1](http://www.informatics.jax.org/searches/accession_report.cgi?id=MGI%3A88321) | [NM_007641](http://srs.sanger.ac.uk/srsbin/cgi-bin/wgetz?-e+%5BREFSEQ-ID:NM_007641%5D) | [AK017903](http://www.ebi.ac.uk/cgi-bin/emblfetch?AK017903) | B-CELL SURFACE PROTEIN CD20 HOMOLOG (B-CELL DIFFERENTIATION ANTIGEN LY-44). |
| [M300020997](http://omad.operon.com/mouseV3/transcript.php?what=M300020997) | -- | -- | -- | -- |
|  |  |  |  |  |
| **Female C57Bl6 day 7 50 cGy** | |  |  |  |
| [M300007861](http://omad.operon.com/mouseV3/transcript.php?what=M300007861) | [Gypa](http://www.informatics.jax.org/searches/accession_report.cgi?id=MGI%3A95880) | [NM_010369](http://srs.sanger.ac.uk/srsbin/cgi-bin/wgetz?-e+%5BREFSEQ-ID:NM_010369%5D) | [M73815](http://www.ebi.ac.uk/cgi-bin/emblfetch?M73815) | GLYCOPHORIN. |
| [M200006628](http://omad.operon.com/mouseV3/transcript.php?what=M200006628) | [W64236](http://www.informatics.jax.org/searches/accession_report.cgi?id=MGI%3A2137870) | [NM_144805](http://srs.sanger.ac.uk/srsbin/cgi-bin/wgetz?-e+%5BREFSEQ-ID:NM_144805%5D) | [BC019416](http://www.ebi.ac.uk/cgi-bin/emblfetch?BC019416) | -- |
| [M300005566](http://omad.operon.com/mouseV3/transcript.php?what=M300005566) | [Capn3](http://www.informatics.jax.org/searches/accession_report.cgi?id=MGI%3A107437) | [NM_007601](http://srs.sanger.ac.uk/srsbin/cgi-bin/wgetz?-e+%5BREFSEQ-ID:NM_007601%5D) | [AF091998](http://www.ebi.ac.uk/cgi-bin/emblfetch?AF091998) | CALPAIN 3 LARGE SUBUNIT (EC 3.4.22.17) (CALPAIN L3) (CALPAIN P94, LARGE SUBUNIT) (CALCIUM-ACTIVATED NEUTRAL PROTEINASE 3) (CANP 3) (MUSCLE-SPECIFIC CALCIUM-ACTIVATED NEUTRAL PROTEASE 3 LARGE SUBUNIT). |
| [M200001376](http://omad.operon.com/mouseV3/transcript.php?what=M200001376) | [Gp5](http://www.informatics.jax.org/searches/accession_report.cgi?id=MGI%3A1096363) | [NM_008148](http://srs.sanger.ac.uk/srsbin/cgi-bin/wgetz?-e+%5BREFSEQ-ID:NM_008148%5D) | [Z69595](http://www.ebi.ac.uk/cgi-bin/emblfetch?Z69595) | PLATELET GLYCOPROTEIN V PRECURSOR (GPV) (CD42D). |
| [M200005863](http://omad.operon.com/mouseV3/transcript.php?what=M200005863) | [Nup210](http://www.informatics.jax.org/searches/accession_report.cgi?id=MGI%3A1859555) | [NM_018815](http://srs.sanger.ac.uk/srsbin/cgi-bin/wgetz?-e+%5BREFSEQ-ID:NM_018815%5D) | [AF113751](http://www.ebi.ac.uk/cgi-bin/emblfetch?AF113751) | NUCLEOPORIN 210; NUCLEAR PORE MEMBRANE GLYCOPROTEIN 210; NUCLEAR PORE MEMBRANE PROTEIN 210. |
| [M200007831](http://omad.operon.com/mouseV3/transcript.php?what=M200007831) | [4933407D05Rik](http://www.informatics.jax.org/searches/accession_report.cgi?id=MGI%3A1921320) | [NM_029748](http://srs.sanger.ac.uk/srsbin/cgi-bin/wgetz?-e+%5BREFSEQ-ID:NM_029748%5D) | [AK016715](http://www.ebi.ac.uk/cgi-bin/emblfetch?AK016715) | -- |
| [M200001259](http://omad.operon.com/mouseV3/transcript.php?what=M200001259) | [Cnih](http://www.informatics.jax.org/searches/accession_report.cgi?id=MGI%3A1277202) | [NM_009919](http://srs.sanger.ac.uk/srsbin/cgi-bin/wgetz?-e+%5BREFSEQ-ID:NM_009919%5D) | [AF022811](http://www.ebi.ac.uk/cgi-bin/emblfetch?AF022811) | CORNICHON HOMOLOG. |
| [M200000413](http://omad.operon.com/mouseV3/transcript.php?what=M200000413) | [Hdgf](http://www.informatics.jax.org/searches/accession_report.cgi?id=MGI%3A1194494) | [NM_008231](http://srs.sanger.ac.uk/srsbin/cgi-bin/wgetz?-e+%5BREFSEQ-ID:NM_008231%5D) | [BC021654](http://www.ebi.ac.uk/cgi-bin/emblfetch?BC021654) | HEPATOMA-DERIVED GROWTH FACTOR (HDGF). |
| [M200003736](http://omad.operon.com/mouseV3/transcript.php?what=M200003736) | [Prdx4](http://www.informatics.jax.org/searches/accession_report.cgi?id=MGI%3A1859815) | [NM_016764](http://srs.sanger.ac.uk/srsbin/cgi-bin/wgetz?-e+%5BREFSEQ-ID:NM_016764%5D) | [U96746](http://www.ebi.ac.uk/cgi-bin/emblfetch?U96746) | PEROXIREDOXIN 4 (EC 1.11.1.-) (PRX-IV) (THIOREDOXIN PEROXIDASE AO372) (THIOREDOXIN-DEPENDENT PEROXIDE REDUCTASE A0372) (ANTIOXIDANT ENZYME AOE372). |
| [M300003493](http://omad.operon.com/mouseV3/transcript.php?what=M300003493) | -- | -- | [BC028899](http://www.ebi.ac.uk/cgi-bin/emblfetch?BC028899) | PEPTIDYL-PROLYL CIS-TRANS ISOMERASE LIKE 2 (EC 5.2.1.8) (PPIASE) (ROTAMASE) (CYCLOPHILIN-60) (CYCLOPHILIN-LIKE PROTEIN CYP-60). |
| [M300020830](http://omad.operon.com/mouseV3/transcript.php?what=M300020830) | -- | -- | -- | -- |
| [M200004428](http://omad.operon.com/mouseV3/transcript.php?what=M200004428) | [0610016L08Rik](http://www.informatics.jax.org/searches/accession_report.cgi?id=MGI%3A1924141) | [NM_029787](http://srs.sanger.ac.uk/srsbin/cgi-bin/wgetz?-e+%5BREFSEQ-ID:NM_029787%5D) | [BC032013](http://www.ebi.ac.uk/cgi-bin/emblfetch?BC032013) | DIAPHORASE 1 (NADH). |
| [M200006257](http://omad.operon.com/mouseV3/transcript.php?what=M200006257) | [2610312E17Rik](http://www.informatics.jax.org/searches/accession_report.cgi?id=MGI%3A1917715) | [NM_027432](http://srs.sanger.ac.uk/srsbin/cgi-bin/wgetz?-e+%5BREFSEQ-ID:NM_027432%5D) | [AK050391](http://www.ebi.ac.uk/cgi-bin/emblfetch?AK050391) | -- |
| [M200009010](http://omad.operon.com/mouseV3/transcript.php?what=M200009010) | [AI840044](http://www.informatics.jax.org/searches/accession_report.cgi?id=MGI%3A2139806) | [NM_144895](http://srs.sanger.ac.uk/srsbin/cgi-bin/wgetz?-e+%5BREFSEQ-ID:NM_144895%5D) | [BC022921](http://www.ebi.ac.uk/cgi-bin/emblfetch?BC022921) | -- |
| [M300001264](http://omad.operon.com/mouseV3/transcript.php?what=M300001264) | [1810036I24Rik](http://www.informatics.jax.org/searches/accession_report.cgi?id=MGI%3A1914760) | [NM_026210](http://srs.sanger.ac.uk/srsbin/cgi-bin/wgetz?-e+%5BREFSEQ-ID:NM_026210%5D) | [AK077277](http://www.ebi.ac.uk/cgi-bin/emblfetch?AK077277) | -- |
| [M300013796](http://omad.operon.com/mouseV3/transcript.php?what=M300013796) | [Shc1](http://www.informatics.jax.org/searches/accession_report.cgi?id=MGI%3A98296) | [NM_011368](http://srs.sanger.ac.uk/srsbin/cgi-bin/wgetz?-e+%5BREFSEQ-ID:NM_011368%5D) | [U15784](http://www.ebi.ac.uk/cgi-bin/emblfetch?U15784) | SHC TRANSFORMING PROTEIN. |
| [M300021114](http://omad.operon.com/mouseV3/transcript.php?what=M300021114) | [9130413I22Rik](http://www.informatics.jax.org/searches/accession_report.cgi?id=MGI%3A1914818) | [NM_026242](http://srs.sanger.ac.uk/srsbin/cgi-bin/wgetz?-e+%5BREFSEQ-ID:NM_026242%5D) | [AB041651](http://www.ebi.ac.uk/cgi-bin/emblfetch?AB041651) | -- |
| [M300018312](http://omad.operon.com/mouseV3/transcript.php?what=M300018312) | -- | -- | -- | -- |
| [M300003187](http://omad.operon.com/mouseV3/transcript.php?what=M300003187) | -- | -- | -- | -- |
| [M300001659](http://omad.operon.com/mouseV3/transcript.php?what=M300001659) | [Kpna2](http://www.informatics.jax.org/searches/accession_report.cgi?id=MGI%3A103561) | [NM_010655](http://srs.sanger.ac.uk/srsbin/cgi-bin/wgetz?-e+%5BREFSEQ-ID:NM_010655%5D) | [BC006720](http://www.ebi.ac.uk/cgi-bin/emblfetch?BC006720) | IMPORTIN ALPHA-2 SUBUNIT (KARYOPHERIN ALPHA-2 SUBUNIT) (SRP1-ALPHA) (RAG COHORT PROTEIN 1) (PENDULIN) (PORE TARGETING COMPLEX 58 KDA SUBUNIT) (PTAC58) (IMPORTIN ALPHA P1). |
| [M300011584](http://omad.operon.com/mouseV3/transcript.php?what=M300011584) | -- | -- | -- | -- |
| [M300018684](http://omad.operon.com/mouseV3/transcript.php?what=M300018684) | [Kpna2](http://www.informatics.jax.org/searches/accession_report.cgi?id=MGI%3A103561) | [NM_010655](http://srs.sanger.ac.uk/srsbin/cgi-bin/wgetz?-e+%5BREFSEQ-ID:NM_010655%5D) | [BC006720](http://www.ebi.ac.uk/cgi-bin/emblfetch?BC006720) | IMPORTIN ALPHA-2 SUBUNIT (KARYOPHERIN ALPHA-2 SUBUNIT) (SRP1-ALPHA) (RAG COHORT PROTEIN 1) (PENDULIN) (PORE TARGETING COMPLEX 58 KDA SUBUNIT) (PTAC58) (IMPORTIN ALPHA P1). |
| [M300005759](http://omad.operon.com/mouseV3/transcript.php?what=M300005759) | [Ube2v1](http://www.informatics.jax.org/searches/accession_report.cgi?id=MGI%3A1913839) | -- | [BC019372](http://www.ebi.ac.uk/cgi-bin/emblfetch?BC019372) | SIMILAR TO UBIQUITIN-CONJUGATING ENZYME E2 VARIANT 1 (EC 6.3.2.19) (UBIQUITIN-PROTEIN LIGASE) (UBIQUITIN CARRIER PROTEIN). |
| [M200014015](http://omad.operon.com/mouseV3/transcript.php?what=M200014015) | [Lgals1](http://www.informatics.jax.org/searches/accession_report.cgi?id=MGI%3A96777) | [NM_008495](http://srs.sanger.ac.uk/srsbin/cgi-bin/wgetz?-e+%5BREFSEQ-ID:NM_008495%5D) | [AK004298](http://www.ebi.ac.uk/cgi-bin/emblfetch?AK004298) | GALECTIN-1 (BETA-GALACTOSIDE-BINDING LECTIN L-14-I) (LACTOSE-BINDING LECTIN 1) (S-LAC LECTIN 1) (GALAPTIN) (14 KDA LECTIN). |
| [M200000746](http://omad.operon.com/mouseV3/transcript.php?what=M200000746) | [Calr](http://www.informatics.jax.org/searches/accession_report.cgi?id=MGI%3A88252) | [NM_007591](http://srs.sanger.ac.uk/srsbin/cgi-bin/wgetz?-e+%5BREFSEQ-ID:NM_007591%5D) | [M92988](http://www.ebi.ac.uk/cgi-bin/emblfetch?M92988) | CALRETICULIN PRECURSOR (CRP55) (CALREGULIN) (HACBP) (ERP60). |
|  |  |  |  |  |
| **Female C57Bl6 day 7 200 Gy** | |  |  |  |
| [M200004758](http://omad.operon.com/mouseV3/transcript.php?what=M200004758) | [Blvrb](http://www.informatics.jax.org/searches/accession_report.cgi?id=MGI%3A2385271) | [NM_144923](http://srs.sanger.ac.uk/srsbin/cgi-bin/wgetz?-e+%5BREFSEQ-ID:NM_144923%5D) | [BC027279](http://www.ebi.ac.uk/cgi-bin/emblfetch?BC027279) | BILIVERDIN REDUCTASE B (FLAVIN REDUCTASE (NADPH)). |
| [M300003852](http://omad.operon.com/mouseV3/transcript.php?what=M300003852) | [Treml1-pending](http://www.informatics.jax.org/searches/accession_report.cgi?id=MGI%3A1918576) | -- | [AK017256](http://www.ebi.ac.uk/cgi-bin/emblfetch?AK017256) | -- |
| [M300007590](http://omad.operon.com/mouseV3/transcript.php?what=M300007590) | -- | [NM_172479](http://srs.sanger.ac.uk/srsbin/cgi-bin/wgetz?-e+%5BREFSEQ-ID:NM_172479%5D) | -- | -- |
| [M300005240](http://omad.operon.com/mouseV3/transcript.php?what=M300005240) | [Mgst3](http://www.informatics.jax.org/searches/accession_report.cgi?id=MGI%3A1913697) | [NM_025569](http://srs.sanger.ac.uk/srsbin/cgi-bin/wgetz?-e+%5BREFSEQ-ID:NM_025569%5D) | [BC029669](http://www.ebi.ac.uk/cgi-bin/emblfetch?BC029669) | MICROSOMAL GLUTATHIONE S-TRANSFERASE 3. |
| [M200000621](http://omad.operon.com/mouseV3/transcript.php?what=M200000621) | [Gpc4](http://www.informatics.jax.org/searches/accession_report.cgi?id=MGI%3A104902) | [NM_008150](http://srs.sanger.ac.uk/srsbin/cgi-bin/wgetz?-e+%5BREFSEQ-ID:NM_008150%5D) | [X83577](http://www.ebi.ac.uk/cgi-bin/emblfetch?X83577) | GLYPICAN-4 PRECURSOR (K-GLYPICAN). |
| [M300006292](http://omad.operon.com/mouseV3/transcript.php?what=M300006292) | [1810017F10Rik](http://www.informatics.jax.org/searches/accession_report.cgi?id=MGI%3A1913510) | [NM_025452](http://srs.sanger.ac.uk/srsbin/cgi-bin/wgetz?-e+%5BREFSEQ-ID:NM_025452%5D) | [AK008935](http://www.ebi.ac.uk/cgi-bin/emblfetch?AK008935) | BETA-CASEIN-LIKE. |
| [M300004473](http://omad.operon.com/mouseV3/transcript.php?what=M300004473) | [4833406P10Rik](http://www.informatics.jax.org/searches/accession_report.cgi?id=MGI%3A2442818) | -- | [AF404774](http://www.ebi.ac.uk/cgi-bin/emblfetch?AF404774) | ACTIN-BINDING LIM PROTEIN 1 MEDIUM ISOFORM. |
| [M300005665](http://omad.operon.com/mouseV3/transcript.php?what=M300005665) | [2010011I20Rik](http://www.informatics.jax.org/searches/accession_report.cgi?id=MGI%3A1914267) | [NM_025912](http://srs.sanger.ac.uk/srsbin/cgi-bin/wgetz?-e+%5BREFSEQ-ID:NM_025912%5D) | [BC016210](http://www.ebi.ac.uk/cgi-bin/emblfetch?BC016210) | -- |
| [M200015276](http://omad.operon.com/mouseV3/transcript.php?what=M200015276) | [Pep4](http://www.informatics.jax.org/searches/accession_report.cgi?id=MGI%3A97542) | [NM_008820](http://srs.sanger.ac.uk/srsbin/cgi-bin/wgetz?-e+%5BREFSEQ-ID:NM_008820%5D) | [D82983](http://www.ebi.ac.uk/cgi-bin/emblfetch?D82983) | XAA-PRO DIPEPTIDASE (EC 3.4.13.9) (X-PRO DIPEPTIDASE) (PROLINE DIPEPTIDASE) (PROLIDASE) (IMIDODIPEPTIDASE) (PEPTIDASE 4). |
| [M300000073](http://omad.operon.com/mouseV3/transcript.php?what=M300000073) | [Myf5](http://www.informatics.jax.org/searches/accession_report.cgi?id=MGI%3A97252) | [NM_008656](http://srs.sanger.ac.uk/srsbin/cgi-bin/wgetz?-e+%5BREFSEQ-ID:NM_008656%5D) | [X56182](http://www.ebi.ac.uk/cgi-bin/emblfetch?X56182) | MYOGENIC FACTOR MYF-5. |
| [M300002998](http://omad.operon.com/mouseV3/transcript.php?what=M300002998) | [Nisch](http://www.informatics.jax.org/searches/accession_report.cgi?id=MGI%3A1928323) | [NM_022656](http://srs.sanger.ac.uk/srsbin/cgi-bin/wgetz?-e+%5BREFSEQ-ID:NM_022656%5D) | [AF315344](http://www.ebi.ac.uk/cgi-bin/emblfetch?AF315344) | NISCHARIN; IMIDAZOLINE RECEPTOR I-1-LIKE PROTEIN. |
| [M300008241](http://omad.operon.com/mouseV3/transcript.php?what=M300008241) | [1110005A05Rik](http://www.informatics.jax.org/searches/accession_report.cgi?id=MGI%3A1921571) | [NM_025372](http://srs.sanger.ac.uk/srsbin/cgi-bin/wgetz?-e+%5BREFSEQ-ID:NM_025372%5D) | [AK003451](http://www.ebi.ac.uk/cgi-bin/emblfetch?AK003451) | -- |
| [M300002598](http://omad.operon.com/mouseV3/transcript.php?what=M300002598) | -- | -- | [AF206023](http://www.ebi.ac.uk/cgi-bin/emblfetch?AF206023) | ANTI-MYOSIN IMMUNOGLOBULIN HEAVY CHAIN VARIABLE REGION (FRAGMENT). |
| [M200004350](http://omad.operon.com/mouseV3/transcript.php?what=M200004350) | -- | -- | [BC024401](http://www.ebi.ac.uk/cgi-bin/emblfetch?BC024401) | SIMILAR TO DC12 PROTEIN. |
| [M300007147](http://omad.operon.com/mouseV3/transcript.php?what=M300007147) | -- | -- | -- | -- |
| [M200009417](http://omad.operon.com/mouseV3/transcript.php?what=M200009417) | [Mt2](http://www.informatics.jax.org/searches/accession_report.cgi?id=MGI%3A97172) | -- | [K02236](http://www.ebi.ac.uk/cgi-bin/emblfetch?K02236) | METALLOTHIONEIN-II (MT-II). |
| [M300022215](http://omad.operon.com/mouseV3/transcript.php?what=M300022215) | -- | -- | -- | -- |
| [M200014231](http://omad.operon.com/mouseV3/transcript.php?what=M200014231) | [Supt16h](http://www.informatics.jax.org/searches/accession_report.cgi?id=MGI%3A1890948) | [NM_033618](http://srs.sanger.ac.uk/srsbin/cgi-bin/wgetz?-e+%5BREFSEQ-ID:NM_033618%5D) | [AF323667](http://www.ebi.ac.uk/cgi-bin/emblfetch?AF323667) | SUPPRESSOR OF TY 16 HOMOLOG; SUPPRESSOR OF TY 16 HOMOLOG (S.CEREVISIAE). |
| [M300016699](http://omad.operon.com/mouseV3/transcript.php?what=M300016699) | -- | -- | [AK011630](http://www.ebi.ac.uk/cgi-bin/emblfetch?AK011630) | -- |
| [M300015461](http://omad.operon.com/mouseV3/transcript.php?what=M300015461) | -- | -- | -- | -- |
| [M300006903](http://omad.operon.com/mouseV3/transcript.php?what=M300006903) | -- | [NM_007624](http://srs.sanger.ac.uk/srsbin/cgi-bin/wgetz?-e+%5BREFSEQ-ID:NM_007624%5D) | -- | CHROMOBOX HOMOLOG 3 (DROSOPHILA HP1 GAMMA); HETEROCHROMATIN PROTEIN 1 GAMMA. |
| [M300002502](http://omad.operon.com/mouseV3/transcript.php?what=M300002502) | [Pnn](http://www.informatics.jax.org/searches/accession_report.cgi?id=MGI%3A1100514) | [NM_008891](http://srs.sanger.ac.uk/srsbin/cgi-bin/wgetz?-e+%5BREFSEQ-ID:NM_008891%5D) | [Y08701](http://www.ebi.ac.uk/cgi-bin/emblfetch?Y08701) | PININ; DNA SEGMENT, CHR 12, ERATO DOI 512, EXPRESSED. |
| [M200000746](http://omad.operon.com/mouseV3/transcript.php?what=M200000746) | [Calr](http://www.informatics.jax.org/searches/accession_report.cgi?id=MGI%3A88252) | [NM_007591](http://srs.sanger.ac.uk/srsbin/cgi-bin/wgetz?-e+%5BREFSEQ-ID:NM_007591%5D) | [M92988](http://www.ebi.ac.uk/cgi-bin/emblfetch?M92988) | CALRETICULIN PRECURSOR (CRP55) (CALREGULIN) (HACBP) (ERP60). |
| [M200009655](http://omad.operon.com/mouseV3/transcript.php?what=M200009655) | [Cct6a](http://www.informatics.jax.org/searches/accession_report.cgi?id=MGI%3A107943) | [NM_009838](http://srs.sanger.ac.uk/srsbin/cgi-bin/wgetz?-e+%5BREFSEQ-ID:NM_009838%5D) | [AB022159](http://www.ebi.ac.uk/cgi-bin/emblfetch?AB022159) | T-COMPLEX PROTEIN 1, ZETA SUBUNIT (TCP-1-ZETA) (CCT-ZETA) (CCT-ZETA- 1). |
| [M300011584](http://omad.operon.com/mouseV3/transcript.php?what=M300011584) | -- | -- | -- | -- |
