# Supplementary material for: Gene Expression Signatures of Radiation Response Are Specific, Durable and Accurate in Mice and Humans
Source: PLoS One. 2008 Apr 2;3(4):e1912. doi: 10.1371/journal.pone.0001912 (PMC2271127; doi:10.1371/journal.pone.0001912)
Supplement: Table S5 — (0.08 MB DOC) [file pone.0001912.s005.doc]

Table S5. Genes that distinguish LPS treatment in C57Bl6 mice. Operon Oligo ID can be queried in the OMAD database ([http://omad.operon.com](http://omad.operon.com/))

| **Operon Oligo ID** | **Gene Symbol** | **RefSeq** | **Genbank** | **Description** |
| --- | --- | --- | --- | --- |
| [M200003295](http://omad.operon.com/mouseV3/transcript.php?what=M200003295) | [Saa3](http://www.informatics.jax.org/searches/accession_report.cgi?id=MGI%3A98223) | [NM_011315](http://srs.sanger.ac.uk/srsbin/cgi-bin/wgetz?-e+%5BREFSEQ-ID:NM_011315%5D) | [M17792](http://www.ebi.ac.uk/cgi-bin/emblfetch?M17792) | SERUM AMYLOID A-3 PROTEIN PRECURSOR. |
| [M300009870](http://omad.operon.com/mouseV3/transcript.php?what=M300009870) | [Ccl12](http://www.informatics.jax.org/searches/accession_report.cgi?id=MGI%3A108224) | [NM_011331](http://srs.sanger.ac.uk/srsbin/cgi-bin/wgetz?-e+%5BREFSEQ-ID:NM_011331%5D) | [AF065938](http://www.ebi.ac.uk/cgi-bin/emblfetch?AF065938) | SMALL INDUCIBLE CYTOKINE A12 PRECURSOR (CCL12) (MONOCYTE CHEMOTACTIC PROTEIN 5) (MCP-5) (MCP-1 RELATED CHEMOKINE). |
| [M300005418](http://omad.operon.com/mouseV3/transcript.php?what=M300005418) | [Il1rn](http://www.informatics.jax.org/searches/accession_report.cgi?id=MGI%3A96547) | [NM_031167](http://srs.sanger.ac.uk/srsbin/cgi-bin/wgetz?-e+%5BREFSEQ-ID:NM_031167%5D) | [S64082](http://www.ebi.ac.uk/cgi-bin/emblfetch?S64082) | INTERLEUKIN-1 RECEPTOR ANTAGONIST PROTEIN PRECURSOR (IL-1RA) (IL-1RN) (IRAP). |
| [M200001838](http://omad.operon.com/mouseV3/transcript.php?what=M200001838) | [Upp](http://www.informatics.jax.org/searches/accession_report.cgi?id=MGI%3A1097668) | [NM_009477](http://srs.sanger.ac.uk/srsbin/cgi-bin/wgetz?-e+%5BREFSEQ-ID:NM_009477%5D) | [D44464](http://www.ebi.ac.uk/cgi-bin/emblfetch?D44464) | URIDINE PHOSPHORYLASE (EC 2.4.2.3) (UDRPASE). |
| [M200000053](http://omad.operon.com/mouseV3/transcript.php?what=M200000053) | [Fcgr1](http://www.informatics.jax.org/searches/accession_report.cgi?id=MGI%3A95498) | [NM_010186](http://srs.sanger.ac.uk/srsbin/cgi-bin/wgetz?-e+%5BREFSEQ-ID:NM_010186%5D) | [BC025535](http://www.ebi.ac.uk/cgi-bin/emblfetch?BC025535) | HIGH AFFINITY IMMUNOGLOBULIN GAMMA FC RECEPTOR I PRECURSOR (FC-GAMMA RI) (FCRI) (IGG FC RECEPTOR I). |
| [M200004157](http://omad.operon.com/mouseV3/transcript.php?what=M200004157) | [9130009C22Rik](http://www.informatics.jax.org/searches/accession_report.cgi?id=MGI%3A1918836) | [NM_027835](http://srs.sanger.ac.uk/srsbin/cgi-bin/wgetz?-e+%5BREFSEQ-ID:NM_027835%5D) | [AF374384](http://www.ebi.ac.uk/cgi-bin/emblfetch?AF374384) | -- |
| [M300005305](http://omad.operon.com/mouseV3/transcript.php?what=M300005305) | [Lcn2](http://www.informatics.jax.org/searches/accession_report.cgi?id=MGI%3A96757) | -- | [X81627](http://www.ebi.ac.uk/cgi-bin/emblfetch?X81627) | NEUTROPHIL GELATINASE-ASSOCIATED LIPOCALIN PRECURSOR (NGAL) (P25) (SV-40 INDUCED 24P3 PROTEIN) (LIPOCALIN 2). |
| [M300006479](http://omad.operon.com/mouseV3/transcript.php?what=M300006479) | [Bst1](http://www.informatics.jax.org/searches/accession_report.cgi?id=MGI%3A105370) | [NM_009763](http://srs.sanger.ac.uk/srsbin/cgi-bin/wgetz?-e+%5BREFSEQ-ID:NM_009763%5D) | [D31788](http://www.ebi.ac.uk/cgi-bin/emblfetch?D31788) | ADP-RIBOSYL CYCLASE 2 PRECURSOR (EC 3.2.2.5) (CYCLIC ADP-RIBOSE HYDROLASE 2) (CADPR HYDROLASE 2) (BONE MARROW STROMAL ANTIGEN 1) (BST- 1) (BP-3 ALLOANTIGEN) (ANTIGEN BP3). |
| [M200004765](http://omad.operon.com/mouseV3/transcript.php?what=M200004765) | [Gbp2](http://www.informatics.jax.org/searches/accession_report.cgi?id=MGI%3A102772) | [NM_010260](http://srs.sanger.ac.uk/srsbin/cgi-bin/wgetz?-e+%5BREFSEQ-ID:NM_010260%5D) | [AF077007](http://www.ebi.ac.uk/cgi-bin/emblfetch?AF077007) | GUANYLATE NUCLEOTIDE BINDING PROTEIN 2. |
| [M300005673](http://omad.operon.com/mouseV3/transcript.php?what=M300005673) | [Zbp1](http://www.informatics.jax.org/searches/accession_report.cgi?id=MGI%3A1927449) | [NM_021394](http://srs.sanger.ac.uk/srsbin/cgi-bin/wgetz?-e+%5BREFSEQ-ID:NM_021394%5D) | [BC020033](http://www.ebi.ac.uk/cgi-bin/emblfetch?BC020033) | Z-DNA BINDING PROTEIN 1 (TUMOR STROMA AND ACTIVATED MACROPHAGE PROTEIN DLM-1). |
| [M300005674](http://omad.operon.com/mouseV3/transcript.php?what=M300005674) | [Zbp1](http://www.informatics.jax.org/searches/accession_report.cgi?id=MGI%3A1927449) | [NM_021394](http://srs.sanger.ac.uk/srsbin/cgi-bin/wgetz?-e+%5BREFSEQ-ID:NM_021394%5D) | [BC020033](http://www.ebi.ac.uk/cgi-bin/emblfetch?BC020033) | Z-DNA BINDING PROTEIN 1 (TUMOR STROMA AND ACTIVATED MACROPHAGE PROTEIN DLM-1). |
| [M300001891](http://omad.operon.com/mouseV3/transcript.php?what=M300001891) | [Gp49b](http://www.informatics.jax.org/searches/accession_report.cgi?id=MGI%3A102701) | [NM_013532](http://srs.sanger.ac.uk/srsbin/cgi-bin/wgetz?-e+%5BREFSEQ-ID:NM_013532%5D) | [U05264](http://www.ebi.ac.uk/cgi-bin/emblfetch?U05264) | MAST CELL SURFACE GLYCOPROTEIN GP49B PRECURSOR. |
| [M300005166](http://omad.operon.com/mouseV3/transcript.php?what=M300005166) | [Ifi204](http://www.informatics.jax.org/searches/accession_report.cgi?id=MGI%3A96429) | [NM_008329](http://srs.sanger.ac.uk/srsbin/cgi-bin/wgetz?-e+%5BREFSEQ-ID:NM_008329%5D) | [M31419](http://www.ebi.ac.uk/cgi-bin/emblfetch?M31419) | INTERFERON-ACTIVATABLE PROTEIN 204 (IFI-204) (INTERFERON-INDUCIBLE PROTEIN P204). |
| [M200005576](http://omad.operon.com/mouseV3/transcript.php?what=M200005576) | [Usp18](http://www.informatics.jax.org/searches/accession_report.cgi?id=MGI%3A1344364) | [NM_011909](http://srs.sanger.ac.uk/srsbin/cgi-bin/wgetz?-e+%5BREFSEQ-ID:NM_011909%5D) | [AF069502](http://www.ebi.ac.uk/cgi-bin/emblfetch?AF069502) | UBL CARBOXYL-TERMINAL HYDROLASE 18 (EC 3.1.2.-) (UBL THIOLESTERASE 18) (ISG15-SPECIFIC PROCESSING PROTEASE) (43 KDA ISG15-SPECIFIC PROTEASE). |
| [M300020771](http://omad.operon.com/mouseV3/transcript.php?what=M300020771) | -- | -- | -- | -- |
| [M300011591](http://omad.operon.com/mouseV3/transcript.php?what=M300011591) | -- | [NM_172893](http://srs.sanger.ac.uk/srsbin/cgi-bin/wgetz?-e+%5BREFSEQ-ID:NM_172893%5D) | [BC024579](http://www.ebi.ac.uk/cgi-bin/emblfetch?BC024579) | -- |
| [M200007439](http://omad.operon.com/mouseV3/transcript.php?what=M200007439) | [Gtpi-pending](http://www.informatics.jax.org/searches/accession_report.cgi?id=MGI%3A1926262) | [NM_019440](http://srs.sanger.ac.uk/srsbin/cgi-bin/wgetz?-e+%5BREFSEQ-ID:NM_019440%5D) | [AJ007972](http://www.ebi.ac.uk/cgi-bin/emblfetch?AJ007972) | INTERFERON-G INDUCED GTPASE. |
| [M300012693](http://omad.operon.com/mouseV3/transcript.php?what=M300012693) | -- | -- | -- | -- |
| [M300012210](http://omad.operon.com/mouseV3/transcript.php?what=M300012210) | -- | -- | -- | -- |
| [M200014281](http://omad.operon.com/mouseV3/transcript.php?what=M200014281) | [2010008K16Rik](http://www.informatics.jax.org/searches/accession_report.cgi?id=MGI%3A1917360) | [NM_027320](http://srs.sanger.ac.uk/srsbin/cgi-bin/wgetz?-e+%5BREFSEQ-ID:NM_027320%5D) | [BC008158](http://www.ebi.ac.uk/cgi-bin/emblfetch?BC008158) | INTERFERON-INDUCED 35 KDA PROTEIN HOMOLOG (IFP 35). |
| [M300009340](http://omad.operon.com/mouseV3/transcript.php?what=M300009340) | -- | [NM_145481](http://srs.sanger.ac.uk/srsbin/cgi-bin/wgetz?-e+%5BREFSEQ-ID:NM_145481%5D) | [BC021340](http://www.ebi.ac.uk/cgi-bin/emblfetch?BC021340) | -- |
| [M200004564](http://omad.operon.com/mouseV3/transcript.php?what=M200004564) | [Nte](http://www.informatics.jax.org/searches/accession_report.cgi?id=MGI%3A1354723) | [NM_015801](http://srs.sanger.ac.uk/srsbin/cgi-bin/wgetz?-e+%5BREFSEQ-ID:NM_015801%5D) | [AF173829](http://www.ebi.ac.uk/cgi-bin/emblfetch?AF173829) | NEUROPATHY TARGET ESTERASE; SWISS CHEESE. |
| [M300000152](http://omad.operon.com/mouseV3/transcript.php?what=M300000152) | [Araf](http://www.informatics.jax.org/searches/accession_report.cgi?id=MGI%3A88065) | [NM_009703](http://srs.sanger.ac.uk/srsbin/cgi-bin/wgetz?-e+%5BREFSEQ-ID:NM_009703%5D) | [D00024](http://www.ebi.ac.uk/cgi-bin/emblfetch?D00024) | A-RAF PROTO-ONCOGENE SERINE/THREONINE-PROTEIN KINASE (EC 2.7.1.-). |
| [M200006264](http://omad.operon.com/mouseV3/transcript.php?what=M200006264) | -- | [NM_176831](http://srs.sanger.ac.uk/srsbin/cgi-bin/wgetz?-e+%5BREFSEQ-ID:NM_176831%5D) | -- | -- |
| [M300000077](http://omad.operon.com/mouseV3/transcript.php?what=M300000077) | [D15Ertd417e](http://www.informatics.jax.org/searches/accession_report.cgi?id=MGI%3A1196439) | [NM_144811](http://srs.sanger.ac.uk/srsbin/cgi-bin/wgetz?-e+%5BREFSEQ-ID:NM_144811%5D) | [BC021398](http://www.ebi.ac.uk/cgi-bin/emblfetch?BC021398) | CHROMOBOX PROTEIN HOMOLOG 6. |
