# Supplementary material for: Gene Expression Signatures of Radiation Response Are Specific, Durable and Accurate in Mice and Humans
Source: PLoS One. 2008 Apr 2;3(4):e1912. doi: 10.1371/journal.pone.0001912 (PMC2271127; doi:10.1371/journal.pone.0001912)
Supplement: Table S6 — (0.03 MB DOC) [file pone.0001912.s006.doc]

Table S6. Donor Patient Characteristics

_______________________________________________________

Characteristic Number

*Samples analyzed* n=18 healthy donors

n=36 patients pre-radiotherapy

n=34 patients post-radiotherapy

n=36 patients pre-chemotherapy

n=32 patients post-chemotherapy

*Patient/Donor Age* 47.9 years (mean)

*Diagnoses* MDS/AML (n=23)

ALL (n=8)

Multiple myeloma (n=20)

Non-Hodgkin’s Lymphoma (n=20)

Hodgkin’s Disease (n=6)

Myeloproliferative disorder (n=7)

Scleroderma (n=3)

Sickle cell disease (n=1)

*Prior radiotherapy* n=15

*Prior chemotherapy*  n=82

*Transplantation type* Non-myeloablative allogeneic/200 cGy (n=24)

Myeloablative allogeneic/1350 cGy (n=15)

Myeloablative autologous/1200 cGy (n=8)

Chemotherapy allogeneic (n=19)

Chemotherapy autologous (n=22)

Patients undergoing either TBI-based or chemotherapy-based conditioning followed by allogeneic or autologous stem cell transplantation were eligible for enrollment. PB samples were collected prior to and 6 hours following either 200 cGy total body irradiation (non-myeloablative conditioning) or the first fraction (150 cGy) of total body irradiation (myeloablative conditioning). MDS=myelodysplastic syndrome, AML=acute myelogenous leukemia, ALL=acute lymphocytic leukemia
