# Supplementary material for: Gene Expression Signatures of Radiation Response Are Specific, Durable and Accurate in Mice and Humans
Source: PLoS One. 2008 Apr 2;3(4):e1912. doi: 10.1371/journal.pone.0001912 (PMC2271127; doi:10.1371/journal.pone.0001912)
Supplement: Table S7 — (0.08 MB DOC) [file pone.0001912.s007.doc]

Table S7. Genes that distinguish radiation status in humans. Operon Oligo ID can be queried in the OMAD database ([http://omad.operon.com](http://omad.operon.com/))

| **Operon Oligo_ID** | **Gene Symbol** | **RefSeq** | **Genbank** | **Description** |
| --- | --- | --- | --- | --- |
| [H200000088](http://omad.operon.com/humanV3/transcript.php?what=H200000088) | [XPC](http://bioinfo.weizmann.ac.il/cards-bin/cardsearch.pl?search=XPC) | [NM_004628](http://srs.sanger.ac.uk/srsbin/cgi-bin/wgetz?-e+%5BREFSEQ-ID:NM_004628%5D) | [X65024](http://www.ebi.ac.uk/cgi-bin/emblfetch?X65024) | DNA-REPAIR PROTEIN COMPLEMENTING XP-C CELLS (XERODERMA PIGMENTOSUM GROUP C COMPLEMENTING PROTEIN) (P125) |
| [H200001266](http://omad.operon.com/humanV3/transcript.php?what=H200001266) | -- | [NM_017792](http://srs.sanger.ac.uk/srsbin/cgi-bin/wgetz?-e+%5BREFSEQ-ID:NM_017792%5D) | [AK000380](http://www.ebi.ac.uk/cgi-bin/emblfetch?AK000380) | -- |
| [H200002100](http://omad.operon.com/humanV3/transcript.php?what=H200002100) | -- | [NM_024556](http://srs.sanger.ac.uk/srsbin/cgi-bin/wgetz?-e+%5BREFSEQ-ID:NM_024556%5D) | [BC001340](http://www.ebi.ac.uk/cgi-bin/emblfetch?BC001340) | -- |
| [H200002529](http://omad.operon.com/humanV3/transcript.php?what=H200002529) | -- | [NM_032324](http://srs.sanger.ac.uk/srsbin/cgi-bin/wgetz?-e+%5BREFSEQ-ID:NM_032324%5D) | [AF416713](http://www.ebi.ac.uk/cgi-bin/emblfetch?AF416713) | -- |
| [H200004865](http://omad.operon.com/humanV3/transcript.php?what=H200004865) | -- | [NM_006828](http://srs.sanger.ac.uk/srsbin/cgi-bin/wgetz?-e+%5BREFSEQ-ID:NM_006828%5D) | [AL834463](http://www.ebi.ac.uk/cgi-bin/emblfetch?AL834463) | DJ467N11.1 PROTEIN |
| [H200006009](http://omad.operon.com/humanV3/transcript.php?what=H200006009) | [GTF3A](http://bioinfo.weizmann.ac.il/cards-bin/cardsearch.pl?search=GTF3A) | [NM_002097](http://srs.sanger.ac.uk/srsbin/cgi-bin/wgetz?-e+%5BREFSEQ-ID:NM_002097%5D) | [U14134](http://www.ebi.ac.uk/cgi-bin/emblfetch?U14134) | TRANSCRIPTION FACTOR IIIA (FACTOR A) (TFIIIA) |
| [H200006598](http://omad.operon.com/humanV3/transcript.php?what=H200006598) | [PCNA](http://bioinfo.weizmann.ac.il/cards-bin/cardsearch.pl?search=PCNA) | [NM_002592](http://srs.sanger.ac.uk/srsbin/cgi-bin/wgetz?-e+%5BREFSEQ-ID:NM_002592%5D) | [BC000491](http://www.ebi.ac.uk/cgi-bin/emblfetch?BC000491) | PROLIFERATING CELL NUCLEAR ANTIGEN (PCNA) (CYCLIN) |
| [H200008365](http://omad.operon.com/humanV3/transcript.php?what=H200008365) | [CDKN1A](http://bioinfo.weizmann.ac.il/cards-bin/cardsearch.pl?search=CDKN1A) | [NM_000389](http://srs.sanger.ac.uk/srsbin/cgi-bin/wgetz?-e+%5BREFSEQ-ID:NM_000389%5D) | [BC013967](http://www.ebi.ac.uk/cgi-bin/emblfetch?BC013967) | CYCLIN-DEPENDENT KINASE INHIBITOR 1 (P21) (CDK-INTERACTING PROTEIN 1) (MELANOMA DIFFERENTIATION ASSOCIATED PROTEIN 6) (MDA-6) |
| [H200011100](http://omad.operon.com/humanV3/transcript.php?what=H200011100) | [PPM1D](http://bioinfo.weizmann.ac.il/cards-bin/cardsearch.pl?search=PPM1D) | [NM_003620](http://srs.sanger.ac.uk/srsbin/cgi-bin/wgetz?-e+%5BREFSEQ-ID:NM_003620%5D) | [BC033893](http://www.ebi.ac.uk/cgi-bin/emblfetch?BC033893) | PROTEIN PHOSPHATASE 2C DELTA ISOFORM (PP2C-DELTA) (P53- INDUCED PROTEIN PHOSPHATASE 1) (PROTEIN PHOSPHATASE MAGNESIUM- DEPENDENT 1 DELTA) |
| [H200011577](http://omad.operon.com/humanV3/transcript.php?what=H200011577) | -- | [NM_018247](http://srs.sanger.ac.uk/srsbin/cgi-bin/wgetz?-e+%5BREFSEQ-ID:NM_018247%5D) | [AK001718](http://www.ebi.ac.uk/cgi-bin/emblfetch?AK001718) | -- |
| [H200014322](http://omad.operon.com/humanV3/transcript.php?what=H200014322) | -- | -- | [BC009552](http://www.ebi.ac.uk/cgi-bin/emblfetch?BC009552) | CGI-203 |
| [H200014719](http://omad.operon.com/humanV3/transcript.php?what=H200014719) | [ACTA2](http://bioinfo.weizmann.ac.il/cards-bin/cardsearch.pl?search=ACTA2) | [NM_001613](http://srs.sanger.ac.uk/srsbin/cgi-bin/wgetz?-e+%5BREFSEQ-ID:NM_001613%5D) | [X60732](http://www.ebi.ac.uk/cgi-bin/emblfetch?X60732) | ACTIN, AORTIC SMOOTH MUSCLE (ALPHA-ACTIN 2) |
| [H200016323](http://omad.operon.com/humanV3/transcript.php?what=H200016323) | -- | [NM_152240](http://srs.sanger.ac.uk/srsbin/cgi-bin/wgetz?-e+%5BREFSEQ-ID:NM_152240%5D) | [BC002896](http://www.ebi.ac.uk/cgi-bin/emblfetch?BC002896) | P53 TARGET ZINC FINGER PROTEIN ISOFORM 1; ZINC FINGER PROTEIN WIG1; WIG-1/PAG608 PROTEIN |
| [H200017549](http://omad.operon.com/humanV3/transcript.php?what=H200017549) | [TIMM8B](http://bioinfo.weizmann.ac.il/cards-bin/cardsearch.pl?search=TIMM8B) | [NM_012459](http://srs.sanger.ac.uk/srsbin/cgi-bin/wgetz?-e+%5BREFSEQ-ID:NM_012459%5D) | [BC000711](http://www.ebi.ac.uk/cgi-bin/emblfetch?BC000711) | MITOCHONDRIAL IMPORT INNER MEMBRANE TRANSLOCASE SUBUNIT TIM8 B (DEAFNESS DYSTONIA PROTEIN 2) (DDP-LIKE PROTEIN) |
| [H300000421](http://omad.operon.com/humanV3/transcript.php?what=H300000421) | -- | [NM_016399](http://srs.sanger.ac.uk/srsbin/cgi-bin/wgetz?-e+%5BREFSEQ-ID:NM_016399%5D) | [BC002638](http://www.ebi.ac.uk/cgi-bin/emblfetch?BC002638) | PROTEIN 15E1.1 (PROTEIN HSPC132) |
| [H300003103](http://omad.operon.com/humanV3/transcript.php?what=H300003103) | -- | -- | -- | -- |
| [H300003151](http://omad.operon.com/humanV3/transcript.php?what=H300003151) | [MOAP1](http://bioinfo.weizmann.ac.il/cards-bin/cardsearch.pl?search=MOAP1) | [NM_022151](http://srs.sanger.ac.uk/srsbin/cgi-bin/wgetz?-e+%5BREFSEQ-ID:NM_022151%5D) | [BC015044](http://www.ebi.ac.uk/cgi-bin/emblfetch?BC015044) | MODULATOR OF APOPTOSIS 1; MAP-1 PROTEIN; PARANEOPLASTIC ANTIGEN LIKE 4 |
| [H300010830](http://omad.operon.com/humanV3/transcript.php?what=H300010830) | -- | [NM_022767](http://srs.sanger.ac.uk/srsbin/cgi-bin/wgetz?-e+%5BREFSEQ-ID:NM_022767%5D) | [BC005164](http://www.ebi.ac.uk/cgi-bin/emblfetch?BC005164) | -- |
| [H300015667](http://omad.operon.com/humanV3/transcript.php?what=H300015667) | -- | [NM_022767](http://srs.sanger.ac.uk/srsbin/cgi-bin/wgetz?-e+%5BREFSEQ-ID:NM_022767%5D) | [BC005164](http://www.ebi.ac.uk/cgi-bin/emblfetch?BC005164) | -- |
| [H300018970](http://omad.operon.com/humanV3/transcript.php?what=H300018970) | -- | [NM_014454](http://srs.sanger.ac.uk/srsbin/cgi-bin/wgetz?-e+%5BREFSEQ-ID:NM_014454%5D) | [AK001886](http://www.ebi.ac.uk/cgi-bin/emblfetch?AK001886) | SESTRIN 1 (P53-REGULATED PROTEIN PA26) |
| [H300019371](http://omad.operon.com/humanV3/transcript.php?what=H300019371) | [DDB2](http://bioinfo.weizmann.ac.il/cards-bin/cardsearch.pl?search=DDB2) | [NM_000107](http://srs.sanger.ac.uk/srsbin/cgi-bin/wgetz?-e+%5BREFSEQ-ID:NM_000107%5D) | [BC000093](http://www.ebi.ac.uk/cgi-bin/emblfetch?BC000093) | DNA DAMAGE BINDING PROTEIN 2 (DAMAGE-SPECIFIC DNA BINDING PROTEIN 2) (DDB P48 SUBUNIT) (DDBB) (UV-DAMAGED DNA-BINDING PROTEIN 2) (UV-DDB 2) |
| [H300020184](http://omad.operon.com/humanV3/transcript.php?what=H300020184) | [C19orf2](http://bioinfo.weizmann.ac.il/cards-bin/cardsearch.pl?search=C19orf2) | [NM_003796](http://srs.sanger.ac.uk/srsbin/cgi-bin/wgetz?-e+%5BREFSEQ-ID:NM_003796%5D) | [AB006572](http://www.ebi.ac.uk/cgi-bin/emblfetch?AB006572) | RNA POLYMERASE II SUBUNIT 5-MEDIATING PROTEIN (RPB5-MEDIATING PROTEIN) |
| [H300020858](http://omad.operon.com/humanV3/transcript.php?what=H300020858) | [HNRPDL](http://bioinfo.weizmann.ac.il/cards-bin/cardsearch.pl?search=HNRPDL) | [NM_005463](http://srs.sanger.ac.uk/srsbin/cgi-bin/wgetz?-e+%5BREFSEQ-ID:NM_005463%5D) | [BC011714](http://www.ebi.ac.uk/cgi-bin/emblfetch?BC011714) | HETEROGENEOUS NUCLEAR RIBONUCLEOPROTEIN D-LIKE; A+U-RICH ELEMENT RNA BINDING FACTOR |
| [H300021118](http://omad.operon.com/humanV3/transcript.php?what=H300021118) | [BBC3](http://bioinfo.weizmann.ac.il/cards-bin/cardsearch.pl?search=BBC3) | [NM_014417](http://srs.sanger.ac.uk/srsbin/cgi-bin/wgetz?-e+%5BREFSEQ-ID:NM_014417%5D) | [AF354655](http://www.ebi.ac.uk/cgi-bin/emblfetch?AF354655) | BCL2 BINDING COMPONENT 3; BCL-2 BINDING COMPONENT 3; PUMA/JFY1 PROTEIN; BCL-2 BINDING COMPONENT 3 |
| [H300022025](http://omad.operon.com/humanV3/transcript.php?what=H300022025) | [BAX](http://bioinfo.weizmann.ac.il/cards-bin/cardsearch.pl?search=BAX) | [NM_138763](http://srs.sanger.ac.uk/srsbin/cgi-bin/wgetz?-e+%5BREFSEQ-ID:NM_138763%5D) | [U19599](http://www.ebi.ac.uk/cgi-bin/emblfetch?U19599) | BAX PROTEIN, CYTOPLASMIC ISOFORM DELTA |
