# Supplementary material for: Gene Expression Signatures of Radiation Response Are Specific, Durable and Accurate in Mice and Humans
Source: PLoS One. 2008 Apr 2;3(4):e1912. doi: 10.1371/journal.pone.0001912 (PMC2271127; doi:10.1371/journal.pone.0001912)
Supplement: Table S8 — (0.08 MB DOC) [file pone.0001912.s008.doc]

Table S8. Genes that distinguish chemotherapy treatment in humans. Operon Oligo ID can be queried in the OMAD database ([http://omad.operon.com](http://omad.operon.com/))

| **Operon**  **oligo_ID** | **Gene Symbol** | **RefSeq** | **Genbank** | **Description** |
| --- | --- | --- | --- | --- |
| [H200001454](http://omad.operon.com/humanV3/transcript.php?what=H200001454) | [FKBP5](http://bioinfo.weizmann.ac.il/cards-bin/cardsearch.pl?search=FKBP5) | [NM_004117](http://srs.sanger.ac.uk/srsbin/cgi-bin/wgetz?-e+%5BREFSEQ-ID:NM_004117%5D) | [U42031](http://www.ebi.ac.uk/cgi-bin/emblfetch?U42031) | FK506-BINDING PROTEIN 5 (EC 5.2.1.8) (PEPTIDYL-PROLYL CIS-TRANS ISOMERASE) (PPIASE) (ROTAMASE) (51 KDA FK506-BINDING PROTEIN) (FKBP- 51) (54 KDA PROGESTERONE RECEPTOR-ASSOCIATED IMMUNOPHILIN) (FKBP54) (P54) (FF1 ANTIGEN) (HSP90-BINDING IMMUNOPHILIN) |
| [H200002954](http://omad.operon.com/humanV3/transcript.php?what=H200002954) | [SAP30](http://bioinfo.weizmann.ac.il/cards-bin/cardsearch.pl?search=SAP30) | [NM_003864](http://srs.sanger.ac.uk/srsbin/cgi-bin/wgetz?-e+%5BREFSEQ-ID:NM_003864%5D) | [BC016757](http://www.ebi.ac.uk/cgi-bin/emblfetch?BC016757) | SIN3 ASSOCIATED POLYPEPTIDE P30; SIN3-ASSOCIATED POLYPEPTIDE, 30KD |
| [H200004993](http://omad.operon.com/humanV3/transcript.php?what=H200004993) | [SOCS1](http://bioinfo.weizmann.ac.il/cards-bin/cardsearch.pl?search=SOCS1) | [NM_003745](http://srs.sanger.ac.uk/srsbin/cgi-bin/wgetz?-e+%5BREFSEQ-ID:NM_003745%5D) | [AB000676](http://www.ebi.ac.uk/cgi-bin/emblfetch?AB000676) | SUPPRESSOR OF CYTOKINE SIGNALING 1 (SOCS-1) (JAK-BINDING PROTEIN) (JAB) (STAT INDUCED STAT INHIBITOR 1) (SSI-1) (TEC-INTERACTING PROTEIN 3) (TIP-3) |
| [H200002479](http://omad.operon.com/humanV3/transcript.php?what=H200002479) | [CRAMP1L](http://bioinfo.weizmann.ac.il/cards-bin/cardsearch.pl?search=CRAMP1L) | -- | [AB037847](http://www.ebi.ac.uk/cgi-bin/emblfetch?AB037847) | -- |
| [H200020334](http://omad.operon.com/humanV3/transcript.php?what=H200020334) | -- | [NM_006372](http://srs.sanger.ac.uk/srsbin/cgi-bin/wgetz?-e+%5BREFSEQ-ID:NM_006372%5D) | [AY034482](http://www.ebi.ac.uk/cgi-bin/emblfetch?AY034482) | NS1-ASSOCIATED PROTEIN 1 |
| [H200002535](http://omad.operon.com/humanV3/transcript.php?what=H200002535) | -- | [NM_018034](http://srs.sanger.ac.uk/srsbin/cgi-bin/wgetz?-e+%5BREFSEQ-ID:NM_018034%5D) | [BC025315](http://www.ebi.ac.uk/cgi-bin/emblfetch?BC025315) | -- |
| [H200002231](http://omad.operon.com/humanV3/transcript.php?what=H200002231) | [UVRAG](http://bioinfo.weizmann.ac.il/cards-bin/cardsearch.pl?search=UVRAG) | [NM_003369](http://srs.sanger.ac.uk/srsbin/cgi-bin/wgetz?-e+%5BREFSEQ-ID:NM_003369%5D) | [AB012958](http://www.ebi.ac.uk/cgi-bin/emblfetch?AB012958) | UV RADIATION RESISTANCE-ASSOCIATED GENE PROTEIN (P63) |
| [H200002230](http://omad.operon.com/humanV3/transcript.php?what=H200002230) | -- | [NM_005475](http://srs.sanger.ac.uk/srsbin/cgi-bin/wgetz?-e+%5BREFSEQ-ID:NM_005475%5D) | [AJ012793](http://www.ebi.ac.uk/cgi-bin/emblfetch?AJ012793) | LYMPHOCYTE SPECIFIC ADAPTER PROTEIN LNK (SIGNAL TRANSDUCTION PROTEIN LNK) (LYMPHOCYTE ADAPTER PROTEIN) |
| [H300001588](http://omad.operon.com/humanV3/transcript.php?what=H300001588) | [ASGR1](http://bioinfo.weizmann.ac.il/cards-bin/cardsearch.pl?search=ASGR1) | [NM_001671](http://srs.sanger.ac.uk/srsbin/cgi-bin/wgetz?-e+%5BREFSEQ-ID:NM_001671%5D) | [AB070933](http://www.ebi.ac.uk/cgi-bin/emblfetch?AB070933) | ASIALOGLYCOPROTEIN RECEPTOR 1 (HEPATIC LECTIN H1) (ASGPR) (ASGP-R) |
| [H300001821](http://omad.operon.com/humanV3/transcript.php?what=H300001821) | [BLVRA](http://bioinfo.weizmann.ac.il/cards-bin/cardsearch.pl?search=BLVRA) | [NM_000712](http://srs.sanger.ac.uk/srsbin/cgi-bin/wgetz?-e+%5BREFSEQ-ID:NM_000712%5D) | [AC005189](http://www.ebi.ac.uk/cgi-bin/emblfetch?AC005189) | BILIVERDIN REDUCTASE A PRECURSOR (EC 1.3.1.24) (BILIVERDIN-IX ALPHA- REDUCTASE) |
| [H200001397](http://omad.operon.com/humanV3/transcript.php?what=H200001397) | [RAI17](http://bioinfo.weizmann.ac.il/cards-bin/cardsearch.pl?search=RAI17) | -- | [AB033050](http://www.ebi.ac.uk/cgi-bin/emblfetch?AB033050) | -- |
| [H300008401](http://omad.operon.com/humanV3/transcript.php?what=H300008401) | [TRAF3](http://bioinfo.weizmann.ac.il/cards-bin/cardsearch.pl?search=TRAF3) | [NM_003300](http://srs.sanger.ac.uk/srsbin/cgi-bin/wgetz?-e+%5BREFSEQ-ID:NM_003300%5D) | [U15637](http://www.ebi.ac.uk/cgi-bin/emblfetch?U15637) | TNF RECEPTOR ASSOCIATED FACTOR 3 (CD40 RECEPTOR ASSOCIATED FACTOR 1) (CRAF1) (CD40 BINDING PROTEIN) (CD40BP) (LMP1 ASSOCIATED PROTEIN) (LAP1) (CAP-1) |
| [H300022877](http://omad.operon.com/humanV3/transcript.php?what=H300022877) | [LILRB1](http://bioinfo.weizmann.ac.il/cards-bin/cardsearch.pl?search=LILRB1) | [NM_006669](http://srs.sanger.ac.uk/srsbin/cgi-bin/wgetz?-e+%5BREFSEQ-ID:NM_006669%5D) | [AF009221](http://www.ebi.ac.uk/cgi-bin/emblfetch?AF009221) | LEUKOCYTE IMMUNOGLOBULIN-LIKE RECEPTOR, SUBFAMILY B (WITH TM AND ITIM DOMAINS), MEMBER 1; LEUKOCYTE IMMUNOGLOBULIN-LIKE RECEPTOR 1; CD85 ANTIGEN |
| [H300018428](http://omad.operon.com/humanV3/transcript.php?what=H300018428) | [BID](http://bioinfo.weizmann.ac.il/cards-bin/cardsearch.pl?search=BID) | [NM_001196](http://srs.sanger.ac.uk/srsbin/cgi-bin/wgetz?-e+%5BREFSEQ-ID:NM_001196%5D) | [BC022072](http://www.ebi.ac.uk/cgi-bin/emblfetch?BC022072) | BH3 INTERACTING DOMAIN DEATH AGONIST (BID) |
| [H300022441](http://omad.operon.com/humanV3/transcript.php?what=H300022441) | -- | -- | [AL360143](http://www.ebi.ac.uk/cgi-bin/emblfetch?AL360143) | -- |
| [H200014949](http://omad.operon.com/humanV3/transcript.php?what=H200014949) | [HMOX1](http://bioinfo.weizmann.ac.il/cards-bin/cardsearch.pl?search=HMOX1) | [NM_002133](http://srs.sanger.ac.uk/srsbin/cgi-bin/wgetz?-e+%5BREFSEQ-ID:NM_002133%5D) | [X14782](http://www.ebi.ac.uk/cgi-bin/emblfetch?X14782) | HEME OXYGENASE 1 (EC 1.14.99.3) (HO-1) |
| [H200006902](http://omad.operon.com/humanV3/transcript.php?what=H200006902) | [TIEG](http://bioinfo.weizmann.ac.il/cards-bin/cardsearch.pl?search=TIEG) | [NM_005655](http://srs.sanger.ac.uk/srsbin/cgi-bin/wgetz?-e+%5BREFSEQ-ID:NM_005655%5D) | [AF050110](http://www.ebi.ac.uk/cgi-bin/emblfetch?AF050110) | TRANSFORMING GROWTH FACTOR-BETA-INDUCIBLE EARLY GROWTH RESPONSE PROTEIN 1 (TGFB-INDUCIBLE EARLY GROWTH RESPONSE PROTEIN 1) (TIEG-1) (KRUEPPEL-LIKE FACTOR 10) |
| [H200001600](http://omad.operon.com/humanV3/transcript.php?what=H200001600) | [NOTCH2](http://bioinfo.weizmann.ac.il/cards-bin/cardsearch.pl?search=NOTCH2) | [NM_024408](http://srs.sanger.ac.uk/srsbin/cgi-bin/wgetz?-e+%5BREFSEQ-ID:NM_024408%5D) | [U77493](http://www.ebi.ac.uk/cgi-bin/emblfetch?U77493) | NEUROGENIC LOCUS NOTCH HOMOLOG PROTEIN 2 PRECURSOR (NOTCH 2) (HN2) |
| [H300007970](http://omad.operon.com/humanV3/transcript.php?what=H300007970) | [ZFP36L1](http://bioinfo.weizmann.ac.il/cards-bin/cardsearch.pl?search=ZFP36L1) | [NM_004926](http://srs.sanger.ac.uk/srsbin/cgi-bin/wgetz?-e+%5BREFSEQ-ID:NM_004926%5D) | [BC018340](http://www.ebi.ac.uk/cgi-bin/emblfetch?BC018340) | BUTYRATE RESPONSE FACTOR 1 (TIS11B PROTEIN) (EGF-RESPONSE FACTOR 1) (ERF-1) |
| [H300019724](http://omad.operon.com/humanV3/transcript.php?what=H300019724) | [IFI30](http://bioinfo.weizmann.ac.il/cards-bin/cardsearch.pl?search=IFI30) | [NM_006332](http://srs.sanger.ac.uk/srsbin/cgi-bin/wgetz?-e+%5BREFSEQ-ID:NM_006332%5D) | [AF097362](http://www.ebi.ac.uk/cgi-bin/emblfetch?AF097362) | GAMMA-INTERFERON INDUCIBLE LYSOSOMAL THIOL REDUCTASE PRECURSOR (GAMMA-INTERFERON-INDUCIBLE PROTEIN IP-30) |
| [H200004653](http://omad.operon.com/humanV3/transcript.php?what=H200004653) | -- | -- | [AB033073](http://www.ebi.ac.uk/cgi-bin/emblfetch?AB033073) | -- |
| [H300012785](http://omad.operon.com/humanV3/transcript.php?what=H300012785) | [WARS](http://bioinfo.weizmann.ac.il/cards-bin/cardsearch.pl?search=WARS) | [NM_004184](http://srs.sanger.ac.uk/srsbin/cgi-bin/wgetz?-e+%5BREFSEQ-ID:NM_004184%5D) | [X67928](http://www.ebi.ac.uk/cgi-bin/emblfetch?X67928) | TRYPTOPHANYL-TRNA SYNTHETASE (EC 6.1.1.2) (TRYPTOPHAN--TRNA LIGASE) (TRPRS) (IFP53) (HWRS) |
| [H200010704](http://omad.operon.com/humanV3/transcript.php?what=H200010704) | [CPVL](http://bioinfo.weizmann.ac.il/cards-bin/cardsearch.pl?search=CPVL) | [NM_031311](http://srs.sanger.ac.uk/srsbin/cgi-bin/wgetz?-e+%5BREFSEQ-ID:NM_031311%5D) | [BC016838](http://www.ebi.ac.uk/cgi-bin/emblfetch?BC016838) | SERINE CARBOXYPEPTIDASE VITELLOGENIC-LIKE |
| [H200017278](http://omad.operon.com/humanV3/transcript.php?what=H200017278) | [SCO2](http://bioinfo.weizmann.ac.il/cards-bin/cardsearch.pl?search=SCO2) | [NM_005138](http://srs.sanger.ac.uk/srsbin/cgi-bin/wgetz?-e+%5BREFSEQ-ID:NM_005138%5D) | [AL021683](http://www.ebi.ac.uk/cgi-bin/emblfetch?AL021683) | SCO2 PROTEIN HOMOLOG, MITOCHONDRIAL PRECURSOR |
| [H200005078](http://omad.operon.com/humanV3/transcript.php?what=H200005078) | -- | [NM_006344](http://srs.sanger.ac.uk/srsbin/cgi-bin/wgetz?-e+%5BREFSEQ-ID:NM_006344%5D) | [D50532](http://www.ebi.ac.uk/cgi-bin/emblfetch?D50532) | MACROPHAGE LECTIN 2 (CALCIUM DEPENDENT) |
